# Plasma proteomic biomarkers as mediators or moderators for the association between poor cardiovascular health and white matter microstructural integrity: The UK Biobank study

**DOI:** 10.1002/alz.14507

**Published:** 2025-01-17

**Authors:** May A. Beydoun, Hind A. Beydoun, Nicole Noren Hooten, Zhiguang Li, Yi‐Han Hu, Michael F. Georgescu, Sharmin Hossain, Toshiko Tanaka, Mustapha Bouhrara, Christian A. Maino Vieytes, Marie T. Fanelli‐Kuczmarski, Lenore J. Launer, Michele K. Evans, Alan B. Zonderman

**Affiliations:** ^1^ Laboratory of Epidemiology and Population Sciences National Institute on Aging, NIA/NIH/IRP Baltimore Maryland USA; ^2^ VA National Center on Homelessness Among Veterans U.S. Department of Veterans Affairs Washington, DC USA; ^3^ Department of Management, Policy, and Community Health, School of Public Health University of Texas Health Science Center at Houston Houston Texas USA; ^4^ Department of Human Services (DHS) State of Maryland Baltimore Maryland USA; ^5^ Translational Gerontology Branch National Institute on Aging, NIA/NIH/IRP Baltimore Maryland USA; ^6^ Laboratory of Clinical Investigation National Institute on Aging, NIA/NIH/IRP Baltimore Maryland USA

**Keywords:** aging, cardiovascular health, diffusion‐weighted magnetic resonance imaging, Life's Essential 8, plasma proteomic biomarkers, white matter microstructural integrity

## Abstract

**INTRODUCTION:**

The plasma proteome's mediating or moderating roles in the association between poor cardiovascular health (CVH) and brain white matter (WM) microstructural integrity are largely unknown.

**METHODS:**

Data from 3953 UK Biobank participants were used (40–70 years, 2006–2010), with a neuroimaging visit between 2014 and 2021. Poor CVH was determined using Life's Essential 8 (LE8) and reversing standardized *z*‐scores (LE8*_z_*__rev_). The plasma proteome was examined as a potential mediator or moderator of LE8*_z_*__rev_’s effects on quantitative diffusion‐weighted magnetic resonance imaging (dMRI) metrics.

**RESULTS:**

LE8_z_rev_ was significantly associated with deteriorated WM microstructural integrity, as reflected by lower tract‐averaged fractional anisotropy (dMRI‐FA_mean_), (*β* ± standared error (SE): −0.00152 ± 0.0003, *p* < 0.001) and higher tract‐averaged orientation dispersion (dMRI‐OD_mean_), (*β* ± SE:+0.00081 ± 0.00017, *p* < 0.001). Ten strongly mediating plasma proteins of 1463 were identified, with leptin as the principal driver.

**DISCUSSION:**

Poor CVH is linked to poor WM microstructural integrity measures (lower FA_mean_ and higher OD_mean_), mostly mediated through leptin.

**Highlights:**

- Up to 3953 UK Biobank participants were selected for this study.
- Poor cardiovascular health (CVH) was determined using Life's Essential 8.
- The plasma proteome was examined as a potential mediator or moderator of poor CVH's effect on dMRI metrics.
- Ten plasma proteins were identified with strong mediating effects, with leptin being the principal driver.

## 1 BACKGROUND

Dementia is a leading cause of disability and mortality among aging people,^1^, ^2^ with Alzheimer's disease (AD) being the most frequently observed subtype.^3^ AD is a progressive neurodegenerative disorder with multifactorial etiology, causing temporal decrements in episodic memory and cognitive impairment.^4^ Risk factors for late‐onset AD include older age, family history, and genetics, with the apolipoprotein E (*APOE*) ε4 allele being a major genetic risk factor.^5^ The 2020 Lancet Commission, and its more recent 2024 update, identified 14 modifiable risk factors contributing to ≈40% of dementia cases.^6^, ^7^ Diet, physical activity, smoking, and sleep partially mediate some socio‐economic disparities in dementia risk.^8^ The American Heart Association's Life's Essential 8 (LE8), a composite measure of cardiovascular health (CVH), has recently been developed as an upgrade to Life's Simple 7 (LS7).^9^, ^10^ The LE8 score is based on four lifestyle factors, namely diet, physical activity, nicotine exposure, and sleep health, combined with biological factors, namely body mass index (BMI), blood pressure, blood cholesterol, and blood glucose, with higher scores indicating better CVH.^9^, ^10^ The LE8, like the LS7, has been proposed as a framework to identify factors that may affect dementia incidence among numerous other health outcomes.^8^, ^9^, ^10^, ^11^, ^12^, ^13^, ^14^, ^15^, ^16^ Given that dementia and AD are polygenic traits,^5^ at least one study showed that both CVH based on the LS7 and genetic risk for AD contribute to dementia risk.^17^ Studies on the plasma proteome and CVH were primarily targeted, although the plasma proteome has been linked to all‐cause dementia in large‐scale proteomics.^18^, ^19^, ^20^, ^21^, ^22^ Furthermore, sex differences were shown for both CVH (along with its components) and dementia incidence.^23^, ^24^

Magnetic resonance imaging (MRI) is a powerful tool for studying brain tissue alterations that may contribute to neuropsychiatric and neurodegenerative disorders, such as AD.^25^, ^26^, ^27^, ^28^, ^29^ Diffusion tensor imaging (DTI) is a prominent technique used to characterize microstructural changes in cerebral tissue.^30^ DTI indices, such as fractional anisotropy (FA) and mean diffusivity (MD), quantify the degree of anisotropy and overall diffusion of water within brain tissue^30^, ^31^ and have been used to study cerebral aging and degeneration,^32^, ^33^, ^34^, ^35^, ^36^, ^37^ showing variations in deterioration across different segments within specific tracts.^32^ DTI offers insights into brain microstructural changes, particularly in vulnerable areas like the frontal lobes.^33^ DTI can be a critical tool for identifying neural underpinnings of various psychopathologies.^34^ Although higher cerebral blood flow (CBF) is linked to better WM integrity preservation,^35^ obesity is associated with worsening of this brain health outcome, potentially contributing to cognitive and neuropsychiatric disorders.^36^ The latter finding was corroborated for other cardiovascular risk factors including hypertension, diabetes, and high cholesterol.^37^ More recently, multicomponent diffusion approaches like neurite orientation dispersion and density imaging (NODDI) have been introduced, providing additional biomarkers of cerebral tissue, such as neurite density index (NDI) and orientation dispersion index (ODI), beyond DTI metrics.^38^ DTI and NODDI can be placed under the umbrella of diffusion‐weighted MRI (dMRI).

Cognitive aging has been frequently linked with proteins such as C‐reactive protein (CRP), interleukin‐6 (IL‐6), tumor necrosis factor alpha (TNF‐α), apolipoprotein E (ApoE), and fibrinogen,^39^, ^40^, ^41^, ^42^ which can potentially mediate or mediate the associations between CVH and dementia‐related MRI outcomes through several mechanisms.^43^, ^44^, ^45^, ^46^ Similarly, ApoE is crucial in lipid transport and has been linked to AD pathology, including amyloid plaque accumulation and brain atrophy and impaired myelination, as well as poor CVH.^47^, ^48^ Therefore, poor CVH, characterized by elevated levels of these proteins, could result in the structural brain changes observed on MRI.

Nevertheless, the potential mediating and/or moderating effects of the plasma proteome on the relationship between poor CVH and dMRI‐based metrics is yet to be studied in large‐scale cohorts. In this original study, we comprehensively investigated the relationship of poor CVH (measured with LE8) with cerebral white matter (WM) microstructural integrity, assessed using DTI and NODDI dMRI metrics, among UK Biobank participants, with a focus on the potential mediating and moderating effects of the plasma proteome in this relationship.

## 2 METHODS

### 2.1 Database

The UK Biobank is a prospective study that included a little over 500,000 UK residents who were recruited between the years of 2006 and 2010, as long as their baseline ages ranged between 37 and 73 years.^49^ The study's goals and methodology are explained elsewhere.^49^ After being selected, participants were sent to one of 22 assessment sites (located within 25 miles) in England, Scotland, or Wales to complete touch‐screen and self‐administered questionnaires in addition to an in‐person interview.^49^ Phenotypic measurements and biological samples were acquired.^49^ A wide range of measurable exposures for various areas of interest were chosen for the UK Biobank questionnaire following a thorough assessment of prior observational studies, clinical trials, and demographic surveys, as well as collaboration with international experts. The present application number 77,963 was approved by the UK Biobank access management team, and ethical approval was also obtained from the National Institutes of Health's Institutional Review Board.

#### 2.1.1 Dementia exclusion criterion

Using linkage over time with electronic health records, including from hospitalizations, individuals whose age at the onset of dementia was younger than their initial age at baseline assessment were eliminated using algorithmically generated data for dementia occurrences (reported in fields 42,018 and 42,020).^50^

#### 2.1.2 dMRI phenotypic outcomes

In an ongoing secondary investigation that began in 2014 and had recruited 50,000 participants by October 2022, brain MRIs were conducted on a subset of UK Biobank participants at three different MRI locations around the study area.^51^, ^52^ Among several standard exclusion criteria, dementia, Parkinson's disease, and stroke patients were excluded from these brain imaging studies.^53^ Similar 3T Siemens Skyra scanners were used to collect all brain MRI data, which were then processed using published methods and documentation found at https://biobank.ctsu.ox.ac.uk/crystal/crystal/docs/brain_mri.pdf. The protocol is freely available at http://www.fmrib.ox.ac.uk/ukbiobank/protocol/V4_23092014.pdf.

**RESEARCH IN CONTEXT**

1. **Systematic review**: The authors of this work reviewed the literature relying mainly on PubMed searches. Cardiovascular health (CVH) as measured by Life's Essential 8 (LE8) composite score has been explored in a handful of studies in relation to various dementia traits and neuroimaging markers of dementia, including but not limited to diffusion‐weighted magnetic resonance imaging metrics. Despite evidence of an association between poor CVH and reduced white matter (WM) microstructural integrity, the mechanism underlying such association remains unclear. We therefore examined potential mediators and moderators of these relationships by considering the plasma proteome in a sample of ≤3953 participants of the UK Biobank study.
2. **Interpretation**: Our findings suggest that two neuroimaging metrics of WM microstructural integrity, namely global means of fractional anisotropy (FA_mean_) and orientation dispersion (OD_mean_), were significantly associated with poor CVH as measured by the LE8. Several plasma proteins mediated these relationships, the most notable being leptin, particularly for OD_mean_. These findings are consistent with the hypothesized link between poor CVH and early neuroimaging markers for dementia and contribute further to the evidence from epidemiologic studies as to the mechanism behind this relationship.
3. **Future directions**: Future research should explore those mediational relationships with repeated measures on FA and OD metrics as applied to comparable populations. Upon replication of our findings in a longitudinal setting, enhancing leptin sensitivity among others may be a therapeutic target that would improve WM microstructural integrity, an early marker of brain health.

In short, the DTI and NODDI metrics were among the imaging‐derived phenotypes (IDPs) that the UK biobank team made available to authorized researchers after analyzing WM tract‐averaged dMRI indices (see Online Supplementary Methods (OSM) 1, Appendix I, for additional information). DTI indices, including FA and MD, quantify the degree of anisotropy and overall diffusion of water within brain tissue, representing sensitive imaging biomarkers of neuronal health, such as axonal, myelin, dendritic, and synaptic integrity.^30^, ^31^ FA, which ranges from 0 to 1, is used to assess molecular displacement's directionality by diffusion; higher values suggest anisotropic diffusion, conferring a preferred direction. MD evaluates the average magnitude of molecular displacement by diffusion, with higher values suggesting more freely diffusing water.^30^, ^31^ DTI describes the distribution of diffusion displacements using a statistical mode.^30^, ^31^ Multicomponent diffusion approaches, especially the NODDI MRI approach, have been introduced for improvement in specificity. NODDI uses a multicompartmental model of water diffusion that incorporates intracellular volume fraction (ICVF) or NDI, that is, water within neurites, extracellular water, and a compartment that consist of isotropically diffusing water (ISOVF) from the cerebrospinal fluid volume, while also providing a measure of the  ODI.^38^ ISOVF and the OD indices are, in general, inversely related to brain health, whereas ICVF/NDI integrity is directly related to brain health, with higher values indicating greater neurite density or integrity.^54^ NODDI became rapidly popular and was shown to provide reliable and specific information about neurite density and dispersion in studies of aging and investigations of neurologic disorders.^55^, ^56^

Here we restricted the IDPs to the DTI and NODDI metrics that are currently accessible, such as FA, MD, ISOVF, ICVF, and OD indices throughout the WM tracts.^57^ Tract‐averaged global means of FA (FA_mean_), MD (MD_mean_), ISOVF (ISOVF_mean_), ICVF (ICVF_mean_), and OD (OD_mean_) were the main MRI outcomes of interest, and in most analyses, those were transformed into standardized z‐scores within the final selected sample. We note that tract‐specific values were considered outcomes of secondary importance; further information and results are summarized in OSM 1 (Appendix I) and Table S1.

#### 2.1.3 CVH, measured by LE8

The LE8 composite measure of CVH was recently coined by the American Heart Association and used as the primary exposure of interest in this investigation. Most importantly, the definition and scoring approach for quantifying CVH using the American Heart Association's LE8 score, is described in detail as applied in the National Health and Nutrition Examination Surveys (NHANES) from 2013 to 2018.^9^, ^10^ Details on how it was applied to the UK Biobank data are provided elsewhere.^8^, ^58^ In summary, criteria applied cover the lifestyle and biological sub‐domains of LE8.^9^, ^10^ For *lifestyle factors*, the metrics include diet, physical activity, nicotine exposure, and sleep health.^9^, ^10^ Diet is scored based on adherence to a Dietary Approaches to Stop Hypertension (DASH)–style eating pattern, with higher quantiles reflecting better CVH^9^, ^10^ and its application to UK Biobank data is summarized in OSM 2 (Appendix I). Physical activity is measured in minutes of moderate or vigorous activity per week, with a target of 150+ min for top scoring.^9^, ^10^ Nicotine exposure is evaluated based on smoking history, with current smokers scoring the lowest, and points deducted for living with an indoor smoker.^9^, ^10^ Sleep health is assessed based on average nightly sleep, with 7–9 h considered optimal.^9^, ^10^ In the *biological* sub‐domain, metrics include BMI, blood lipids, blood glucose, and blood pressure.^9^, ^10^ Each factor is assigned points based on measured values, with optimal ranges corresponding to the highest scores.^9^, ^10^ For example, a BMI under 25 kg/m^2^,^9^, ^10^ non‐HDL cholesterol under 130 mg/dL,^9^, ^10^ fasting blood glucose below 100 mg/dL,^9^, ^10^ and optimal blood pressure under 120/80 mmHg each score 100 points.^9^, ^10^ Each factor includes detailed gradations for suboptimal levels, and points may be subtracted for conditions such as drug‐treated lipid levels or diabetes.^9^, ^10^ This scoring system allows for a comprehensive evaluation of CVH based on both behaviors and clinical health metrics.

Typically, the overall LE8 score can vary from 0 to 800, where a higher number indicates improved CVH. The score in this study was *z*‐standardized and reverse coded, or LE8*_z_*__rev_ (multiplying the *z*‐scored LE8 score by −1), so that a higher score would indicate reduced CVH, and a unit rise would represent a 1 standard deviation (SD) decline in the LE8 score, as was done previously in an earlier study.^58^ A similar procedure was carried out for the individual LE8 biological sub‐score, which included the elements of BMI, blood lipids, blood glucose, and blood pressure, as well as the LE8 lifestyle sub‐score, which included the elements of diet, physical activity, smoking, and sleep.^58^

#### 2.1.4 OLINK proteomics

One of the components of the UK Biobank Pharma Proteomics Project (UKB‐PPP) was large‐scale plasma proteome research. Using the Olink Explore 1536 Proteomics platform, 54,306 plasma samples from individual visits by UK Biobank participants were examined. Using Proximity Extension Assay (PEA) technology, this platform measures 1472 protein analytes, which correspond to 1463 different proteins from panels related to inflammation, cancer, the cardiometabolic system, and the nervous system. A thorough description of the pre‐processing and standardization of the data as well as the UKB‐PPP implementation of PEA assays in this cohort is provided elsewhere.^59^ Log2 normalized protein expression (NPX) units were calculated to present the protein data (OSM 3, Appendix I). Plasma proteomic data were measured on samples collected during the baseline assessment visit (2006–2010).

#### 2.1.5 Covariates

Relevant covariates were considered potential confounders between the association of LE8 with dMRI metrics. These covariates included socio‐demographics, namely age, sex, household size as an ordinal variable, and race/ethnicity re‐coded in the main modeling part of the analyses into non‐White versus White from the original race/ethnicity variable coded as “White, Black, South Asian, and Others,” due to the limited sample size available. Baseline data in the UK Biobank were collected using a touch‐screen questionnaire, which included information on educational attainment (https://biobank.ndph.ox.ac.uk/showcase/field.cgi?id = 6138), re‐coded as 0 = Low, combining “CSEs/Equivalent”, “NVQ/HND/HNC/Equivalent”, and “Other professional qualifications”; 1 = Intermediate, combining “O Levels/GCSEs/Equivalent” and “A/AS Levels Equivalent”; 2 = Higher level or “College/University”, as was done in previous studies.^60^ The five categories for household income prior to taxation were 1 = “Less than £18,000,” 2 = “£18,000–£29,999,” 3 = “£30,000‐£51,999,” 4 = “52,000–£100,000,” and 5 = “ > £100,000.” Using national census data, Townsend Deprivation Index (TDI) ratings were calculated emphasizing owner occupation, car ownership, overcrowding in homes, and unemployment at the residential postcode level. Greater socioeconomic disadvantage is indicated by higher TDI scores.^61^ Consequently, a single socioeconomic status (SES) summary score was obtained by multiplying TDI by −1 to denote greater SES and combining it with *z*‐scores for household income and educational attainment. In addition, household size and time elapsed between baseline visit and the neuroimaging visit were considered among potential confounders. Finally, a portion of our analyses stratified by the AD Polygenic Risk Score (AD PRS), expressed as tertiles, as a secondary potential effect modifier that included variants in the *APOE* gene and was found to be highly correlated with *APOE* ε4 status (see OSM 4, Appendix I).

#### 2.1.6 Study sample selection

The UK Biobank sample included 502,268 adults who had consented for their data to be used at the time of analysis, with 353,089 having key covariates of interest, including LE8 total score, sub‐scores and component scores, AD PRS, and various socio‐demographic and socio‐economic factors (Figure S1, Appendix II). Only 29,481 additionally had neuroimaging phenotypic data, including dMRI outcomes of interest. A final sample of 3953 had relevant key variables, dMRI phenotypic measures, and plasma proteomic data, after excluding one prevalent dementia case that was not accounted for by the main exclusion criteria.

#### 2.1.7 Statistical methods

##### Descriptive analysis

We first conducted descriptive analyses by estimating means and proportions of the main research variables for the entire selected sample, accounting for the stratification by sex. The estimates were compared by sex using linear, logistic, or multinomial logit models. The software Stata 18.0, developed by StataCorp in College Station, TX, was utilized for conducting all analyses. All heatmaps were generated using various R software functions.

##### Exposure‐Outcome analyses: Multiple linear regression models

Second, exposure‐outcome relationships were examined within a selected analytic sample, focusing on global dMRI metrics mean values and LE8_z_rev_ as the main exposure. Confounders included age, sex, race/ethnicity, SES *z*‐score, household size, and time between initial assessment and MRI visit. Multiple linear regression models were used to assess the relationships of the LE8*_z_*__rev_ exposure on each outcome of FA_mean_, MD_mean_, ISOVF_mean_, ICVF_mean_, and OD_mean_, adjusting for all covariates. Heterogeneity across sex was tested and stratification was presented, whereas for AD PRS tertiles, stratification was presented only if *p* < 0.10 for any of these sub‐analyses. Secondary outcomes for DTI and NODDI metrics included tract‐specific FA, MD, ISOVF, ICVF, and OD metrics. The effect sizes of LE8*_z_*__rev_ on individual *z*‐scored DTI and NODDI metrics were determined using R software version 4.3.1. Statistically significant findings after Bonferroni correction at type I error of 0.05 were also plotted on the JHU‐MNI 152 brain image template using FSLEYES software (FSLeyes—FslWiki (ox.ac.uk). All effect sizes are available as raw data on github (https://github.com/baydounm/UKB‐paper13‐supplementarydata).

##### Exposure‐mediator analyses: Multiple linear regression models

Third, the LE8*_z_*__rev_ exposure was used as the main predictor in multiple linear regression models with 1463 plasma proteomic biomarkers as outcomes. The analysis included adjusting for specified covariates using Stata *parmby, qqval*, and *multproc* commands. A volcano plot was created with the R ggplot program to display the *p*‐values and effect sizes obtained from 1463 equations, emphasizing those that met the Bonferroni adjustment for multiple testing. The selected top hits had an effect size greater than 0.20 in absolute value, which corresponds to one‐fifth SD higher (or lower for a negative value) plasma protein per SD decrement in LE8 total score. This value is considered a cutoff point between very weak (or very small) and weak (or small) effect sizes when examining the relationship between two continuous variables, based on various criteria such as those used for Cohen's *d* (https://cran.r‐project.org/web/packages/effectsize/vignettes/interpret.html). This step was included only if the Bonferroni correction resulted in a substantial number of putative proteomic mediators and/or moderators, for example, more than 500.

##### Mediation‐moderation analysis: Four‐way decomposition models

Fourth, the chosen plasma proteins from the previous step were integrated into a four‐way decomposition model with LE8*_z_*__rev_ as the exposure variable. This method generally decomposes an overall effect of an exposure on an outcome through the effect of a third variable, which is a potential mediator that is allowed to interact with the exposure. Each global mean of DTI and NODDI metrics served as alternative outcome variables of interest. Each of the selected proteomic biomarkers was used as a potentially mediating variable in a multiple linear regression modeling approach for both the mediator and the outcome final equation. Moreover, these proteomic biomarkers were able to serve as potential moderators in the relationship, by interacting with the exposure in relation to the outcome variables. Further exploration of the four‐way decomposition is detailed in OSM 5 (Appendix I), with an additional technical appendix provided in Appendix III. Tabular layout was used with heatmap visualizations specifically for the chosen key mediators meeting Bonferroni correction and other selection criteria. They are categorized as “no mediation,” “consistent mediation” (where the controlled direct effect [CDE] is less than the total effect [TE] in absolute value and the pure indirect effect [PIE] has the same sign as CDE), and “inconsistent mediation” (where CDE is greater than TE in absolute value and PIE and CDE have opposite signs).^62^, ^63^, ^64^ The key mediators or moderators of the association between LE8*_z_*__rev_ and dementia features, including neuroimaging markers of dementia, are highlighted based on their function and connection to CVH risk factors, LS7, and LE8 in prior studies. More specifically, consistent mediation was determined using the criteria of PIE *p* < 0.05 and/or % PIE of TE is >20% as long as TE *p* < 0.05 and CDE < TE.

##### Post hoc principal components analyses and four‐way decomposition models

Fifth, if multiple selected plasma proteins were statistically significant mediators with a significant PIE at type I error of 0.05, they were included in a principal components analysis (PCA), a data reduction technique. This was done under the condition that the TE was also statistically significant at a 0.05 alpha level and there was a consistent mediation effect (CDE less than TE in absolute value, and PIE having the same sign as CDE and/or the TE) for a minimum of two of the five DTI and NODDI metrics. Multiple extracted components were orthogonally varimax rotated to facilitate interpretation. The number of extracted components was decided upon using the Kaiser rule, stating that components with an eigenvalue greater than 1 should be retained.^65^ The PCA scores (*z*‐scores) were predicted using regression and then incorporated into four‐way decomposition models to assess the extent of mediation and/or moderation by these components in the overall effect of LE8*_z_*__rev_ on various global dMRI outcomes that exhibited statistically significant association with LE8*_z_*__rev_ at a type I error of 0.05. Sex was the primary criterion used for stratification, whereas AD PRS, which included the key *APOE* gene variants, served as an additional secondary stratifying variable.

##### Proteome‐wide four‐way decomposition models and pathways analyses

Sixth, the supplemental materials present results of a four‐way decomposition across the proteome, specifically analyzing 1463 proteins, with a focus on plasma proteins showing statistically significant TE and PIE at a significance level of 0.05, irrespective of the sign of PIE, as was done in previous studies.^58^, ^66^, ^67^, ^68^ This analysis is followed by OLINK insight (https://olink.com/) and STRING (https://string‐db.org/) pathway analyses detailed in OSM 8 (Appendix I) and github repository: https://github.com/baydounm/UKB‐paper13‐supplementarydata).

##### Post hoc analyses for LE8 components and sub‐scores

Finally, another secondary analysis was conducted to visualize the top mediators, who shared several dMRI metrics and focused on consistent mediators, in relation to the main exposure, its lifestyle and biological sub‐scores, and its individual components. Several heatmaps were used, as needed, along with similar ordinary least square (OLS) models that adjusted for household size, age, sex, racial minority status, and SES, and time elapsed between the baseline assessment and the neuroimaging visit.

## 3 RESULTS

### 3.1 Descriptive analysis

Within the selected study sample of 3953 persons from the UK Biobank with complete neuroimaging, proteomics, and key covariate data, and who were dementia‐free at baseline, we observed variations by sex in parameters such as age, race, household size, SES index, and LE8 total and sub‐scores (Table 1). Women, most notably, exhibited higher LE8 total, lifestyle, and biological sub‐scores, indicating better CVH, coupled with a lower socioeconomic status, particularly in terms of educational attainment and household income. Among participants who did not withdraw consent for their data to be used and had complete data on TDI among others, those included in the final sample differed from those excluded by being younger, less likely to be female, less likely to belong to a racial minority group, and less likely to have an elevated TDI (data not shown).

**TABLE 1:** Study sample characteristics by sex: UK Biobank 2006–2021.

	Overall (*N* = 3953)	Men (N = 1853)	Women (*N* = 2100)	*p*_sex_
**Demographic**				
Baseline age, years, mean ± SE	54.66 ± 0.12	55.42 ± 0.18	53.99 ± 0.17	<0.001
Sex, % female	53.1%	–	–	–
Race/ethnicity				
White	96.8%	96.9%	96.7%	(Ref)
Black	0.7%	0.8%	0.7%	0.75
South Asian	0.9%	1.0%	0.8%	0.48
Other	1.6%	1.4%	1.9%	0.27
Non‐White, %	3.21%	3.13%	3.29%	0.78
Household size	2.602 ± 0.019	2.657 ± 0.029	2.555 ± 0.026	0.007
**Socio‐economic**				
***Education***				
Low	15.0%	15.7%	14.4%	0.66
Intermediate	35.2%	31.1%	38.8%	<0.001
High	33.3%	53.2%	46.8%	(Ref)
***Income***				
Less than £18,000	11.3%	8.8%	13.5%	<0.001
£18,000–£29,999	22.5%	21.0%	23.8%	0.15
£30,000–£51,999	29.2%	29.3%	29.2%	(Ref)
£52,000–£100,000	28.8%	31.4%	26.4%	0.049
greater than £100,000	8.2%	9.4%	7.1%	0.035
Townsend Deprivation Index, mean ± SE	−1.773 ± 0.045	−1.824 ± 0.0655	−1.728 ± 0.061	0.28
SES, mean ± SE	−0.010 ± 0.011	+0.038 ± 0.015	−0.053 ± 0.014	<0.001
**Life's essential 8 LE8), mean ± SE**				
Total score	531.1 ± 1.5	512.9 ± 2.0	547.2 ± 2.1	<0.001
LE8*_z_*__rev_	1.10e‐10 ± 0.0159	0.0895 ± 0.0238	−0.1728 ± 0.0221	<0.001
Lifestyle score	262.2 ± 0.9	257.0 ± 1.4	266.9 ± 1.3	<0.001
Diet component	35.1 ± 0.5	30.6 ± 0.7	39.1 ± 0.7	<0.001
Physical activity component	50.5 ± 0.5	52.0 ± 0.8	49.2 ± 0.7	<0.001
Smoking component	85.1 ± 0.4	82.9 ± 0.7	87.0 ± 0.5	<0.001
Sleep component	91.5 ± 0.3	91.4 ± 0.4	91.6 ± 0.3	0.82
Biological score	268.9 ± 1.0	255.9 ± 1.4	280.3 ± 1.5	<0.001
BMI component	74.1 ± 0.4	70.5 ± 0.6	77.2 ± 0.6	<0.001
Lipids component	52.0 ± 0.5	50.6 ± 0.7	53.2 ± 0.7	0.007
Glucose component	94.7 ± 0.2	94.1 ± 0.4	95.1 ± 0.3	0.036
Blood pressure component	48.2 ± 0.5	40.7 ± 0.7	54.7 ± 0.7	<0.001
**AD PRS**				
Tertile, %				
T1	33.3	33.6	33.1	__
T2	33.3	33.3	33.3	0.82
T3	33.3	33.1	33.5	0.71
**dMRI global measures (raw unstandardized metrics),** mean ± SE				
FA_mean_	+0.5615 ± 0.0003	0.5614 ± 0.0005	+0.5616 ± 0.0004	0.71
MD_mean_	+0.0007925 ± 5.36E‐07	+0.0007958 ± 7.92E‐07	+0.0007896 ± 7.22E‐07	<0.001
ISOVF_mean_	+0.0943 ± 0.0002	+0.0954 ± 0.0003	+0.0932 ± 0.0003	<0.001
ICVF_mean_	+0.6120 ± 0.0005	+0.6112 ± 0.0007	+0.6129 ± 0.0006	0.089
OD_mean_	+0.1277 ± 0.0002	+0.1277 ± 0.0003	+0.1277 ± 0.0002	0.93

*Notes*: No multiple imputation was carried out in this analysis. *p*‐value is associated with the parameter for sex in bivariate linear and multinomial logistic regression analyses, with the main outcome being a continuous or categorical characteristic, respectively. (Ref) is the referent category in the multinomial logistic regression model. Values are means ± SE or percentages.

Abbreviations: AD, Alzheimer's disease; BMI, body mass index; dMRI, diffusion‐weighted magnetic resonance imaging; FA, fractional anisotropy; ICVF, intracellular volume fraction; ISOVF, isotropically diffusing water; LE8, Life's Essential 8; MD, mean diffusivity; OD, orientation dispersion; PRS, polygenic risk score; Ref, referent category; SE, standard error; TE, total effect.

### 3.2 Exposure‐outcome analyses: Multiple linear regression models

Table 2 provides the results of the overall association of LE8*_z_*__rev_ with raw (i.e., unstandardized) global means of DTI and NODDI measures using similar models as above. Most notably, a 1 SD increase in LE8*_z_*__rev_ was associated with a *β* ± standard error (SE) of +0.00081 ± 0.00017 (*p* < 0.001) increase in OD_mean_ and a –0.00152 ± 0.0003 decrease in FA_mean_ in the overall selected sample (*p* < 0.001). Further analyses did not show heterogeneity of these relationships across sex or AD PRS. In fact, stratified analysis by sex indicates similar associations between LE8*_z_*__rev_ and FA_mean_ and OD_mean_ among males and females.

**TABLE 2:** Poor cardiovascular health as measured by Life's Essential 8 total score (*z*‐scored, reverse coded) and global diffusion weighted magnetic resonance imaging (dMRI, raw unstandardized metrics) markers, overall, by sex: UK Biobank 2006–2021.

	LE8 *z*‐score, reverse‐coded (LE8*_z_*__rev_); *β* ± SE	P_LE8_ *_z_*__rev_	P_sex × LE8_ *_z_*__rev_	P_ADPRS × LE8_ *_z_*__rev_
**dMRI outcomes, Y**				
**Overall, *N* =** **3953**				
FA_mean_	−0.00155 ± 0.0003	<0.001	0.74	0.32
MD_mean_	2.24‐07 ± 4.91E‐07	0.65	0.73	0.80
ISOVF_mean_	+0.00039 ± 0.00020	0.050	0.34	0.66
ICVF_mean_	+0.00023 ± 0.00046	0.62	0.95	0.50
OD_mean_	+0.00082 ± 0.00017	<0.001	0.28	0.77
**Men, *N* = 1,853**				
FA_mean_	−0.00165 ± 0.0005	0.001	__	0.52
MD_mean_	4.05E‐07 ± 7.46E‐07	0.59	**__**	0.97
ISOVF_mean_	+0.0006 ± 0.0003	0.042	**__**	0.96
ICVF_mean_	+0.0003 ± 0.0007	0.69	**__**	0.58
OD_mean_	+0.0010 ± 0.0003	<0.001	__	0.36
**Women, *N* = 2100**				
FA_mean_	−0.00146 ± 0.0004	0.001	__	0.38
MD_mean_	1.18E‐07 ± 6.61E‐07	0.86	**__**	0.51
ISOVF_mean_	+0.0002 ± 0.0003	0.45	**__**	0.68
ICVF_mean_	+0.0001 ± 0.0006	0.87	**__**	0.097
OD_mean_	+0.0007 ± 0.0002	0.003	__	0.51

*Note*: All linear regression models were adjusted for baseline age, sex, race/ethnicity (non‐White vs White), household size, SES *z*‐score, and time elapsed between baseline assessment visit and neuroimaging visit (days). Interaction between LE8_z_rev_ and AD PRS tertiles and between LE8_z_rev_ and sex were tested, by including a 2‐way interaction term in the main adjusted model. Values are adjusted regression coefficients *β* ± SE. 1 SD of LE8*_z_*__rev_ is equivalent to 93.1 point reduction in LE8 total score.

Abbreviations: AD, Alzheimer's disease; dMRI, diffusion‐weighted magnetic resonance imaging; FA, fractional anisotropy; ICVF, intracellular volume fraction; ISOVF, isotropically diffusing water; LE8, Life's essential 8; MD, mean diffusivity; OD, orientation dispersion; PRS, polygenic risk score; SE, Standard Error; SES, socioeconomic status; TE, total effect.

**FIGURE 1 alz14507-fig-0001:**
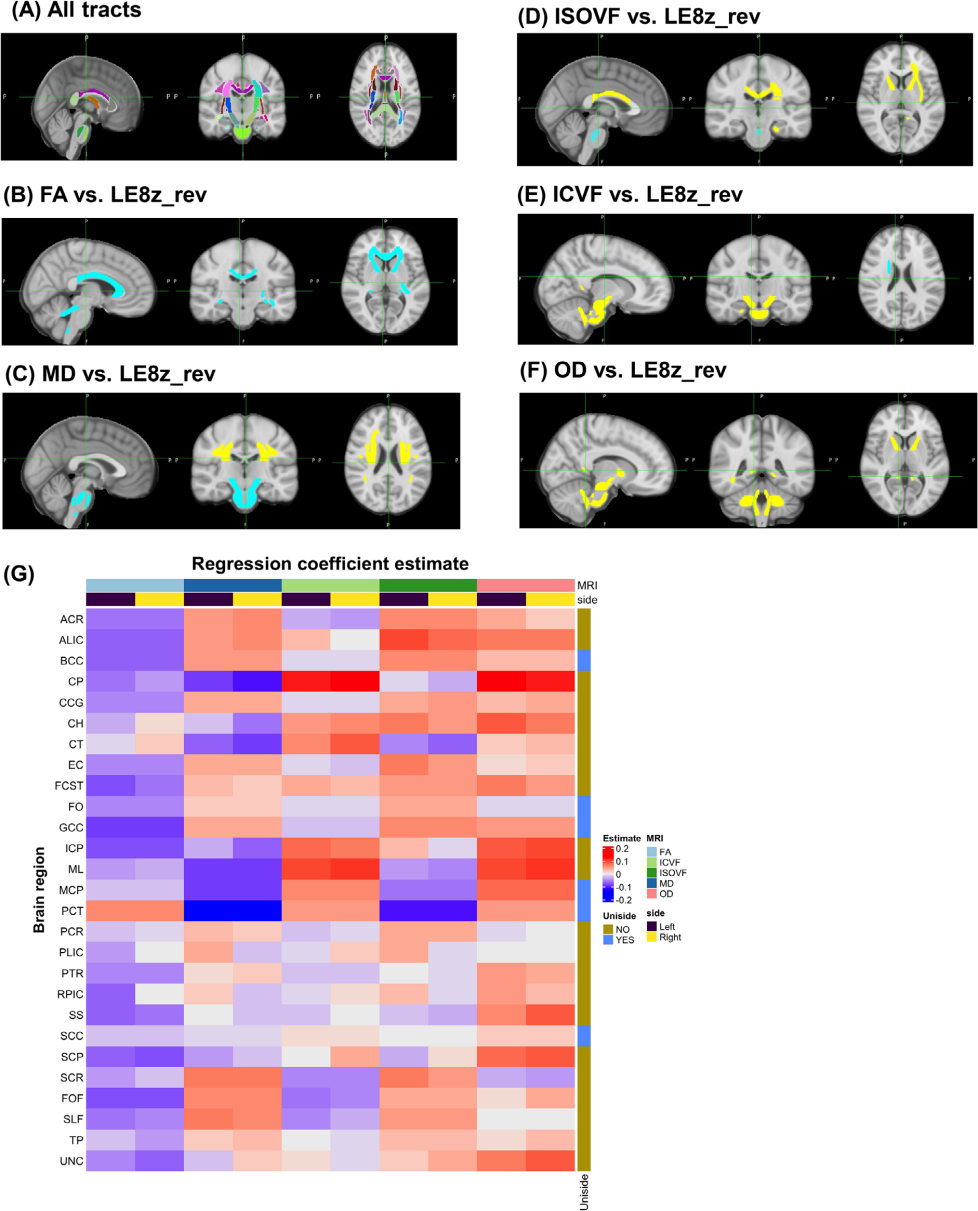
Poor cardiovascular health (LE8*_z_*__rev_total_) and tract‐specific and mean dMRI metrics (FA, MD, ICVF, ISOVF, OD, standardized z‐scores) in final selected sample (*N* = 3953), multiple linear regression models: UK Biobank 2006–2021. Based on a series of linear regression models adjusted for age, sex, race (non‐White vs White), time elapsed from baseline to neuroimaging visit (days), and household size. Tract‐specific FA, MD, ISOVF, ICVF, and OD are standardized z‐scores, as is the reverse‐coded LE8 total score. Effect sizes are plotted on a heat map to highlight effect sizes (G), whereas standard Montreal Neurological Institute (MNI) brain images display effect sizes on the brain regions/tracts only when *p*corr < 0.05. Light blue (or yellow) color is for effect sizes in absolute values ≥0.04 and dark blue (or red) are for effect sizes in absolute value ≥0.03 but <0.04. Light blue/dark blue are for inverse associations and yellow/red are for positive associations. Those are highlighted rows in supplementary datasheet 1 (B) is for FA and (C) is for MD; (D) is for ISOVF; (E) is for ICVF; (F) is for OD; (A) displays the entire JHU FA skeleton with different colors. Better WM microstructural integrity is linked to higher FA and ICVF and lower MD, ISOVF, and OD. FA, fractional anisotropy; ICVF, intra‐cellular volume fraction; ISOVF, volume fraction of Gaussian isotropic diffusion; MD, mean diffusivity; NODDI, neurite orientation dispersion and density imaging; OD orientation dispersion index.

Poor CVH measured by LE8*_z_*__rev_ was linked to significant decrements in WM microstructural integrity, by being associated with lower *z*‐scored tract‐specific FA and greater tract‐specific OD (>10 tracts of 48) of each of these metrics, after adjusting for relevant confounders as, depicted in Figure 1 (see Covariates section). Among tracts that survived Bonferroni correction of OD versus LE8*_z_*__rev_, we observed a positive association in ALIC (L/R), CP (L/R), CH (L/R), FCST (L), ICP (L/R), ML (L/R), MCP, SS (R), SCP (L/R), and UNC (L/R), whereas for FA versus LE8*_z_*__rev_ we observed inverse associations in ACR (L/R), ALIC (L/R), BCC, CP (L), FCST (L/R), GCC, ICP (L/R), RPIC (L), SS (L), SCP (L/R), FOF (R), and UNC (R) (see Appendix I, Table S1 for abbreviations; and supplementary datasheet 1 [Appendix IV] for details).

### 3.3 Exposure‐mediator analyses: Multiple linear regression models

**FIGURE 2 alz14507-fig-0002:**
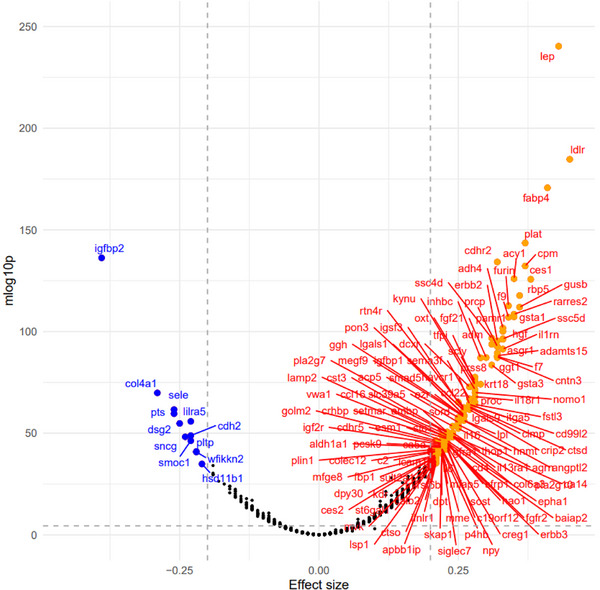
Volcano plot of plasma proteomic biomarkers (standardized *z*‐scored) in relation to LE8 reverse‐coded z‐scored total score: UK Biobank 2006–2010. Based on a series of multiple linear regression models, with main predictor being LE8 toal score (*z*‐scored, reverse‐coded by multiplying by −1) and the outcome being each of 1463 plasma proteomic biomarkers (Log2 transformed, *z*‐scored). The *y*‐axis is the predictor's associated *p*‐value on a –log10 scale and the *x*‐axis is the *β* coefficient (effect of LE8 exposure on standardized *z*‐scores of plasma proteomic markers) from the multiple linear regression models. An estimate with a Bonferroni corrected *p*‐value < 0.05 and an absolute estimate >0.20 is marked by the plasma proteomic marker abbreviation (see UK Biobank showcase URL: https://biobank.ndph.ox.ac.uk/showcase/). Selected proteins (*k* = 147) for further mediation analysis have a corrected *p*‐value < 0.05 and a point estimate >0.20 in absolute value (red). Dashed vertical lines are set at −0.20 and +0.20 effect sizes. Dashed horizontal line is set at the Bonferroni corrected *p*‐value. Details are provided on github: https://github.com/baydounm/UKB‐paper13‐supplementarydata. See list of abbreviations for protein abbreviations: mlog10p, −log_10_(*p*‐value); LE8, Life's Essential 8.

We ran 1463 multiple ordinary least square regression models to analyze the connections between LE8*_z_*__rev_ and each plasma protein, with the plasma protein being the main outcome. The models included age, sex, race/ethnicity, SES *z*‐score, household size, time elapsed, the key exposure of interest (LE8_z_rev_), and each plasma protein of the plasma proteome as the outcome (the potential mediators between LE8*_z_*__rev_ and DTI/NODDI metrics). LE8*_z_*__rev_ significantly predicted 844 plasma proteins after Bonferroni correction. Of those, only 147 showed a significant effect size β of either less than −0.20 or greater than +0.20, indicating a shift of 1/5 SD in plasma protein for each SD increase in LE8*_z_*__rev_, as illustrated in Figure 2. The protein LDLR (low‐density lipoprotein receptor) showed the highest positive effect size in Figure 2, followed by LEP (leptin) and FABP4 (fatty acid binding protein 4). Conversely, the largest inverse effect size was found for IGFPB2 (insulin‐like factor binding protein 2). These 147 proteins that were strongly predicted by the main exposure (LE8*_z_*__rev_) were incorporated into a four‐way decomposition model using LE8*_z_*__rev_ as the exposure and each of the five global means DTI and NODDI measurements (FA_mean_, MD_mean_, ICVF_mean_, ISOVF_mean_, and OD_mean_) as the outcomes.

### 3.4 Mediation‐moderation analysis: Four‐way decomposition models

**FIGURE 3 alz14507-fig-0003:**
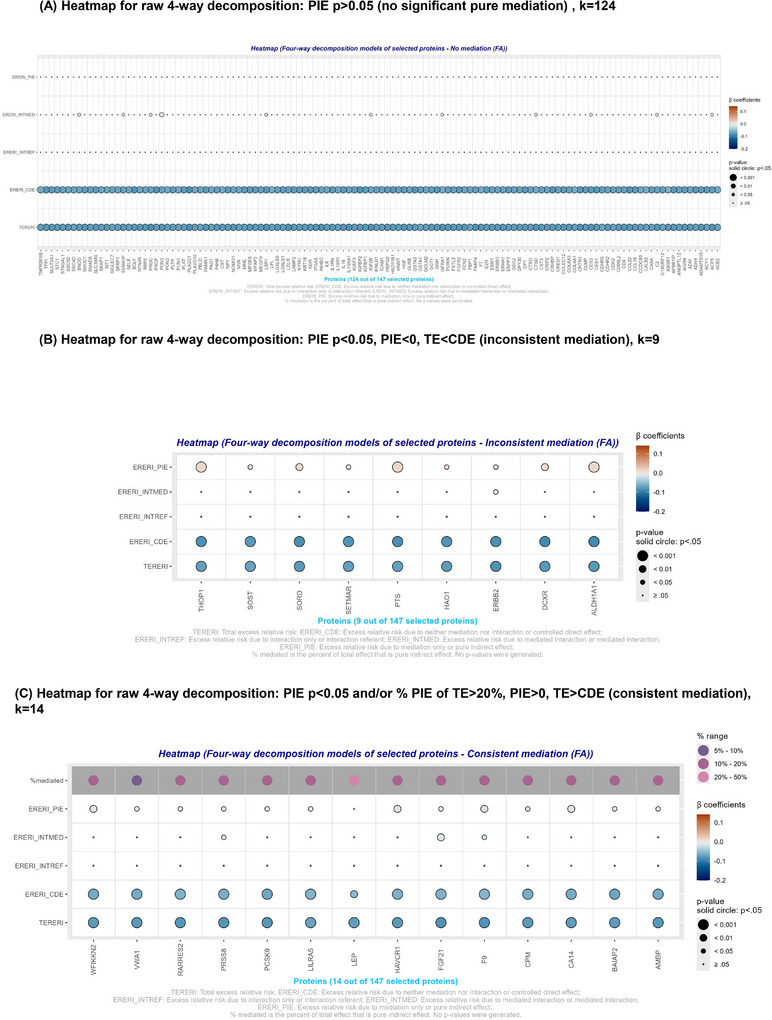
Four‐way decomposition of the association between poor cardiovascular health as measured by LE8 total score (*z*‐scored, reverse coded) and FA_mean_ (*z*‐scored) by the selected plasma proteomic biomarkers (*k* = 147): UK Biobank 2006–2021. (A) Heatmap for four‐way decomposition showing no significant pure mediation for 124 proteins: PIE *p* > 0.05. (B) Heatmap for four‐way decomposition showing inconsistent mediation for 9 proteins: PIE *p* < 0.05, PIE < 0, TE < CDE. (C) Heatmap for four‐way decomposition showing consistent mediation for 14 proteins: PIE *p* < 0.05 and/or % PIE of TE >20%, PIE > 0, and TE > CDE. Effects could range from 0 to 0.5. *p < 0.05; **p < 0.010; ***p < 0.001. Protein abbreviations are found at https://www.ncbi.nlm.nih.gov/gene/. ERERI_CDE, excess relative risk due to neither mediation nor interaction or controlled direct effect; ERERI_INTMED, excess relative risk due to mediated interaction or mediated interaction; ERERI_INTREF, excess relative risk due to interaction only or interaction referent; ERERI_PIE, excess relative risk due to mediation only or pure indirect effect; FA, fractional anisotropy; TERERI, total excess relative risk.

**FIGURE 4 alz14507-fig-0004:**
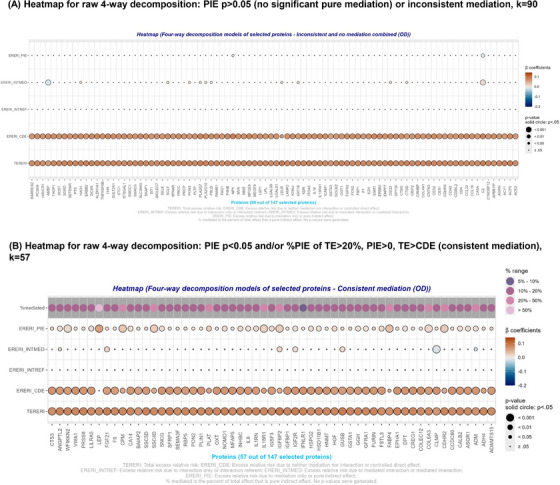
Four‐way decomposition of the association between poor cardiovascular health as measured by LE8 total score (*z*‐scored, reverse coded) and OD_mean_ (*z*‐scored) by the selected plasma proteomic biomarkers (*k* = 147): UK Biobank 2006–2021. (A) Heatmap for four‐way decomposition showing no significant or inconsistent mediation for 90 proteins (see A and B of Figure 3). (B) Heatmap for four‐way decomposition showing consistent medition for 57 proteins: PIE *p* < 0.05 and/or % PIE of TE >20%, PIE > 0, TE > CDE. Effects could range from 0 to 0.5. *p < 0.05; **p < 0.010; ***p < 0.001. Protein abbreviations are found at https://www.ncbi.nlm.nih.gov/gene/. ERERI_CDE, excess relative risk due to neither mediation nor interaction or controlled direct effect; ERERI_INTMED, excess relative risk due to mediated interaction or mediated interaction; ERERI_INTREF, excess relative risk due to interaction only or interaction referent; ERERI_PIE, excess relative risk due to mediation only or pure indirect effect; OD, orientation dispersion; TERERI, total excess relative risk.

Four‐way decomposition[Fig alz14507-fig-0001], [Fig alz14507-fig-0002] model findings applied to the selected proteins with strong relationship with the primary exposure (*k* = 147) are presented in Table 3, Figures 3 and 4, and Appendix V (supplementary datasheet 2). Results in Figures 3 and 4 were categorized as: (A) PIE did not result in any mediation (*p* > 0.05); (B) statistically significant PIE, reflecting pure mediation, at type I error of 0.05, with |CDE| > |TE| and PIE sign opposite to that of TE (*p* < 0.05); and (C) statistically significant PIE, reflecting pure mediation, at type I error of 0.05 with |CDE| < |TE| and PIE sign identical to TE (*p* < 0.05) and/or % PIE of TE >20%, for each of FA_mean_ and OD_mean_. Table 3 presents the comprehensive results for consistent mediation that were found to be in common between FA_mean_ and OD_mean_, whereas Appendix V (supplementary datasheet 2) has the full set of findings that are depicted in Figures 3 and 4. Although most of the selected proteins were not significant mediators for both outcomes, 10 plasma proteins (Table 3) consistently mediated the association between LE8*_z_*__rev_ and both FA_mean_ and OD_mean_, whereas findings for OD_mean_ indicated that 57 plasma proteins were among those consistent mediators that were strongly associated with the LE8*_z_*__rev_ exposure, the latter being indicative of poor CVH. Among those consistent mediators that were shared between the two outcomes, leptin was the strongest mediator, explaining >80% of the LE8*_z_*__rev_ versus OD_mean_ association, resulting in a markedly attenuated CDE (*p* > 0.05), and explaining >20% of the TE of LE8*_z_*__rev_ on FA_mean_ (Table 3, Figures 3 and 4 and supplementary datasheet 2 (Appendix V)). Similarly, CPM explained >30% of the TE of LE8*_z_*__rev_ on OD_mean_ and ≈20% of the TE of LE8*_z_*__rev_ on FA_mean_. When examining Figures 3C and 4B, very few of the four‐way decomposition findings indicated that a plasma protein may have acted as a moderator in addition to being a mediator (i.e., INTMED) in the overall effect of LE8*_z_*__rev_ on FA_mean_ and OD_mean_, respectively, with a consistent mediated interaction found for FGF21 for both metrics.[Table alz14507-tbl-0003]

**TABLE 3 alz14507-tbl-0003:** Four‐way decomposition of the association between poor cardiovascular health (LE8, *z*‐scored, reverse coded) and global diffusion‐weighted magnetic resonance imaging markers (dMRI) through selected plasma proteomic biomarkers (*k* = 10; *N*_max _= 3953, with consistent mediation; in common between FA_mean_ and OD_mean_ (both standardized *z*‐scored): UK Biobank 2006–2021.

	FA_Mean			OD_Mean			
FOURWAYDECOMP	Beta	SE	P	Beta	SE	*p*	PROTEIN
te	−**0.07356**	**0.015984**	**<0.001**	**0.07551**	**0.017007**	**<0.001**	LEP
cde	−0.0521	0.017582	0.003	0.016245	0.018633	0.383	LEP
intref	−7.41E‐06	0.000127	0.953	−2.03E‐06	3.61E‐05	0.955	LEP
intmed	−0.00468	0.006179	0.448	−0.00128	0.006547	0.845	LEP
pie	−**0.01677**	**0.008763**	**0.056**	**0.060551**	**0.009426**	**<0.001**	LEP
p_cde	0.70823	0.152513	<0.001	0.215131	0.211199	0.308	LEP
p_intref	0.000101	0.001722	0.953	−2.7E‐05	0.000479	0.955	LEP
p_intmed	0.063689	0.080681	0.43	−0.01699	0.087977	0.847	LEP
p_pie	**0.22798**	**0.127625**	**0.074**	**0.801888**	**0.214866**	**<0.001**	**LEP**
te	**−0.07653**	**0.015583**	**<0.001**	**0.079535**	**0.01658**	**<0.001**	CPM
cde	**−0.06134**	**0.016341**	**<0.001**	**0.052125**	**0.017363**	**0.003**	CPM
intref	−1.59E‐06	2.54E‐05	0.95	2.58E‐07	1.65E‐05	0.988	CPM
intmed	−0.00054	0.005178	0.917	8.77E‐05	0.005502	0.987	CPM
pie	**−0.01465**	**0.006237**	**0.019**	**0.027322**	**0.006684**	**<0.001**	CPM
p_cde	**0.801507**	**0.104464**	**<0.001**	**0.655371**	**0.11797**	**<0.001**	CPM
p_intref	2.08E‐05	0.000332	0.95	3.25E‐06	0.000208	0.988	CPM
p_intmed	0.007064	0.067323	0.916	0.001103	0.069126	0.987	CPM
p_pie	**0.191408**	**0.090753**	**0.035**	**0.343523**	**0.110692**	**0.002**	CPM
te	**−0.08144**	**0.015641**	**<0.001**	**0.078361**	**0.016682**	**<0.001**	F9
cde	**−0.0565**	**0.016123**	**<0.001**	**0.060702**	**0.017226**	**<0.001**	F9
intref	−7.75E‐06	0.000434	0.986	1.80E‐06	0.000101	0.986	F9
intmed	−0.01025	0.004953	0.038	0.002379	0.005271	0.652	F9
pie	**−0.01468**	**0.005565**	**0.008**	**0.015278**	**0.005944**	**0.01**	F9
p_cde	**0.693763**	**0.101948**	**<0.001**	**0.774649**	**0.106903**	**<0.001**	F9
p_intref	9.52E‐05	0.005326	0.986	2.29E‐05	0.001285	0.986	F9
p_intmed	0.125908	0.05939	0.034	0.030362	0.065922	0.645	F9
p_pie	**0.180234**	**0.075895**	**0.018**	**0.194966**	**0.085732**	**0.023**	F9
te	**−0.08284**	**0.015357**	**<0.001**	**0.087067**	**0.016377**	**<0.001**	FGF21
cde	**−0.05844**	**0.015944**	**<0.001**	**0.061031**	**0.017001**	**<0.001**	FGF21
intref	−2.9E‐05	0.000649	0.965	3.22E‐05	0.000731	0.965	FGF21
intmed	**−0.01374**	**0.004397**	**0.002**	**0.01547**	**0.004695**	**0.001**	FGF21
pie	**−0.01063**	**0.004966**	**0.032**	**0.010534**	**0.005291**	**0.046**	FGF21
p_cde	**0.705473**	**0.091013**	**<0.001**	**0.700966**	**0.092776**	**<0.001**	FGF21
p_intref	0.000346	0.007832	0.965	0.00037	0.008389	0.965	FGF21
p_intmed	**0.16586**	**0.056373**	**0.003**	**0.177672**	**0.058116**	**0.002**	FGF21
p_pie	0.128322	0.06442	0.046	0.120992	0.064835	0.062	FGF21
te	**−0.08017**	**0.015436**	**<0.001**	**0.083943**	**0.016485**	**<0.001**	PRSS8
cde	**−0.06173**	**0.015905**	**<0.001**	**0.064504**	**0.016988**	**<0.001**	PRSS8
intref	−8.62E‐06	0.000414	0.983	8.73E‐06	0.00042	0.983	PRSS8
intmed	−0.00822	0.004108	0.045	0.008336	0.004385	0.057	PRSS8
pie	**−0.01021**	**0.004728**	**0.031**	**0.011094**	**0.005051**	**0.028**	PRSS8
p_cde	**0.769981**	**0.083457**	**<0.001**	**0.768424**	**0.085237**	**<0.001**	PRSS8
p_intref	0.000108	0.005164	0.983	0.000104	0.005	0.983	PRSS8
p_intmed	0.102576	0.051465	0.046	0.099308	0.052349	0.058	PRSS8
p_pie	**0.127335**	**0.063974**	**0.047**	**0.132164**	**0.06564**	**0.044**	PRSS8
te	**−0.07009**	**0.015455**	**<0.001**	**0.077944**	**0.016484**	**<0.001**	LILRA5
cde	**−0.05812**	**0.015637**	**<0.001**	**0.065044**	**0.016683**	**<0.001**	LILRA5
intref	−3.69E‐06	0.000137	0.978	2.65E‐06	9.98E‐05	0.979	LILRA5
intmed	−0.00227	0.003724	0.542	0.001675	0.004001	0.675	LILRA5
pie	−0.00969	0.003959	0.014	0.011223	0.004265	0.009	LILRA5
p_cde	**0.829243**	**0.081292**	**<0.001**	**0.83449**	**0.078086**	**<0.001**	LILRA5
p_intref	5.26E‐05	0.001947	0.978	0.000034	0.00128	0.979	LILRA5
p_intmed	0.032397	0.052169	0.535	0.02149	0.05061	0.671	LILRA5
p_pie	**0.138308**	**0.064028**	**0.031**	**0.143986**	**0.062455**	**0.021**	LILRA5
te	**−0.07527**	**0.01524**	**<0.001**	**0.079059**	**0.016251**	**<0.001**	CA14
cde	**−0.06408**	**0.015567**	**<0.001**	**0.066207**	**0.016597**	**<0.001**	CA14
intref	−4.21E‐06	9.85E‐05	0.966	5.56E‐06	0.00013	0.966	CA14
intmed	−0.00153	0.003322	0.645	0.002022	0.003543	0.568	CA14
pie	**−0.00965**	**0.003701**	**0.009**	**0.010824**	**0.003952**	**0.006**	CA14
p_cde	**0.851323**	**0.069825**	**<0.001**	**0.837438**	**0.071974**	**<0.001**	CA14
p_intref	0.000056	0.001308	0.966	7.03E‐05	0.001641	0.966	CA14
p_intmed	0.020358	0.043774	0.642	0.02558	0.044405	0.565	CA14
p_pie	**0.128263**	**0.055452**	**0.021**	**0.136912**	**0.057171**	**0.017**	CA14
te	**−0.07776**	**0.015282**	**<0.001**	**0.079085**	**0.016287**	**<0.001**	VWA1
cde	**−0.06535**	**0.015558**	**<0.001**	**0.06881**	**0.016594**	**<0.001**	VWA1
intref	−1.01E‐05	0.000313	0.974	4.08E‐06	0.000127	0.974	VWA1
intmed	−0.00485	0.003486	0.164	0.00196	0.003704	0.597	VWA1
pie	−0.00755	0.003658	0.039	0.008311	0.003904	0.033	VWA1
p_cde	**0.840384**	**0.068452**	**<0.001**	**0.870077**	**0.069483**	**<0.001**	VWA1
p_intref	0.00013	0.004025	0.974	5.17E‐05	0.001601	0.974	VWA1
p_intmed	0.062422	0.044654	0.162	0.024789	0.046355	0.593	VWA1
p_pie	0.097065	0.050728	0.056	0.105083	0.053889	0.051	VWA1
te	**−0.07776**	**0.015282**	**<0.001**	**0.068175**	**0.014994**	**<0.001**	BAIAP2
cde	**−0.06535**	**0.015558**	**<0.001**	**0.058109**	**0.0152**	**<0.001**	BAIAP2
intref	−1.01E‐05	0.000313	0.974	1.91E‐06	8.56E‐05	0.982	BAIAP2
intmed	−0.00485	0.003486	0.164	0.001283	0.003195	0.688	BAIAP2
pie	**−0.00755**	**0.003658**	**0.039**	**0.008782**	**0.003536**	**0.013**	BAIAP2
p_cde	**0.840384**	**0.068452**	**<0.001**	**0.852348**	**0.072394**	**<0.001**	BAIAP2
p_intref	0.00013	0.004025	0.974	0.000028	0.001256	0.982	BAIAP2
p_intmed	0.062422	0.044654	0.162	0.018815	0.046378	0.685	BAIAP2
p_pie	**0.097065**	**0.050728**	**0.056**	**0.128809**	**0.059092**	**0.029**	BAIAP2
te	**−0.07687**	**0.015335**	**<0.001**	**0.082398**	**0.016388**	**<0.001**	WFIKKN2
cde	**−0.0641**	**0.015518**	**<0.001**	**0.061793**	**0.016539**	**<0.001**	WFIKKN2
intref	−1.04E‐06	0.00014	0.994	3.19E‐06	0.000432	0.994	WFIKKN2
intmed	−0.00199	0.003122	0.524	0.00612	0.003353	0.068	WFIKKN2
pie	**−0.01077**	**0.003429**	**0.002**	**0.014482**	**0.003711**	**<0.001**	WFIKKN2
p_cde	**0.833949**	**0.064555**	**<0.001**	**0.749932**	**0.073507**	**<0.001**	WFIKKN2
p_intref	1.35E‐05	0.001825	0.994	3.87E‐05	0.00524	0.994	WFIKKN2
p_intmed	0.025872	0.040168	0.52	0.074276	0.041055	0.07	WFIKKN2
p_pie	**0.140165**	**0.052507**	**0.008**	**0.175753**	**0.056499**	**0.002**	WFIKKN2

*Note*: Names of the genes/proteins can be found at: https://www.ncbi.nlm.nih.gov/gene. Tereri and ereri_cde are interpreted as Log_e_(hazard ratios). 1 SD of LE8_z_rev_ is equivalent to 93.1 point reduction in LE8 total score.

Abbreviations: ERERI_CDE, excess relative risk due to neither mediation nor interaction or controlled direct effect; ERERI_INTMED, excess relative risk due to mediated interaction or mediated interaction; ERERI_INTREF, excess relative risk due to interaction only or interaction referent; ERERI_PIE, excess relative risk due to mediation only or pure indirect effect; FA, fractional anisotropy; LE8, Life's Essential 8; OD, orientation dispersion; p_CDE, proportion of total effect that is controlled direct effect; p_INTMED, proportion of total effect that is mediated interaction; p_INTREF, proportion of total effect that is interaction referent; p_PIE, proportion of total effect that is pure indirect effect; SE, standard error; TERERI, total excess relative risk.

### 3.5 Post hoc principal components analyses and four‐way decomposition models

A PCA was carried out in which a reduced number of dimensions were extracted using the Kaiser rule (eigenvalue > 1) and was applied to those top 10 significant proteomic mediators belonging to group C (i.e., consistent mediators) that were in common between FA_mean_ and OD_mean_, (details provided in OSM 5 and Table S2, Appendix I). This resulted in three PCs (PC1, PC2, and PC3) (Figure S2, Appendix VI). PC1 reflected the largest portion of the variance (≈6 proteins with factor loadings >0.20, % variance explained = 32.2%), whereas PC2 and PC3 reflected smaller proportions of the variance explained (14% and 11%, respectively). When a four‐way decomposition model was carried out for poor CVH versus FA_mean_, adjusting for the same set of covariates as above, PC1 explained over half of the LE8*_z_*__rev_‐FA_mean_ effect within the lowest AD PRS tertile and ≈25% of the TE overall (*p* < 0.05 for both PIEs), with similar patterns observed for PC2 overall and within the first two AD PRS groups. Around one‐third of the TE of LE8*_z_*__rev_ on FA_mean_ among women was explained by PC2. No significant mediation was detected for PC3 (Appendix VII, Figure S3). When it came to OD_mean_, findings were slightly distinct from those of FA_mean_ (Appendix VII, Figure S3). In fact, in the overall sample, all three PCs acted as statistically significant mediators, explaining 35%–50% of the TE of LE8*_z_*__rev_ on OD_mean_, with marked attenuation in the CDE (*p* > 0.05) compared with the TE observed for PC1 and PC2. Most notably, all three PCs explained each >30% of the TE of LE8*_z_*__rev_ on OD_mean_ within the higher AD PRS tertile.[Fig alz14507-fig-0003], [Fig alz14507-fig-0004]

### 3.6 Proteome‐wide four‐way decomposition models and pathways analyses

Furthermore, OLINK Insight and STRING analyses findings for all key mediators are summarized in Appendix I (OSM 7), as well as in Appendices VIII, IX, XA, XB and XI. Based on pathway analyses provided in detail in Figure S4 (Appendix VIII) and supplementary datasheet 3 (Appendix IX), 212 plasma proteins were mediators between poor CVH and FA_mean_, and 142 plasma proteins were mediators between poor CVH and OD_mean_, with 313 being shared between the two metrics. A total of 1008 pathways were identified based on the OLINK insight pathway analysis for mediators between poor CVH and FA_mean_ and/or OD_mean_. For FA_mean_ alone, 821 pathways were identified, whereas for OD_mean_, 525 unique pathways were identified. These pathways belong to the immune system, signal transduction, metabolism, disease, protein metabolism, hemostasis, apoptosis, cellular response to stress, and membrane trafficking. STRING analysis results are presented in Figure S4D,E, focusing on the largest cluster uncovered for FA_mean_ and OD_mean_ four‐way decomposition findings. Gene Ontology (GO) biological, molecular, and cellular components pathways for each of these clusters were obtained (see also: https://github.com/baydounm/UKB‐paper13‐supplementarydata). For OD_mean_, the largest cluster was automatically labeled as “cytokine‐cytokine receptor interaction,” whereas several GO pathways identified as being the strongest with lowest false discovery rate (FDR) were identified. For FA_mean_, clear patterns emerged regarding the strongest and most significant GO pathways.

### 3.7 Post hoc analyses for LE8 components and sub‐scores

Findings for the post hoc association between LE8 components and sub‐scores with each of the 10 key mediators are stored in Appendix XII (Figure S6) and Appendix XIII (supplementary datasheet 4). Most notably, although 7 of the 10 plasma proteins were increased with poorer CVH, the reverse was the case for carbonic anhydrase 14 (CA14) and WAP, Follistatin/Kazal, Immunoglobulin, Kunitz, and Netrin domain‐containing 2 (WFIKKN2), with the strongest correlations found with LE8*_z_*__rev_total_, LE8*_z_*__rev_biological_, and LE8*_z_*__rev_BMI_. Detailed Output, code and data sets used for heatmaps among others are provided in: https://github.com/baydounm/UKB‐paper13‐supplementarydata.

## 4 DISCUSSION

The present UK Biobank study found that poor CVH is significantly associated with lower WM microstructural integrity, as reflected by lower FA_mean_ values or higher OD_mean_ values. The study identified 10 plasma proteins with strong mediating effects, with leptin exhibiting the strongest association with OD_mean_ and FA_mean_, mainly as a PIE, followed closely by carboxypeptidase M (CPM). Most notably, poor CVH was strongly associated with OD_mean_, with over 50 plasma proteins mediating the relationship. A PCA identified three PCs, all of which were strong mediators between poor CVH and OD_mean_ at the highest AD PRS tertile.

Research has uncovered a link between dementia incidence and certain components of the LS7,^14^, ^15^, ^16^, ^69^ such as smoking, sub‐optimal physical exercise, and elevated fasting glucose.^69^ More recent studies have found a putative protective effect of CVH on brain health, with healthier LS7 profiles associated with lower volumes of WM hyperintensities and larger brain volume.^70^, ^71^ A study using a cohort from the U.S.‐based Northern Manhattan Study found that a higher number of components of the LS7 were associated with reduced WM hyperintensity volume and silent brain infarct, and greater cerebral volume.^72^

Ten proteins, namely LEP (leptin), CPM, F9, FGF21, PRSS8, LILRA5, CA14, VWA1, BAIAP2, and WFIKKN2, were found to be strong mediators between poor CVH and two DTI/NODDI metrics: FA and OD. Leptin, a hormone produced by adipose cells, has a primary role in regulating energy balance and appetite.^73^ Its link to cardiovascular disease is mainly through obesity driven by leptin resistance, but also through promoting inflammation, oxidative stress, and endothelial dysfunction.^73^ Cross‐sectional and cohort studies have shown that high leptin levels can impact cognition, dementia, and AD.^74^, ^75^, ^76^ In the Health ABC cohort study, participants in the high leptin group were 34% less likely to experience clinically significant cognitive decline over a 4‐year period.^75^ In a prospective study of 785 people without dementia, higher leptin was linked to a lower risk of incident dementia and AD.^76^ In older persons without major neurological or psychiatric disorders, serum leptin was inversely correlated with executive function.^74^ Other studies have reported that high leptin is associated with lower risk for dementia, although some studies find no association.^77^, ^78^ Table S3 and OSM 7 include a detailed literature review on the 10 plasma proteins and their association with CVH and dementia‐related traits. As expected, leptin in our study was highly correlated with the BMI component of the LE8 score, along with the total score and the biological sub‐score. This suggests that leptin's mediating effect between poor CVH and FA or OD is driven mainly by adiposity as measured by BMI.

Recent cross‐sectional data on older men aged 50 years and over, with cerebral small vessel disease, found that in men higher leptin levels were associated with lower gray matter and total brain volumes.^79^ However, in the Framingham Heart Study, higher leptin levels were associated with higher total cerebral brain volume.^76^ Therefore, some evidence is accumulating as to leptin's relationship with brain volumes, whereas little is known regarding its association with markers of brain WM microstructural integrity.

Several molecular pathways were proposed to elucidate the connection between leptin and cognition. Leptin may be involved in hippocampus synaptic plasticity, which is associated with learning and memory and is linked to leptin‐induced long‐term potentiation (LTP).^80^, ^81^ Second, it controls neuron excitability by adjusting cognitive function via the MAPK signaling pathway.^80^ Furthermore, leptin may work as an anti‐apoptotic agent in stressful conditions and provide neuroprotective effects.^82^, ^83^ Leptin was found to enhance the uptake of amyloid beta into the cell through ApoE, leading to a decrease in its accumulation outside the cell.^84^ Leptin and insulin work together to decrease the excessive phosphorylation of tau, a key element of the neurofibrillary tangle that is a key characteristic of AD.^85^, ^86^ Treating transgenic mice (AD model) with leptin led to enhancements in memory skills.^86^ It is important to note that the content of leptin in brain tissues and cerebrospinal fluid does not rise at blood leptin levels beyond 25–30 ng/mL.^87^ Because of this occurrence, leptin resistance and obesity are prone to develop.^87^ Excessive blood levels of leptin cause a decrease in blood–brain barrier permeability.^87^ A more detailed review of the literature for associations of leptin (and of the other nine top consistent mediators: CPM, F9, FGF21, PRSS8, LILRA5, CA14, VWA1, BAIAP2, and WFIKKN2) with dementia and CVH traits is provided in OSM 7 (Appendix I).

Our current study has several strengths but some limitations. First, the study incorporated a large‐scale proteomic analysis and is the first cohort study with sufficient power to analyze the plasma proteome's mediating and moderating effects in the relationship between poor CVH and indices of brain WM health. Second, LE8 as a composite measure was properly established using data from the UK Biobank, comprising dietary, pharmaceutical, and environmental tobacco exposure indicators that were easily available during the baseline assessment. Third, the UK Biobank covers numerous topic areas, allowing for accurate estimations of exposure–outcome connections by confounder adjustment. Potential study limitations include selection bias because of missing data, mainly due to the proportionate exclusion of racial minorities and lower SES groups as well as females from the final sample, and measurement inaccuracy because several characteristics were examined using self‐report. Although key variables have been controlled for, residual confounding is possible due to the study's observational design. Furthermore, although prevalent dementia cases were removed, reverse causality remains an issue for the relationship between poor CVH and subclinical stages of dementia. Furthermore, although DTI indices are sensitive for the detection of early damage to cerebral tissue microstructure, they are not unique to any underlying determinant of brain tissue, rendering precise data interpretation challenging.^88^ Although the NODDI methodology was presented to improve specificity to neurite degeneration and dispersion using ICVF and OD measurements, derived parameters suffer from experimental and modeling limitations. Indeed, the generated ICVF and ISOVF parameters have been found to be sensitive to some experimental MRI parameters and underlying assumptions such as considering constant diffusivity parameters across the brain regions and adult subjects.^88^ This may have precluded detection of CVH effect on axonal integrity as derived from the ICVF/NDI measurement. Nevertheless, the WM integrity associations observed in our work could be driven by differences in WM myelination as captured using DTI‐FA and NODD‐OD metrics, which are very sensitive to myelin content and organization. Indeed, myelin alterations, as measured using advanced and direct MRI measures of myelin content, have recently been associated with various metabolic and vascular risk factors, including obesity,^89^ physical activity,^90^ and changes in cognitions.^91^ Additional investigations, including using improved versions of NODDI^92^ and direct MRI measures of myelin content, are still required to corroborate, complement, or expand upon, our findings. It is important to note that DTI and NODDI metrics, although highly informative for WM integrity, do not capture all possible aspects of brain microstructure. Furthermore, the four‐way decomposition method includes assumptions and restrictions that are addressed elsewhere.^62^ Finally, due to limited statistical power, we did not stratify based on race/ethnicity.

In summary, our study found that suboptimal CVH was linked to poor DTI and NODDI measures of WM microstructural integrity, specifically lower FA_mean_ and higher OD_mean_, underscoring the necessity to improve CVH as a preventive strategy. Furthermore, this relationship between poor CVH and decrements in cerebral health was mediated through multiple plasma proteins, with leptin being the principal driver. Poor CVH was strongly associated with OD_mean_, with over 50 plasma proteins mediating the relationship. Because these dMRI metrics are among key biomarkers designed to reflect brain WM neurodegeneration, serum leptin can be used as a more readily available biomarker prior to using these more elaborate methods to screen for brain WM changes related to AD and related dementias. Future research should explore these mediational relationships with repeated measures on FA and OD metrics. Enhancing leptin sensitivity may be a therapeutic target to improve WM microstructural integrity, an early marker of brain health.

## AUTHOR CONTRIBUTIONS

**May A. Beydoun**: Study concept; data acquisition; plan of analysis; data management and statistical analysis; data visualization; literature search and review; write‐up of parts of the manuscript; revision of the manuscript. **Hind A. Beydoun**: Study concept; plan of analysis; assistance with data management and statistical analysis; literature search and review; write‐up of parts of the manuscript; revision of the manuscript. **Nicole Noren Hooten**: Study concept; plan of analysis; literature search and review; data visualization; write‐up of parts of the manuscript; revision of the manuscript. **Zhiguang Li**: Study concept; plan of analysis; literature search and review; data visualization; write‐up of parts of the manuscript; revision of the manuscript. **Yi‐Han Hu**: Study concept; plan of analysis; literature search and review; data visualization; write‐up of parts of the manuscript; revision of the manuscript. **Michael F. Georgescu**: Literature search and review; write‐up of parts of the manuscript; revision of the manuscript. **Sharmin Hossain**: Plan of analysis; write‐up of parts of the manuscript; literature search and review; revision of the manuscript. **Toshiko Tanaka**: Plan of analysis; literature search and review; write‐up of parts of the manuscript; revision of the manuscript. **Mustapha Bouhrara**: Plan of analysis; literature search and review; write‐up of parts of the manuscript; revision of the manuscript. **Christian A. Maino‐Vieytes**: Study concept; plan of analysis; literature search and review; data visualization; write‐up of parts of the manuscript; revision of the manuscript. **Marie T. Fanelli‐Kuczmarski**: Plan of analysis; assistance with data management; write‐up of parts of the manuscript; revision of the manuscript. **Lenore J. Launer**: Plan of analysis; literature search and review; write‐up of parts of the manuscript; revision of the manuscript. **Michele K. Evans**: Data acquisition; plan of analysis; write‐up of parts of the manuscript; revision of the manuscript. **Alan B. Zonderman**: Data acquisition; plan of analysis; data visualization; write‐up of parts of the manuscript; revision of the manuscript.

## CONFLICT OF INTEREST STATEMENT

The authors declare no conflicts of interest. Author disclosures are available in the Supporting Information.

## CONSENT STATEMENT

The studies with human participants were reviewed and approved by the UK Biobank, which has approval from various institutional review boards, including the North West Multi‐Center Research Ethics Committee for the United Kingdom, the National Information Governance Board for Health and Social Care for England and Wales, and the Community Health Index Advisory Group for Scotland. All participants provided informed permission for the study using a touch‐screen interface that necessitated acceptance to each statement on the consent form and the participant's signature on an electronic pad. Written informed permission was not necessary for this investigation, since it complied with national laws and institutional regulations.

## DIVERSITY, EQUITY, AND INCLUSION STATEMENT

This study acknowledges the significance of diversity, equity, and inclusion, which is highly relevant to Alzheimer's disease (AD) and dementia research. AD and dementias of all types disproportionately impact underrepresented groups, particularly ethnic minorities and persons of lower individual, household, and area‐level socioeconomic status, who frequently encounter obstacles to early detection, health care access, and research participation. Our objective is to rectify these inequities by advocating for inclusive research methodologies and cultivating equitable health outcomes.

We diligently endeavor to incorporate people from many racial, cultural, and socioeconomic backgrounds in our research, since their involvement is essential for comprehending the heterogeneous characteristics of AD and related dementias. Furthermore, we are dedicated to investigating social determinants of health and their influence on the advancement and results of these health conditions.

We want to enhance tailored strategies for dementia care in general that consider the distinct biological and socio‐environmental exposures encountered by various populations. We underscore the significance of community participation and collaboration with many stakeholders to enhance outreach, accessibility, and the dissemination of our findings, guaranteeing that our research serves all populations impacted by these circumstances.

## Supporting information



Supporting information

Supporting information

Supporting information

Supporting information

Supporting information

Supporting information

Supporting information

Supporting information

Supporting information

Supporting information

Supporting information

Supporting information

Supporting information

Supporting information

Supporting information
