# Supplementary material for: Plasma proteomic biomarkers as mediators or moderators for the association between poor cardiovascular health and white matter microstructural integrity: The UK Biobank study
Source: Alzheimers Dement. 2025 Jan 17;21(2):e14507. doi: 10.1002/alz.14507 (PMC11864230; doi:10.1002/alz.14507)
Supplement: Supplementary file 1 — Supporting information [file ALZ-21-e14507-s014.pdf]

APPENDIX I

ONLINE SUPPLEMENTARY MATERIALS

Plasma proteomic biomarkers as mediators or moderators for the association between poor cardiovascular health and white matter microstructural integrity: The UK Biobank study

May A. Beydoun et. al

OSM1: Diffusion-weighted imaging (DWI) acquisition and processing for dMRI phenotypes

Main source: http://biobank.ctsu.ox.ac.uk/crystal/docs/brain_mri.pdf)

Other sources : [1-3]

A spin-echo echo-planar MRI sequence was used to acquire the diffusion magnetic resonance imaging (dMRI), yielding 10 T2-weighted volumes at b = 0 s/mm^2, as well as diffusion-weighted images at b = 1000 s/mm^2 and b = 2000 s/mm^2 each obtained with 50 distinct diffusion-encoding directions. Repetition time (TR) of 3600 ms, echo time (TE) of 92 ms, multiband acceleration factor of 3All images were acquired with an isotropic voxel size of 2 mm . Details of the dMRI acquisition is provided by Gossai and colleagues (2016) [4]. Images were processed using the FSL software for head motion, eddy current distortion, and outlier slice corrections [5-7].

DTI analysis: Using the FSL's DTIFIT tool, the preprocessed DW images (b = 0 s/mm^2 and b = 1,000 s/mm^2) were utilized to create corresponding fractional anisotropy (FA) and mean diffusivity (MD) maps [5, 8]. While MD reflects the average magnitude of molecular displacement by diffusion with higher values indicate more freely diffusing water, FA reflects the directionality of molecular displacement by diffusion with values between 0 and 1; higher values are an indicator of anisotropic diffusion that confers a preferred direction. Reduced FA or increased MD in WM may indicate axon or myelin sheath degradation. Only the white matter skeleton tract was included in the FA and MD measurements. Parameter maps of FA were registered to the JHU white matter Atlas (ICBM-DTI-81) to define the white matter skeleton [5, 9]. Tract-Based Spatial statistics (TBSS)-derived measures were then computed by averaging the skeletonized image of each FA map across subjects within a set of 48 standard-space tract masks, using FSL's TBSS [5, 10].

NODDI analysis: Using the AMICO (Accelerated Microstructure Imaging via Convex Optimization) tool (https://github.com/daducci/AMICO), dMRI data was processed in addition to the DTI fitting. This was done in accordance with prior studies [11, 12]. The NODDI measures the orientation dispersion index (ODI), which is a measure of axonal disorganization; the intra-cellular volume fraction (ICVF), which is an index of white matter axonal density; and the isotropic water volume fraction (ISOVF), which is a measure of the freely-moving water that is typically attributed to CSF. Here again, the resultant NODDI indice maps were registered to the JHU atlas, with ICVF, ISOVF, and ODI values computed in 48 standard-space tract regions.

Supplementary Table 1 (A). Detailed list of DTI metrics, tract-specific

		List			WM	FA	Y	Y
**Tract-specific FA**								
Mean FA in anterior corona radiata on FA skeleton (left)	Yes	25079	FA_ACR_Left	Selected tracts	WM	FA	Y	Y
Mean FA in anterior corona radiata on FA skeleton (right)	Yes	25078	FA_ACR_Right	Selected tracts	WM	FA	Y	Y
Mean FA in anterior limb of internal capsule on FA skeleton (left)	Yes	25073	FA_ALIC_Left	Selected tracts	WM	FA	Y	Y
Mean FA in anterior limb of internal capsule on FA skeleton (right)	Yes	25072	FA_ALIC_Right	Selected tracts	WM	FA	Y	Y
Mean FA in body of corpus callosum on FA skeleton	No	25059	FA_BCC	Selected tracts	WM	FA	Y	Y
Mean FA in cerebral peduncle on FA skeleton (left)	Yes	25071	FA_CP_Left	Selected tracts	WM	FA	Y	Y
Mean FA in cerebral peduncle on FA skeleton (right)	Yes	25070	FA_CP_Right	Selected tracts	WM	FA	Y	Y
Mean FA in cingulum cingulate gyrus on FA skeleton (left)	Yes	25091	FA_CCG_Left	Selected tracts	WM	FA	Y	Y
Mean FA in cingulum cingulate gyrus on FA skeleton (right)	Yes	25090	FA_CCG_Right	Selected tracts	WM	FA	Y	Y
Mean FA in cingulum	Yes	25093	FA_CH_Left	Selected tracts	WM	FA	Y	Y

hippocampus on FA skeleton (left)								
Mean FA in cingulum hippocampus on FA skeleton (right)	Yes	25092	FA_CH_Right	Selected tracts	WM	FA	Y	Y
Mean FA in corticospinal tract on FA skeleton (left)	Yes	25063	FA_CT_Left	Selected tracts	WM	FA	Y	Y
Mean FA in corticospinal tract on FA skeleton (right)	Yes	25062	FA_CT_Right	Selected tracts	WM	FA	Y	Y
Mean FA in external capsule on FA skeleton (left)	Yes	25089	FA_EC_Left	Selected tracts	WM	FA	Y	Y
Mean FA in external capsule on FA skeleton (right)	Yes	25088	FA_EC_Right	Selected tracts	WM	FA	Y	Y
Mean FA in fornix cres+stria terminalis on FA skeleton (left)	Yes	25095	FA_FCST_Left	Selected tracts	WM	FA	Y	Y
Mean FA in fornix cres+stria terminalis on FA skeleton (right)	Yes	25094	FA_FCST_Right	Selected tracts	WM	FA	Y	Y
Mean FA in fornix on FA skeleton	No	25061	FA_FO	Selected tracts	WM	FA	Y	Y
Mean FA in genu of corpus callosum on FA skeleton	No	25058	FA_GCC	Selected tracts	WM	FA	Y	Y
Mean FA in inferior cerebellar peduncle on FA skeleton (left)	Yes	25067	FA_ICP_Left	Selected tracts	WM	FA	Y	Y
Mean FA in inferior cerebellar peduncle	Yes	25066	FA_ICP_Right	Selected tracts	WM	FA	Y	Y

on FA skeleton (right)								
Mean FA in medial lemniscus on FA skeleton (left)	Yes	25065	FA_ML_Left	Selected tracts	WM	FA	Y	Y
Mean FA in medial lemniscus on FA skeleton (right)	Yes	25064	FA_ML_Right	Selected tracts	WM	FA	Y	Y
Mean FA in middle cerebellar peduncle on FA skeleton	No	25056	FA_MCP	Selected tracts	WM	FA	Y	Y
Mean FA in pontine crossing tract on FA skeleton	No	25057	FA_PC	Selected tracts	WM	FA	Y	Y
Mean FA in posterior corona radiata on FA skeleton (left)	Yes	25083	FA_PCR_Left	Selected tracts	WM	FA	Y	Y
Mean FA in posterior corona radiata on FA skeleton (right)	Yes	25082	FA_PCR_Left	Selected tracts	WM	FA	Y	Y
Mean FA in posterior limb of internal capsule on FA skeleton (left)	Yes	25075	FA_PLIC_Left	Selected tracts	WM	FA	Y	Y
Mean FA in posterior limb of internal capsule on FA skeleton (right)	Yes	25074	FA_PLIC_Right	Selected tracts	WM	FA	Y	Y
Mean FA in posterior thalamic radiation on FA skeleton (left)	Yes	25085	FA_PTR_Left	Selected tracts	WM	FA	Y	Y
Mean FA in posterior thalamic radiation on FA skeleton (right)	Yes	25084	FA_PTR_Right	Selected tracts	WM	FA	Y	Y
Mean FA in retrolenticular part	Yes	25077	FA_RPIC_Left	Selected tracts	WM	FA	Y	Y

of internal capsule on FA skeleton (left)								
Mean FA in retrolenticular part of internal capsule on FA skeleton (right)	Yes	25076	FA_RPIC_Right	Selected tracts	WM	FA	Y	Y
Mean FA in sagittal stratum on FA skeleton (left)	Yes	25087	FA_SS_Left	Selected tracts	WM	FA	Y	Y
Mean FA in sagittal stratum on FA skeleton (right)	Yes	25086	FA_SS_Right	Selected tracts	WM	FA	Y	Y
Mean FA in splenium of corpus callosum on FA skeleton	No	25060	FA_SCC	Selected tracts	WM	FA	Y	Y
Mean FA in superior cerebellar peduncle on FA skeleton (left)	Yes	25069	FA_SCP_Left	Selected tracts	WM	FA	Y	Y
Mean FA in superior cerebellar peduncle on FA skeleton (right)	Yes	25068	FA_SCP_Right	Selected tracts	WM	FA	Y	Y
Mean FA in superior corona radiata on FA skeleton (left)	Yes	25081	FA_SCR_Left	Selected tracts	WM	FA	Y	Y
Mean FA in superior corona radiata on FA skeleton (right)	Yes	25080	FA_SCR_Right	Selected tracts	WM	FA	Y	Y
Mean FA in superior fronto-occipital fasciculus on FA skeleton (left)	Yes	25099	FA_FOF_Left	Selected tracts	WM	FA	Y	Y

Mean FA in superior fronto-occipital fasciculus on FA skeleton (right)	Yes	25098	FA_FOF_Right	Selected tracts	WM	FA	Y	Y
Mean FA in superior longitudinal fasciculus on FA skeleton (left)	Yes	25097	FA_SLF_Left	Selected tracts	WM	FA	Y	Y
Mean FA in superior longitudinal fasciculus on FA skeleton (right)	Yes	25096	FA_SLF_Right	Selected tracts	WM	FA	Y	Y
Mean FA in tapetum on FA skeleton (left)	Yes	25103	FA_TP_Left	Selected tracts	WM	FA	Y	Y
Mean FA in tapetum on FA skeleton (right)	Yes	25102	FA_TP_Right	Selected tracts	WM	FA	Y	Y
Mean FA in uncinate fasciculus on FA skeleton (left)	Yes	25101	FA_UNC_Left	Selected tracts	WM	FA	Y	Y
Mean FA in uncinate fasciculus on FA skeleton (right)	Yes	25100	FA_UNC_Right	Selected tracts	WM	FA	Y	Y
**Tract-specific MD**		**List**		**Selected tracts**	**WM**	**MD**	**Y**	**Y**
Mean MD in anterior corona radiata on FA skeleton (left)	Yes	25127	MD_ACR_Left	**Selected tracts**	**WM**	**MD**	**Y**	**Y**
Mean MD in anterior corona radiata on FA skeleton (right)	Yes	25126	MD_ACR_Right	**Selected tracts**	**WM**	**MD**	**Y**	**Y**

Mean MD in anterior limb of internal capsule on FA skeleton (left)	Yes	25121	MD_ALIC_Left	**Selected tracts**	**WM**	**MD**	**Y**	**Y**
Mean MD in anterior limb of internal capsule on FA skeleton (right)	Yes	25120	MD_ALIC_Right	**Selected tracts**	**WM**	**MD**	**Y**	**Y**
Mean MD in body of corpus callosum on FA skeleton	No	25107	MD_BCC	**Selected tracts**	**WM**	**MD**	**Y**	**Y**
Mean MD in cerebral peduncle on FA skeleton (left)	Yes	25119	MD_CP_Left	**Selected tracts**	**WM**	**MD**	**Y**	**Y**
Mean MD in cerebral peduncle on FA skeleton (right)	Yes	25118	MD_CP_Right	**Selected tracts**	**WM**	**MD**	**Y**	**Y**
Mean MD in cingulum cingulate gyrus on FA skeleton (left)	Yes	25139	MD_CCG_Left	**Selected tracts**	**WM**	**MD**	**Y**	**Y**
Mean MD in cingulum cingulate gyrus on FA skeleton (right)	Yes	25138	MD_CCG_Right	**Selected tracts**	**WM**	**MD**	**Y**	**Y**
Mean MD in cingulum hippocampus on FA skeleton (left)	Yes	25141	MD_CH_Left	**Selected tracts**	**WM**	**MD**	**Y**	**Y**
Mean MD in cingulum hippocampus on FA skeleton (right)	Yes	25140	MD_CH_Right	**Selected tracts**	**WM**	**MD**	**Y**	**Y**
Mean MD in corticospinal tract on FA skeleton (left)	Yes	25111	MD_CT_Left	**Selected tracts**	**WM**	**MD**	**Y**	**Y**

Mean MD in corticospinal tract on FA skeleton (right)	Yes	25110	MD_CT_Right	**Selected tracts**	**WM**	**MD**	**Y**	**Y**
Mean MD in external capsule on FA skeleton (left)	Yes	25137	MD_EC_Left	**Selected tracts**	**WM**	**MD**	**Y**	**Y**
Mean MD in external capsule on FA skeleton (right)	Yes	25136	MD_EC_Right	**Selected tracts**	**WM**	**MD**	**Y**	**Y**
Mean MD in fornix cres+stria terminalis on FA skeleton (left)	Yes	25143	MD_FCST_Left	**Selected tracts**	**WM**	**MD**	**Y**	**Y**
Mean MD in fornix cres+stria terminalis on FA skeleton (right)	Yes	25142	MD_FCST_Right	**Selected tracts**	**WM**	**MD**	**Y**	**Y**
Mean MD in fornix on FA skeleton	No	25109	MD_FO	**Selected tracts**	**WM**	**MD**	**Y**	**Y**
Mean MD in genu of corpus callosum on FA skeleton	No	25106	MD_GCC	**Selected tracts**	**WM**	**MD**	**Y**	**Y**
Mean MD in inferior cerebellar peduncle on FA skeleton (left)	Yes	25115	MD_ICP_Left	**Selected tracts**	**WM**	**MD**	**Y**	**Y**
Mean MD in inferior cerebellar peduncle on FA skeleton (right)	Yes	25114	MD_ICP_Right	**Selected tracts**	**WM**	**MD**	**Y**	**Y**
Mean MD in medial lemniscus on FA skeleton (left)	Yes	25113	MD_ML_Left	**Selected tracts**	**WM**	**MD**	**Y**	**Y**
Mean MD in medial lemniscus on FA skeleton (right)	Yes	25112	MD_ML_Right	**Selected tracts**	**WM**	**MD**	**Y**	**Y**
Mean MD in middle cerebellar peduncle on FA skeleton	No	25104	MD_MCP	**Selected tracts**	**WM**	**MD**	**Y**	**Y**

Mean MD in pontine crossing tract on FA skeleton	No	25105	Mean_PC	**Selected tracts**	**WM**	**MD**	**Y**	**Y**
Mean MD in posterior corona radiata on FA skeleton (left)	Yes	25131	MD_PCR_Left	**Selected tracts**	**WM**	**MD**	**Y**	**Y**
Mean MD in posterior corona radiata on FA skeleton (right)	Yes	25130	MD_PCR_Left	**Selected tracts**	**WM**	**MD**	**Y**	**Y**
Mean MD in posterior limb of internal capsule on FA skeleton (left)	Yes	25123	MD_PLIC_Left	**Selected tracts**	**WM**	**MD**	**Y**	**Y**
Mean MD in posterior limb of internal capsule on FA skeleton (right)	Yes	25122	MD_PLIC_Right	**Selected tracts**	**WM**	**MD**	**Y**	**Y**
Mean MD in posterior thalamic radiation on FA skeleton (left)	Yes	25133	MD_PTR_Left	**Selected tracts**	**WM**	**MD**	**Y**	**Y**
Mean MD in posterior thalamic radiation on FA skeleton (right)	Yes	25132	MD_PTR_Right	**Selected tracts**	**WM**	**MD**	**Y**	**Y**
Mean MD in retrolenticular part of internal capsule on FA skeleton (left)	Yes	25125	MD_RPIC_Left	**Selected tracts**	**WM**	**MD**	**Y**	**Y**
Mean MD in retrolenticular part of internal capsule on FA skeleton (right)	Yes	25124	MD_RPIC_Right	**Selected tracts**	**WM**	**MD**	**Y**	**Y**
Mean MD in sagittal stratum on FA skeleton (left)	Yes	25135	MD_SS_Left	**Selected tracts**	**WM**	**MD**	**Y**	**Y**

Mean MD in sagittal stratum on FA skeleton (right)	Yes	25134	MD_SS_Right	**Selected tracts**	**WM**	**MD**	**Y**	**Y**
Mean MD in splenium of corpus callosum on FA skeleton	No	25108	MD_SCC	**Selected tracts**	**WM**	**MD**	**Y**	**Y**
Mean MD in superior cerebellar peduncle on FA skeleton (left)	Yes	25117	MD_SCP_Left	**Selected tracts**	**WM**	**MD**	**Y**	**Y**
Mean MD in superior cerebellar peduncle on FA skeleton (right)	Yes	25116	MD_SCP_Right	**Selected tracts**	**WM**	**MD**	**Y**	**Y**
Mean MD in superior corona radiata on FA skeleton (left)	Yes	25129	MD_SCR_Left	**Selected tracts**	**WM**	**MD**	**Y**	**Y**
Mean MD in superior corona radiata on FA skeleton (right)	Yes	25128	MD_SCR_Right	**Selected tracts**	**WM**	**MD**	**Y**	**Y**
Mean MD in superior fronto-occipital fasciculus on FA skeleton (left)	Yes	25147	MD_FOF_Left	**Selected tracts**	**WM**	**MD**	**Y**	**Y**
Mean MD in superior fronto-occipital fasciculus on FA skeleton (right)	Yes	25146	MD_FOF_Right	**Selected tracts**	**WM**	**MD**	**Y**	**Y**
Mean MD in superior longitudinal fasciculus on FA skeleton (left)	Yes	25145	MD_SLF_Left	**Selected tracts**	**WM**	**MD**	**Y**	**Y**
Mean MD in superior	Yes	25144	MD_SLF_Right	**Selected tracts**	**WM**	**MD**	**Y**	**Y**

longitudinal fasciculus on FA skeleton (right)								
Mean MD in tapetum on FA skeleton (left)	Yes	25151	MD_TP_Left	**Selected tracts**	**WM**	**MD**	**Y**	**Y**
Mean MD in tapetum on FA skeleton (right)	Yes	25150	MD_TP_Right	**Selected tracts**	**WM**	**MD**	**Y**	**Y**
Mean MD in uncinate fasciculus on FA skeleton (left)	Yes	25149	MD_UNC_Left	**Selected tracts**	**WM**	**MD**	**Y**	**Y**
Mean MD in uncinate fasciculus on FA skeleton (right)	Yes	25148	MD_UNC_Right	**Selected tracts**	**WM**	**MD**	**Y**	**Y**

Sources: [13-15]

Supplementary Table 1 (B) Detailed list of tract-specific NODDI metrics

				dMRI					
**Measure**	**Left/Right (Yes/No)**	**UKB field**		**ROI abbrev.**	**Region**	**GM/WM**	**ISOVF, ICVF, OD**	**Tract**	**Sub-cortical (y/n)**
**Tract-specific ISOVF**						**WM**	ISOVF	**Y**	**Y**
Mean ISOVF in anterior corona radiata on FA skeleton (left)	Yes	25463		ISOVF_ACR_Left	Selected tracts	WM	ISOVF	Y	Y
Mean ISOVF in anterior corona radiata on FA skeleton (right)	Yes	25462		ISOVF_ACR_Right	Selected tracts	WM	ISOVF	Y	Y
Mean ISOVF in anterior limb of internal capsule on FA skeleton (left)	Yes	25457		ISOVF_ALIC_Left	Selected tracts	WM	ISOVF	Y	Y
Mean ISOVF in anterior limb of internal capsule on FA skeleton (right)	Yes	25456		ISOVF_ALIC_Right	Selected tracts	WM	ISOVF	Y	Y
Mean ISOVF in body of corpus callosum on FA skeleton	No	25443		ISOVF_BCC	Selected tracts	WM	ISOVF	Y	Y
Mean ISOVF in cerebral peduncle on FA skeleton (left)	Yes	25455		ISOVF_CP_Left	Selected tracts	WM	ISOVF	Y	Y
Mean ISOVF in cerebral peduncle on FA skeleton (right)	Yes	25454		ISOVF_CP_Right	Selected tracts	WM	ISOVF	Y	Y
Mean ISOVF in cingulum cingulate gyrus on FA skeleton (left)	Yes	25475		ISOVF_CCG_Left	Selected tracts	WM	ISOVF	Y	Y
Mean ISOVF in cingulum cingulate gyrus on FA skeleton (right)	Yes	25474		ISOVF_CCG_Right	Selected tracts	WM	ISOVF	Y	Y
Mean ISOVF in cingulum hippocampus on FA skeleton (left)	Yes	25477		ISOVF_CH_Left	Selected tracts	WM	ISOVF	Y	Y
Mean ISOVF in cingulum hippocampus on FA skeleton (right)	Yes	25476		ISOVF_CH_Right	Selected tracts	WM	ISOVF	Y	Y
Mean ISOVF in corticospinal tract on FA skeleton (left)	Yes	25447		ISOVF_CT_Left	Selected tracts	WM	ISOVF	Y	Y
Mean ISOVF in corticospinal tract on FA skeleton (right)	Yes	25446		ISOVF_CT_Right	Selected tracts	WM	ISOVF	Y	Y
Mean ISOVF in external capsule on FA skeleton (left)	Yes	25473		ISOVF_EC_Left	Selected tracts	WM	ISOVF	Y	Y

Mean ISOVF in external capsule on FA skeleton (right)	Yes	25472		ISOVF_EC_Right	Selected tracts	WM	ISOVF	Y	Y
Mean ISOVF in fornix cres+stria terminalis on FA skeleton (left)	Yes	25479		ISOVF_FCST_Left	Selected tracts	WM	ISOVF	Y	Y
Mean ISOVF in fornix cres+stria terminalis on FA skeleton (right)	Yes	25478		ISOVF_FCST_Right	Selected tracts	WM	ISOVF	Y	Y
Mean ISOVF in fornix on FA skeleton	No	25445		ISOVF_FO	Selected tracts	WM	ISOVF	Y	Y
Mean ISOVF in genu of corpus callosum on FA skeleton	No	25442		ISOVF_GCC	Selected tracts	WM	ISOVF	Y	Y
Mean ISOVF in inferior cerebellar peduncle on FA skeleton (left)	Yes	25451		ISOVF_ICP_Left	Selected tracts	WM	ISOVF	Y	Y
Mean ISOVF in inferior cerebellar peduncle on FA skeleton (right)	Yes	25450		ISOVF_ICP_Right	Selected tracts	WM	ISOVF	Y	Y
Mean ISOVF in medial lemniscus on FA skeleton (left)	Yes	25449		ISOVF_ML_Left	Selected tracts	WM	ISOVF	Y	Y
Mean ISOVF in medial lemniscus on FA skeleton (right)	Yes	25448		ISOVF_ML_Right	Selected tracts	WM	ISOVF	Y	Y
Mean ISOVF in middle cerebellar peduncle on FA skeleton	No	25440		ISOVF_MCP	Selected tracts	WM	ISOVF	Y	Y
Mean ISOVF in pontine crossing tract on FA skeleton	No	25441		ISOVF_PCT	Selected tracts	WM	ISOVF	Y	Y
Mean ISOVF in posterior corona radiata on FA skeleton (left)	Yes	25467		ISOVF_PCR_Left	Selected tracts	WM	ISOVF	Y	Y
Mean ISOVF in posterior corona radiata on FA skeleton (right)	Yes	25466		ISOVF_PCR_Left	Selected tracts	WM	ISOVF	Y	Y
Mean ISOVF in posterior limb of internal capsule on FA skeleton (left)	Yes	25459		ISOVF_PLIC_Left	Selected tracts	WM	ISOVF	Y	Y
Mean ISOVF in posterior limb of internal capsule on FA skeleton (right)	Yes	25458		ISOVF_PLIC_Right	Selected tracts	WM	ISOVF	Y	Y
Mean ISOVF in posterior thalamic radiation on FA skeleton (left)	Yes	25469		ISOVF_PTR_Left	Selected tracts	WM	ISOVF	Y	Y
Mean ISOVF in posterior thalamic radiation on FA skeleton (right)	Yes	25468		ISOVF_PTR_Right	Selected tracts	WM	ISOVF	Y	Y
Mean ISOVF in retrolenticular part of internal capsule on FA skeleton (left)	Yes	25461		ISOVF_RPIC_Left	Selected tracts	WM	ISOVF	Y	Y
Mean ISOVF in retrolenticular part of internal capsule on FA skeleton (right)	Yes	25460		ISOVF_RPIC_Right	Selected tracts	WM	ISOVF	Y	Y
Mean ISOVF in sagittal stratum on FA skeleton (left)	Yes	25471		ISOVF_SS_Left	Selected tracts	WM	ISOVF	Y	Y

Mean ISOVF in sagittal stratum on FA skeleton (right)	Yes	25470		ISOVF_SS_Right	Selected tracts	WM	ISOVF	Y	Y
Mean ISOVF in splenium of corpus callosum on FA skeleton	No	25444		ISOVF_SCC	Selected tracts	WM	ISOVF	Y	Y
Mean ISOVF in superior cerebellar peduncle on FA skeleton (left)	Yes	25453		ISOVF_SCP_Left	Selected tracts	WM	ISOVF	Y	Y
Mean ISOVF in superior cerebellar peduncle on FA skeleton (right)	Yes	25452		ISOVF_SCP_Right	Selected tracts	WM	ISOVF	Y	Y
Mean ISOVF in superior corona radiata on FA skeleton (left)	Yes	25465		ISOVF_SCR_Left	Selected tracts	WM	ISOVF	Y	Y
Mean ISOVF in superior corona radiata on FA skeleton (right)	Yes	25464		ISOVF_SCR_Right	Selected tracts	WM	ISOVF	Y	Y
Mean ISOVF in superior fronto-occipital fasciculus on FA skeleton (left)	Yes	25483		ISOVF_FOF_Left	Selected tracts	WM	ISOVF	Y	Y
Mean ISOVF in superior fronto-occipital fasciculus on FA skeleton (right)	Yes	25482		ISOVF_FOF_Right	Selected tracts	WM	ISOVF	Y	Y
Mean ISOVF in superior longitudinal fasciculus on FA skeleton (left)	Yes	25481		ISOVF_SLF_Left	Selected tracts	WM	ISOVF	Y	Y
Mean ISOVF in superior longitudinal fasciculus on FA skeleton (right)	Yes	25480		ISOVF_SLF_Right	Selected tracts	WM	ISOVF	Y	Y
Mean ISOVF in tapetum on FA skeleton (left)	Yes	25487		ISOVF_TP_Left	Selected tracts	WM	ISOVF	Y	Y
Mean ISOVF in tapetum on FA skeleton (right)	Yes	25486		ISOVF_TP_Right	Selected tracts	WM	ISOVF	Y	Y
Mean ISOVF in uncinate fasciculus on FA skeleton (left)	Yes	25485		ISOVF_UNC_Left	Selected tracts	WM	ISOVF	Y	Y
Mean ISOVF in uncinate fasciculus on FA skeleton (right)	Yes	25484		ISOVF_UNC_Right	Selected tracts	WM	ISOVF	Y	Y
**Tract-specific ICVF**		**List**			**Selected tracts**	**WM**	**ICVF**	**Y**	**Y**
Mean ICVF in anterior corona radiata on FA skeleton (left)	Yes	25367		ICVF_ACR_Left	**Selected tracts**	**WM**	**ICVF**	**Y**	**Y**
Mean ICVF in anterior corona radiata on FA skeleton (right)	Yes	25366		ICVF_ACR_Right	**Selected tracts**	**WM**	**ICVF**	**Y**	**Y**
Mean ICVF in anterior limb of internal capsule on FA skeleton (left)	Yes	25361		ICVF_ALIC_Left	**Selected tracts**	**WM**	**ICVF**	**Y**	**Y**
Mean ICVF in anterior limb of internal capsule on FA skeleton (right)	Yes	25360		ICVF_ALIC_Right	**Selected tracts**	**WM**	**ICVF**	**Y**	**Y**
Mean ICVF in body of corpus callosum on FA skeleton	No	25347		ICVF_BCC	**Selected tracts**	**WM**	**ICVF**	**Y**	**Y**

Mean ICVF in cerebral peduncle on FA skeleton (left)	Yes	25359		ICVF_CP_Left	**Selected tracts**	**WM**	**ICVF**	**Y**	**Y**
Mean ICVF in cerebral peduncle on FA skeleton (right)	Yes	25358		ICVF_CP_Right	**Selected tracts**	**WM**	**ICVF**	**Y**	**Y**
Mean ICVF in cingulum cingulate gyrus on FA skeleton (left)	Yes	25379		ICVF_CCG_Left	**Selected tracts**	**WM**	**ICVF**	**Y**	**Y**
Mean ICVF in cingulum cingulate gyrus on FA skeleton (right)	Yes	25378		ICVF_CCG_Right	**Selected tracts**	**WM**	**ICVF**	**Y**	**Y**
Mean ICVF in cingulum hippocampus on FA skeleton (left)	Yes	25381		ICVF_CH_Left	**Selected tracts**	**WM**	**ICVF**	**Y**	**Y**
Mean ICVF in cingulum hippocampus on FA skeleton (right)	Yes	25380		ICVF_CH_Right	**Selected tracts**	**WM**	**ICVF**	**Y**	**Y**
Mean ICVF in corticospinal tract on FA skeleton (left)	Yes	25351		ICVF_CT_Left	**Selected tracts**	**WM**	**ICVF**	**Y**	**Y**
Mean ICVF in corticospinal tract on FA skeleton (right)	Yes	25350		ICVF_CT_Right	**Selected tracts**	**WM**	**ICVF**	**Y**	**Y**
Mean ICVF in external capsule on FA skeleton (left)	Yes	25377		ICVF_EC_Left	**Selected tracts**	**WM**	**ICVF**	**Y**	**Y**
Mean ICVF in external capsule on FA skeleton (right)	Yes	25376		ICVF_EC_Right	**Selected tracts**	**WM**	**ICVF**	**Y**	**Y**
Mean ICVF in fornix cres+stria terminalis on FA skeleton (left)	Yes	25383		ICVF_FCST_Left	**Selected tracts**	**WM**	**ICVF**	**Y**	**Y**
Mean ICVF in fornix cres+stria terminalis on FA skeleton (right)	Yes	25382		ICVF_FCST_Right	**Selected tracts**	**WM**	**ICVF**	**Y**	**Y**
Mean ICVF in fornix on FA skeleton	No	25349		ICVF_FO	**Selected tracts**	**WM**	**ICVF**	**Y**	**Y**
Mean ICVF in genu of corpus callosum on FA skeleton	No	25346		ICVF_GCC	**Selected tracts**	**WM**	**ICVF**	**Y**	**Y**
Mean ICVF in inferior cerebellar peduncle on FA skeleton (left)	Yes	25355		ICVF_ICP_Left	Selected tracts	WM	**ICVF**	Y	Y
Mean ICVF in inferior cerebellar peduncle on FA skeleton (right)	Yes	25354		ICVF_ICP_Right	Selected tracts	WM	**ICVF**	Y	Y
Mean ICVF in medial lemniscus on FA skeleton (left)	Yes	25353		ICVF_ML_Left	**Selected tracts**	**WM**	**ICVF**	**Y**	**Y**
Mean ICVF in medial lemniscus on FA skeleton (right)	Yes	25352		ICVF_ML_Right	**Selected tracts**	**WM**	**ICVF**	**Y**	**Y**
Mean ICVF in middle cerebellar peduncle on FA skeleton	No	25344		ICVF_MCP	**Selected tracts**	**WM**	**ICVF**	**Y**	**Y**
Mean ICVF in pontine crossing tract on FA skeleton	No	25345		ICVF_PCT	**Selected tracts**	**WM**	**ICVF**	**Y**	**Y**

Mean ICVF in posterior corona radiata on FA skeleton (left)	Yes	25371		ICVF_PCR_Left	**Selected tracts**	**WM**	**ICVF**	**Y**	**Y**
Mean ICVF in posterior corona radiata on FA skeleton (right)	Yes	25370		ICVF_PCR_Left	**Selected tracts**	**WM**	**ICVF**	**Y**	**Y**
Mean ICVF in posterior limb of internal capsule on FA skeleton (left)	Yes	25363		ICVF_PLIC_Left	**Selected tracts**	**WM**	**ICVF**	**Y**	**Y**
Mean ICVF in posterior limb of internal capsule on FA skeleton (right)	Yes	25362		ICVF_PLIC_Right	**Selected tracts**	**WM**	**ICVF**	**Y**	**Y**
Mean ICVF in posterior thalamic radiation on FA skeleton (left)	Yes	25373		ICVF_PTR_Left	**Selected tracts**	**WM**	**ICVF**	**Y**	**Y**
Mean ICVF in posterior thalamic radiation on FA skeleton (right)	Yes	25372		ICVF_PTR_Right	**Selected tracts**	**WM**	**ICVF**	**Y**	**Y**
Mean ICVF in retrolenticular part of internal capsule on FA skeleton (left)	Yes	25365		ICVF_RPIC_Left	**Selected tracts**	**WM**	**ICVF**	**Y**	**Y**
Mean ICVF in retrolenticular part of internal capsule on FA skeleton (right)	Yes	25364		ICVF_RPIC_Right	**Selected tracts**	**WM**	**ICVF**	**Y**	**Y**
Mean ICVF in sagittal stratum on FA skeleton (left)	Yes	25375		ICVF_SS_Left	**Selected tracts**	**WM**	**ICVF**	**Y**	**Y**
Mean ICVF in sagittal stratum on FA skeleton (right)	Yes	25374		ICVF_SS_Right	**Selected tracts**	**WM**	**ICVF**	**Y**	**Y**
Mean ICVF in splenium of corpus callosum on FA skeleton	No	25348		ICVF_SCC	**Selected tracts**	**WM**	**ICVF**	**Y**	**Y**
Mean ICVF in superior cerebellar peduncle on FA skeleton (left)	Yes	25357		ICVF_SCP_Left	**Selected tracts**	**WM**	**ICVF**	**Y**	**Y**
Mean ICVF in superior cerebellar peduncle on FA skeleton (right)	Yes	25356		ICVF_SCP_Right	**Selected tracts**	**WM**	**ICVF**	**Y**	**Y**
Mean ICVF in superior corona radiata on FA skeleton (left)	Yes	25369		ICVF_SCR_Left	**Selected tracts**	**WM**	**ICVF**	**Y**	**Y**
Mean ICVF in superior corona radiata on FA skeleton (right)	Yes	25368		ICVF_SCR_Right	**Selected tracts**	**WM**	**ICVF**	**Y**	**Y**
Mean ICVF in superior fronto-occipital fasciculus on FA skeleton (left)	Yes	25387		ICVF_FOF_Left	**Selected tracts**	**WM**	**ICVF**	**Y**	**Y**
Mean ICVF in superior fronto-occipital fasciculus on FA skeleton (right)	Yes	25386		ICVF_FOF_Right	**Selected tracts**	**WM**	**ICVF**	**Y**	**Y**
Mean ICVF in superior longitudinal fasciculus on FA skeleton (left)	Yes	25385		ICVF_SLF_Left	**Selected tracts**	**WM**	**ICVF**	**Y**	**Y**
Mean ICVF in superior longitudinal fasciculus on FA skeleton (right)	Yes	25384		ICVF_SLF_Right	**Selected tracts**	**WM**	**ICVF**	**Y**	**Y**
Mean ICVF in tapetum on FA skeleton (left)	Yes	25391		ICVF_TP_Left	**Selected tracts**	**WM**	**ICVF**	**Y**	**Y**

Mean ICVF in tapetum on FA skeleton (right)	Yes	25390		ICVF_TP_Right	**Selected tracts**	**WM**	**ICVF**	**Y**	**Y**
Mean ICVF in uncinate fasciculus on FA skeleton (left)	Yes	25389		ICVF_UNC_Left	**Selected tracts**	**WM**	**ICVF**	**Y**	**Y**
Mean ICVF in uncinate fasciculus on FA skeleton (right)	Yes	25388		ICVF_UNC_Right	**Selected tracts**	**WM**	**ICVF**	**Y**	**Y**
**Tract-specific OD**		**List**			**Selected tracts**	**WM**	**OD**	**Y**	**Y**
Mean OD in anterior corona radiata on FA skeleton (left)	Yes	25415		OD_ACR_Left	Selected tracts	WM	OD	Y	Y
Mean OD in anterior corona radiata on FA skeleton (right)	Yes	25414		OD_ACR_Right	Selected tracts	WM	OD	Y	Y
Mean OD in anterior limb of internal capsule on FA skeleton (left)	Yes	25409		OD_ALIC_Left	Selected tracts	WM	OD	Y	Y
Mean OD in anterior limb of internal capsule on FA skeleton (right)	Yes	25408		OD_ALIC_Right	Selected tracts	WM	OD	Y	Y
Mean OD in body of corpus callosum on FA skeleton	No	25395		OD_BCC	Selected tracts	WM	OD	Y	Y
Mean OD in cerebral peduncle on FA skeleton (left)	Yes	25407		OD_CP_Left	Selected tracts	WM	OD	Y	Y
Mean OD in cerebral peduncle on FA skeleton (right)	Yes	25406		OD_CP_Right	Selected tracts	WM	OD	Y	Y
Mean OD in cingulum cingulate gyrus on FA skeleton (left)	Yes	25427		OD_CCG_Left	Selected tracts	WM	OD	Y	Y
Mean OD in cingulum cingulate gyrus on FA skeleton (right)	Yes	25426		OD_CCG_Right	Selected tracts	WM	OD	Y	Y
Mean OD in cingulum hippocampus on FA skeleton (left)	Yes	25429		OD_CH_Left	Selected tracts	WM	OD	Y	Y
Mean OD in cingulum hippocampus on FA skeleton (right)	Yes	25428		OD_CH_Right	Selected tracts	WM	OD	Y	Y
Mean OD in corticospinal tract on FA skeleton (left)	Yes	25399		OD_CT_Left	Selected tracts	WM	OD	Y	Y
Mean OD in corticospinal tract on FA skeleton (right)	Yes	25398		OD_CT_Right	Selected tracts	WM	OD	Y	Y
Mean OD in external capsule on FA skeleton (left)	Yes	25425		OD_EC_Left	Selected tracts	WM	OD	Y	Y
Mean OD in external capsule on FA skeleton (right)	Yes	25424		OD_EC_Right	Selected tracts	WM	OD	Y	Y
Mean OD in fornix cres+stria terminalis on FA skeleton (left)	Yes	25431		OD_FCST_Left	Selected tracts	WM	OD	Y	Y

Mean OD in fornix cres+stria terminalis on FA skeleton (right)	Yes	25430		OD_FCST_Right	Selected tracts	WM	OD	Y	Y
Mean OD in fornix on FA skeleton	No	25397		OD_FO	Selected tracts	WM	OD	Y	Y
Mean OD in genu of corpus callosum on FA skeleton	No	25394		OD_GCC	Selected tracts	WM	OD	Y	Y
Mean OD in inferior cerebellar peduncle on FA skeleton (left)	Yes	25403		OD_ICP_Left	Selected tracts	WM	OD	Y	Y
Mean OD in inferior cerebellar peduncle on FA skeleton (right)	Yes	25402		OD_ICP_Right	Selected tracts	WM	OD	Y	Y
Mean OD in medial lemniscus on FA skeleton (left)	Yes	25401		OD_ML_Left	Selected tracts	WM	OD	Y	Y
Mean OD in medial lemniscus on FA skeleton (right)	Yes	25400		OD_ML_Right	Selected tracts	WM	OD	Y	Y
Mean OD in middle cerebellar peduncle on FA skeleton	No	25392		OD_MCP	Selected tracts	WM	OD	Y	Y
Mean OD in pontine crossing tract on FA skeleton	No	25393		OD_PCT	Selected tracts	WM	OD	Y	Y
Mean OD in posterior corona radiata on FA skeleton (left)	Yes	25419		OD_PCR_Left	Selected tracts	WM	OD	Y	Y
Mean OD in posterior corona radiata on FA skeleton (right)	Yes	25418		OD_PCR_Left	Selected tracts	WM	OD	Y	Y
Mean OD in posterior limb of internal capsule on FA skeleton (left)	Yes	25411		OD_PLIC_Left	Selected tracts	WM	OD	Y	Y
Mean OD in posterior limb of internal capsule on FA skeleton (right)	Yes	25410		OD_PLIC_Right	Selected tracts	WM	OD	Y	Y
Mean OD in posterior thalamic radiation on FA skeleton (left)	Yes	25421		OD_PTR_Left	Selected tracts	WM	OD	Y	Y
Mean OD in posterior thalamic radiation on FA skeleton (right)	Yes	25420		OD_PTR_Right	Selected tracts	WM	OD	Y	Y
Mean OD in retrolenticular part of internal capsule on FA skeleton (left)	Yes	25413		OD_RPIC_Left	Selected tracts	WM	OD	Y	Y
Mean OD in retrolenticular part of internal capsule on FA skeleton (right)	Yes	25412		OD_RPIC_Right	Selected tracts	WM	OD	Y	Y
Mean OD in sagittal stratum on FA skeleton (left)	Yes	25423		OD_SS_Left	Selected tracts	WM	OD	Y	Y
Mean OD in sagittal stratum on FA skeleton (right)	Yes	25422		OD_SS_Right	Selected tracts	WM	OD	Y	Y
Mean OD in splenium of corpus callosum on FA skeleton	No	25396		OD_SCC	Selected tracts	WM	OD	Y	Y

Mean OD in superior cerebellar peduncle on FA skeleton (left)	Yes	25405		OD_SCP_Left	Selected tracts	WM	OD	Y	Y
Mean OD in superior cerebellar peduncle on FA skeleton (right)	Yes	25404		OD_SCP_Right	Selected tracts	WM	OD	Y	Y
Mean OD in superior corona radiata on FA skeleton (left)	Yes	25417		OD_SCR_Left	Selected tracts	WM	OD	Y	Y
Mean OD in superior corona radiata on FA skeleton (right)	Yes	25416		OD_SCR_Right	Selected tracts	WM	OD	Y	Y
Mean OD in superior fronto-occipital fasciculus on FA skeleton (left)	Yes	25435		OD_FOF_Left	Selected tracts	WM	OD	Y	Y
Mean OD in superior fronto-occipital fasciculus on FA skeleton (right)	Yes	25434		OD_FOF_Right	Selected tracts	WM	OD	Y	Y
Mean OD in superior longitudinal fasciculus on FA skeleton (left)	Yes	25433		OD_SLF_Left	Selected tracts	WM	OD	Y	Y
Mean OD in superior longitudinal fasciculus on FA skeleton (right)	Yes	25432		OD_SLF_Right	Selected tracts	WM	OD	Y	Y
Mean OD in tapetum on FA skeleton (left)	Yes	25439		OD_TP_Left	Selected tracts	WM	OD	Y	Y
Mean OD in tapetum on FA skeleton (right)	Yes	25438		OD_TP_Right	Selected tracts	WM	OD	Y	Y
Mean OD in uncinate fasciculus on FA skeleton (left)	Yes	25437		OD_UNC_Left	Selected tracts	WM	OD	Y	Y
Mean OD in uncinate fasciculus on FA skeleton (right)	Yes	25436		OD_UNC_Right	Selected tracts	WM	OD	Y	Y

Sources: https://biobank.ndph.ox.ac.uk/showcase/search.cgi; https://biobank.ndph.ox.ac.uk/showcase/ukb/docs/brain_mri.pdf;

https://biobank.ndph.ox.ac.uk/showcase/label.cgi?id=100

OSM 2: Life's essential 8 dietary component

The touchscreen questionnaire used for the UKB main investigation contained 29 diet-related topics and 18 questions about alcohol. The touchscreen survey inquired about the frequency and types of food consumed in the past year from various food categories including cooked vegetables, salad/raw vegetables, fresh fruit, dried fruit, oily fish, other fish, processed meats, poultry, beef, lamb, pork, cheese, salt added to food, tea, and water. The survey inquired about the preferred milk type, spread type, number of slices and type of bread consumed, number of bowls and type of breakfast cereal most frequently eaten, type of coffee cup most frequently used, avoidance of specific foods and food groups (eggs, dairy products, wheat, sugar), and age at last meat consumption for participants who reported never consuming processed meats, poultry, beef, lamb, pork, and cheese. During the primary evaluation phase, four dietary questions from the pilot experiment were slightly altered. The questions focused on avoiding specific foods and food groups, types of bread, types of spreads, and dietary variety.

The Healthy Diet Index (HDI) score incorporated various food groups by considering the amount and frequency of consumption per week to align with the recommendations outlined elsewhere [16]. However, those guidelines were modified to consider the data's availability in the UK biobank, as detailed in previous studies[17, 18]. The 24-hour dietary recall was utilized to validate the touchscreen questionnaire against UK Biobank participants. The study showed satisfactory agreement in ranking for each food group analyzed [19].

OSM 3: OLINK PROTEOMICS

Sources: [1, 17, 20-24]

The study used the Olink® Explore 1536 Proteomics platform to analyze 54,306 plasma samples from UK Biobank participant-visits. The platform uses Proximity Extension Assay (PEA) technology to measure 1,472 protein analytes, representing 1,463 unique proteins from inflammation, cancer, cardiometabolic, and neurological panels. Plasma samples were diluted in series and analyzed on four 384-well plates with four abundance blocks per 96 samples. Quality control was conducted using a Bioanalyzer, and sequencing on S4 flow cells v1.5 using a Novaseq600 machine with 35 cycles.

Olink quality controls were integrated into the workflow, with internal controls like Incubation Control (Inc Ctrl), extension controls, and amplification controls. The assay limit of detection (LOD) was determined by using triplicate negative controls on each plate and running a pooled plasma sample in triplicate as a plate control sample. Each panel included three proteins for quality control (QC) and correlation analysis: IL6, IL8 (CXCL8), and TNF.

The data was normalized within each batch, and adjustment factors were determined for each set. The original UKB-Olink dataset comprised 58,699 samples from 54,309 individuals. Samples were excluded for withdrawing from the study, unprocessed samples, and failing quality control. In addition, outliers were detected through principal component analysis (PCA) and analyzing the median and interquartile range (IQR) of NPX values across proteins per sample. The coefficient of variation within individuals for each protein was determined by analyzing duplicate

samples, with values ranging from 2.4% to 25%. For this current study, we utilized proteomic data from 3,953 UK Biobank participants that had neuroimaging and data for key variables.

OSM 4: ALZHEIMER'S DISEASE POLYGENIC RISK SCORE

PRS scores were developed and utilized on meta-analyzed GWAS summary statistics, with a focus on ancestry specificity. Data was sourced from external GWAS data for the Standard PRS set, and a combination of external and internal UK Biobank data for the Enhanced PRS set, using a Bayesian method. The Standard PRS Set, also known as the "UKB-Free" set, comprises 28 diseases and 8 quantitative traits. It was developed using external GWAS data and the methodology was outlined in the supplementary material of the primary publication by Thompson et al. in 2022 [25]. We selected AD PRS from the standard PRS set, which was initially identified in the PGS catalog available at https://www.pgscatalog.org. We chose a version of the AD PRS that included APOE.

OSM 5: Four-way decomposition models

Each plasma proteome biomarker was identified as a potential mediator or moderator in the association between poor cardiovascular health (CVH) and the risk of dementia from 2006 to 2021. The impact of $LE8_{z\_rev}$ exposure on white matter microstructural integrity markers was categorized into four groups based on the presence of a mediator that could interact with the exposure: (i) no mediation or interaction; (ii) interaction only; (iii) both mediation and interaction; and (iv) mediation only. This new method in Stata involves evaluating mediation and attributing effects to interactions, enabling the calculation of the four-way breakdown through parametric or semi-parametric regression models. Using ordinary least square linear regression models for both the outcome and the mediator/moderator equations, a total of 1,463 plasma proteome mediators were examined as viable alternatives. Mediation and interaction of the total effect of $LE8_{z\_rev}$ exposure on DTI and NODDI metric outcomes were tested using the Med4way

command [26] [https://github.com/anddis/med4way]. Age, sex, race (Non-White vs. White), SES z-score, household size and time elapsed between baseline assessment and the neuroimaging visit were considered as exogenous factors in a four-way decomposition analysis of the full sample. The significance level for each analysis was set at 0.05.

OSM 6: **Principal components analysis (PCA) of selected consistent mediating proteins**

PCA is a linear dimensionality reduction technique used in data preprocessing, analysis, and visualization. This is accomplished by mapping the data linearly onto a new coordinate system (principal components), which makes it possible to identify the directions that capture the most variation in the data. To plot data in two dimensions and visually identify clusters of strongly connected data points, many research use the first two main components. Nevertheless, the number of extracted components is often determined using the Kaiser rule (eigenvalue>1) or through examining the scree plot.

After selecting the consistent mediators for each of FA_{mean} and OD_{mean}, the common set of consistently mediating proteins between the two outcomes was determined, consisting of 9 plasma proteins (PROTcons_med). Those were chosen and placed as measured variables into a principal components analysis (PCA) model [27]. Using the Kaiser rule, a number of common principal components were extracted. Component loadings and residual variances were estimated, with the residual variance being labeled as uniqueness for each PROTcons_med. The following is a summary of the PCA model:

$$PROT_{cons\_medi} = \sum_{j=1}^{k} \lambda_{ij} * PCj + \phi_i$$

Where $PROT_{cons\_medi}$ is the standardized z-score for each selected Log2 transformed protein deemed to be consistent mediators for both FAmean and ODmean (k=9), λ_{ij} is the component loading for each $PROT_{cons\_medi}$ and each component PCj, PCj is the standardized z-score for each component j, and φ_i is the residual error, the squared value of which is the uniqueness. The sum of squared component loadings for each $PROT\_{}_{cons\_medi}$ is the communality or the common variance that is accounted for by the extracted components.

After extracting 3 principal components using the Kaiser rule, the component loadings were further rotated using an orthogonal rotation process known as varimax. Those 3 components (PC1, PC2 an PC3) could be interpreted and labeled accordingly, using an arbitrary cutoff point of 0.20 or more in absolute value for significant loading that would discriminate between the PC, with the goal of obtaining a simple structure. The component scores (*z*-scores) were predicted to reduce the 9 variables to the main 3 PC, which are also on a set of standardized z-scores. Prediction is accomplished with the regression method.

Supplementary Table 2. Varimax rotated two-factor solution of PROTcons_med$_i$, using 9 selected consistent mediating proteins as measured variables.

	Component loadings, λ_{ij}			Uniqueness, φ_i
	PC1	PC2	PC3	
LEP	-0.07	**-0.45***	**+0.42***	0.35
CPM	**+0.42***	-0.04	+0.11	0.43
F9	**+0.32***	-0.04	**+0.30***	0.45
FGF21	**+0.46***	-0.12	**-0.23***	0.44
PRSS8	**+0.51***	+0.15	+0.01	0.34
LILRA5	+0.00	+0.05	**+0.74***	0.28
CA14	+0.06	**+0.64***	-0.07	0.33
VWA1	**+0.41***	-0.00	-0.01	0.55
BAIAP2	**+0.24***	-0.01	+0.12	0.78
WFIKKN2	-0.10	**+0.59***	**+0.32***	0.39
Eigenvalue	3.24	1.42	1.00	
% var explained	32.2	14.2	10.0	

Note: See list of abbreviations.

*Component loading>0.20 in absolute value. Principal components were labeled as follows: "PC1: 6 proteins of 9, PC2: 3 protein out of 9, PC3: 4 proteins out of 9, based on the combination of significantly high factor loadings and the corresponding measured variables or $PROT_{cons\_medi}$. It is worth noting that PC2 was inversely related to LEP, unlike PC3, with comparable component loadings. A similar pattern was observed for FGF21 between PC1 and PC3. All other strong loadings were positive.

OSM 7: Supplementary literature review on the nine selected consistent protein mediators (between CVH and FA_{mean}; CVH and OD_{mean}) and their relationship with CVH and dementia traits, including neuroimaging markers

LEP

Recent data on older adults with cerebral small vessel disease found that higher leptin levels were associated with lower gray matter and total brain volumes in men[28]. However, in the Framingham study, higher leptin levels were associated with higher total cerebral brain volume[29]. The relationship between leptin and brain volumes and markers of brain white matter microstructural integrity is still being studied. Leptin may be involved in hippocampus synaptic plasticity, controlling neuron excitability, and acting as an anti-apoptotic agent in stressful conditions [30-33]. It has been found to enhance the uptake of beta-amyloid into the cell through Apolipoprotein-E, leading to a decrease in its accumulation outside the cell[34]. Leptin and insulin work together to decrease the excessive phosphorylation of tau, a key element of the neurofibrillary tangle in Alzheimer's disease (AD) [35]. Treating transgenic mice with leptin led to enhancements in memory skills[36]. However, leptin resistance and obesity are prone to develop due to blood leptin levels exceeding 25-30 ng/mL, which alter blood brain barrier permeability [37]. Aljaloud et al.'s 2022 study found no significant difference in physical activity levels between lean and overweight/obese adults[38]. However, the overweight/obese group had higher leptin, insulin, and C-reactive protein levels [38]. Vigorous activity was positively correlated with HDL and negatively with LDL and the ARA/EPA ratio [38]. Mendoza-Herrera et al.'s 2021 study suggested addressing leptin resistance through diet can counteract obesity [39]. Dashti et al.'s 2015 review highlighted the link between short sleep duration and obesity, type 2 diabetes, hypertension, and cardiovascular disease[40]. A systematic review found a correlation between smoking and serum leptin/ghrelin levels in 11,336 patients [40]. Shramco et al.'s 2023 study found the most common variant of the ADIPOQ and LEP genes in patients with metabolic syndrome[41]. Khaki-Khatibi et al.'s 2023 study found an association between leptin gene polymorphisms with an increased risk of cardiovascular disease[42].

CPM

A pilot study comparing gene expression profiles in Alzheimer's disease (AD) patients and control probands revealed 20 potential genes that differ in AD lymphocytes compared to control probands [43]. These genes include defensin, histocompability complex enhancer-binding protein, carboxypeptidase M (or CPM), and the Fc segment of IgE [43]. The down-regulation of defensin and alpha2c-adrenoceptor genes was confirmed by real-time quantitative reverse transcription-polymerase chain reaction analysis [43].

The study suggests these altered expression profiles may be related to AD lymphocyte abnormalities [43]. There is very little evidence of an association between CPM and cardiovascular disease.

F9

The Alzheimer peptide β-amyloid interacts with fibrinogen and blood coagulation factor XII, leading to increased clotting, abnormal clot formation, persistent fibrin deposition, and generation of proinflammatory molecules. These events can damage neurons and contribute to cognitive decline in Alzheimer's disease patients [44]. A study using SomaScan assay found that protein levels of coagulation factor XI are significantly increased in patients diagnosed with probable Alzheimer's disease compared to cognitively healthy subjects or mild cognitive impairment [45]. Factors IX and XI were significant predictors of Alzheimer's-type diagnosis, with an increase in both factors associated with a reduction in cognitive function[45]. The study justifies further investigations into biological pathways involving coagulation/anticoagulation factors in relation to dementia, including dementia resulting from Alzheimer's-type neurodegeneration[45]. Nevertheless, in a third study, no strong relationships between higher FVIII:C levels and white matter hyperintensity burden or cognitive function were detected, neither in cross-sectional nor in longitudinal analyses [46]. Thus, only limited evidence was available for an association between F9 (or Factor IX) and AD or dementia traits. There is very little evidence of an association between CPM and cardiovascular disease.

FGF21

FGF21 is a circulating endocrine hormone secreted by the liver primarily in fasting conditions and exerts its effects after binding to FGFR1 and co-receptor, β-klotho (KLB) [47-49]. It is involved in regulating energy via glucose and lipid metabolism. A recent review focuses on FGF21's role as a neuroprotectant and potential metabolic regulator, as well as its potential as an emerging candidate for combating metabolic stress-induced neurodegenerative abnormalities[48]. The system consists of FGFs and their receptors (FGFRs), which may hold great potential as a new biological marker in the diagnosis of dementia and other cognitive disorders[47-49]. Fibroblast growth factor 21 (FGF21) has been found to ameliorate neurodegeneration in rat and cellular models of Alzheimer's disease (AD) [47-49] . A study, which was conducted in vivo and in vitro, found that FGF21 exerted diverse effects on cell apoptosis, tau hyperphosphorylation, and oxidative stress induced by amyloid β-peptide 25-35[47] . The results also suggested that the regulation of protein phosphatase 2A / mitogen-activated protein kinases / hypoxia-inducible factor-1α pathway may play a key role in mediating the neuroprotective effects of FGF21 against AD-like pathologies[47].

PRSS8

A study has created the largest cerebrospinal fluid pQTL atlas by analyzing 7,028 proteins in 3,107 samples. The atlas identified 3,373 independent associations for 1,961 proteins, including 2,448 novel pQTLs, 1,585 unique to CSF[50]. The researchers also identified pleiotropic regions enriched for neuron-specificity and neurological development [50]. They integrated this atlas with the latest Alzheimer's disease GWAS, identifying 42 putative causal proteins for AD, 15 of which have drugs available. PRSS8 was among those causal proteins for AD [50]. The findings will help understand biology and identify causal and druggable proteins for brain and neurological traits and was corroborated by at least two other studies [51, 52]. Prostasin has been suggested as a candidate biomarker for hypertension based on their role in the activation of epithelial sodium channel in the kidney[53]. In rats, injection of adenovirus with human prostasin resulted in increase in aldosterone and subsequently the animals developed hypertension[54]. In humans, genetic polymorphisms in the PRSS8 gene have been associated with variability in blood pressure[55]. In this report, we link plasma levels of prostasin with elevated blood pressure. In humans, plasma PRSS8 has been associated with increased risk of both prevalent and incident diabetes[55]. While the mechanisms linking PRSS8 with diabetes is unclear, animal studies suggest that PRSS may effect insulin sensitivity through Toll-like receptor 4 signaling-mediated regulation of inflammation[56]. There is some conflicting evidence of the direction of association between PRSS8 and diabetes, where in the human studies PRSS8 is shown to increase the risk of diabetes[57], while animal studies suggest a protective role of PRSS8 on glucose homeostasis[56]. In the current report, PRSS8 was associated with greater plasma glucose, consistent with the prior human study. In addition to cardiometabolic risk factors, plasma PRSS8 has been reported to be suppressed with greater leisure-time physical activity[58].

LILRA5

Three studies from our review of the recent literature involved LILRA5 in dementia traits[59-61]. The first study examining the genetic mechanisms of Alzheimer's disease (LOAD) found that 216 unique significant genes were identified, including 72 with no previously reported LOAD GWAS associations[59]. Eight additional genes were found to be significantly associated with LOAD[59]. The proportion of SNP-based heritability was highly enriched in genes identified by GReX analysis[59]. Another study explored shared genetic susceptibility between Alzheimer's disease (AD) and multiple sclerosis (MS) to identify pathological mechanisms shared between neurodegeneration and the immune system [60]. Gaussian causal mixture modelling (MiXeR) was used to characterize the genetic architecture and overlap between AD and MS[60]. Annotated genes in shared loci were enriched in molecular signaling pathways involved in inflammation and the structural organization of neurons[60]. The results from a third study suggest

that genetic variability in response to amyloid beta deposition influences Alzheimer's disease risk, with an improved RNA-seq-derived network predicting at least four new risk genes for the disease[61]. Whole blood gene expression studies have shown differential expression of *LILRA5* in association with BMI [62], and myocardial infarction [63]. There is also evidence linking LILRA5 protein levels in association with cardiovascular disease. In a study of 826 male participants 70 years and older, plasma LILRA5 was associated with increased risk of incident heart failure[64]. This is consistent with what is reported in this study where higher plasma LILRA5 was found in association with cardiovascular disease risk factors.

CA14

Carbonic anhydrase (CA) is an enzyme is involved in include gas exchange, ion transport, and acid-based control [65]. It has many isoforms which catalyzes the reversible hydration of carbon dioxide [66, 67]. The administration of topiramate, a mitochondrial CA inhibitor, to type II diabetic animal models, resulted in disruption of the blood-brain barrier and blood-retinal barrier [68]. A study using animal models found CA14 was abundantly expressed in the liver and had a role in bicarbonate homeostasis, protecting the liver from bile acid toxicity [67]. Increased CA was associated with hypertensive obstructive sleep apnea [66]. Gait speed and grip strength reduction were substantially correlated with CA3 in human samples [69]. Compared non-smokers and non-diabetics, the activity of CA activity was lower in smokers and diabetics [65]. The findings of these studies support the link between CA and cardiovascular health while also pointing to possible benefits for diabetes treatment and disease prevention.

VWA1

VWA1 (Von Willebrand Factor A-Domain Containing 1) is an extracellular matrix protein that contains the N-terminal von Willebrand factor A domain followed by two fibronectin type III repeats. This protein coding gene is expressed in the cartilage and in the basement membranes of peripheral nerves (endoneurium), skeletal muscle (endomysium), and the central nervous system vasculature[70]. Coronary artery calcification scores are associated with VWA1 [71].VWA1 is one of the genes that can mediate the negative effects of aging and obesity, and the protective effect of exercise. Expression of VWA1 in mouse cardiac endothelial cells have shown to be significantly upregulated by aging mice and high fat diet induced obese mice and downregulated by exercise training[72]. It has been suggested that WARP (von Willebrand A domain-related protein) have an important role in maintaining the blood-brain barrier. WARP was detected in the vessels associated with neural tissue of the brainstem and cerebellar cortex[73].Localization of WARP in the human inner ear vasculature suggests an important role in maintaining its integrity[73]. These studies suggest an association of VWA1 and cardiovascular health. VWA1 involvement in dementia traits has been shown in at least one prior animal

study using 5XFAD humanized mutant mice and Trem2 knockout mice to study Alzheimer's disease (AD)-related pathology has identified differentially expressed genes and differential transcript usage in hippocampal cell types [74]. The researchers also identified three significant canonical pathways that appeared in multiple cell types and overlapped between the two models [74]. The findings highlight the importance of energy imbalance and inflammatory processes in specific hippocampal cell types, including neurons and glial cells, in AD pathology development. Further research is needed to characterize these findings and understand the role of WARP in AD pathology [74].

BAIAP2

Several studies have involved BAIAP2 in dementia traits [75-77]. Researchers have identified new risk factors for behavioral variant frontotemporal dementia (bvFTD) by analyzing the genetic underpinnings of this selective regional vulnerability [75]. They generated a protein interaction network of 63 bvFTD risk genes and characterized this network using gene expression data from healthy and diseased human brain tissue [75]. The results showed that bvFTD network genes show enriched expression across the human lifespan in vulnerable neuronal populations, implicated in cell signaling, cell cycle, immune function, and development [75]. Five genes, BAIAP2, ERBB3, POU2F2, SMARCA2, and CDC37, appear to be novel bvFTD risk loci [75]. The cumulative burden of common genetic variation in an interacting protein network expressed in specific brain regions across the lifespan may influence susceptibility to bvFTD [77]. In Huntington disease (HD), a large-scale Huntingtin protein interaction network implicates Rho GTPase signaling pathways in HD[77]. The researchers validated roles for HIPs in mutant HTT toxicity by showing that Rho GTPase signaling components, BAIAP2, EZR, PIK3R1, PAK2, and RAC1, are modifiers of mutant HTT toxicity[77]. In a third study, Following the confirmation of three proteins—Baiap2, Gpr37l1, and Nptx1—as novel tau-interacting partners, it is now postulated that there might be a possible involvement in tau pathology based on their established roles and consequences in neurodegenerative or psychiatric illnesses[76]. Several genes including BAIAP2 have demonstrated involvement in cardiovascular disease [78] and expressed higher in those with non-ST elevation acute coronary syndrome [79].

WFIKKN2

At least one study showing involvement of WFIKKN2 in AD was carried out comparing AD patients and healthy controls using differentially expressed genes (DEGs) from the entorhinal cortex and hippocampal regions, as well as gene expression data from the Gene Expression Omnibus database [80] . After performing functional enrichment analysis, such as Gene Ontology (GO) and pathway analyses, a protein-protein interaction network was built[80] . In the entorhinal cortex and hippocampal regions, respectively, the study found 195 and 58 down-regulated genes and 251 up-regulated genes, or DEGs. In the entorhinal cortex and hippocampal regions, the study also discovered 11 hub genes, and also identified 3 hub genes as new possibilities for roles in AD pathophysiology[80] . The

findings may shed light on possible treatment options as well as the pathogenic mechanism behind AD [80]. WFIKKN2 has been shown to be associated with cardiovascular health such as hemoglobin A1c and fasting glucose [81]. Additionally, WFIKKN2 has been associated as an obesity-related protein [82] and isolated as associated with atrial fibrillation, among other proteins [83].

Supplementary Table 3. DESCRIPTION OF TOP HITS FOR PROTEOMIC MEDIATORS BETWEEN CARDIOVASCULAR HEALTH AND DEMENTIA

Symbol	Protein/Gene name	Function	Recent papers with relevance to cardiovascular health	Recent papers with relevance to dementia
**LEP**	**Leptin**	This gene encodes a protein that is secreted by white adipocytes into the circulation and plays a major role in the regulation of energy homeostasis. Circulating leptin binds to the leptin receptor in the brain, which activates downstream signaling pathways that inhibit feeding and promote energy expenditure. This protein also has several endocrine functions, and is involved in the regulation of immune and inflammatory responses, hematopoiesis, angiogenesis, reproduction, bone formation and wound healing. Mutations in this gene and its regulatory regions cause severe obesity and morbid obesity with hypogonadism in human patients. A mutation in this gene has also been linked to type 2 diabetes mellitus development. [provided by RefSeq, Aug 2017]	**Physical activity**: [38]<br>**Diet**: [39]<br>**Sleep:** [40]<br>**Smoking:** [84]<br>**Obesity:** [41, 42]<br>**Diabetes/glucose**: [41]<br>**Lipids**: [41]<br>**Blood pressure/hypertension**: [41]<br>**Cardiometabolic risk or cardiovascular disease**: [41, 42]	[28, 29, 85-89]
**CPM**	**Carboxypeptidase M**	The protein encoded by this gene is a membrane-bound arginine/lysine carboxypeptidase. Its expression is associated with monocyte to macrophage differentiation. This encoded protein contains hydrophobic regions at the amino and carboxy termini and has 6 potential asparagine-linked glycosylation sites. The active site residues of carboxypeptidases A and B are conserved in this protein. Three alternatively spliced transcript variants encoding the same protein have been described for this gene. [provided by RefSeq, Jul 2008]	**Physical activity**: n/a<br>**Diet**: n/a<br>**Sleep:** n/a<br>**Smoking:** n/a<br>**Obesity:** n/a<br>**Diabetes/glucose**: n/a<br>**Lipids**: n/a<br>**Blood pressure/hypertension**: [90]<br>**Cardiometabolic risk or cardiovascular disease**:[90]	[43]
**F9**	**Coagulation Factor IX**	This gene encodes vitamin K-dependent coagulation factor IX that circulates in the blood as an inactive zymogen. This	**Physical activity**: n/a<br>**Diet**: n/a<br>**Sleep:** n/a	[44-46, 92]

		factor is converted to an active form by factor XIa, which excises the activation peptide and thus generates a heavy chain and a light chain held together by one or more disulfide bonds. The role of this activated factor IX in the blood coagulation cascade is to activate factor X to its active form through interactions with Ca+2 ions, membrane phospholipids, and factor VIII. Alterations of this gene, including point mutations, insertions and deletions, cause factor IX deficiency, which is a recessive X-linked disorder, also called hemophilia B or Christmas disease. Alternative splicing results in multiple transcript variants encoding different isoforms that may undergo similar proteolytic processing. [provided by RefSeq, Sep 2015]	**Smoking:** n/a<br>**Obesity:** n/a<br>**Diabetes/glucose**: n/a<br>**Lipids**: n/a<br>**Blood pressure/hypertension**: n/a<br>**Cardiometabolic risk or cardiovascular disease**:[91]	
**FGF21**	Fibroblast Growth Factor 21	Theis gene encodes a member of the fibroblast growth factor (FGF) family. FGF family members possess broad mitogenic and cell survival activities and are involved in a variety of biological processes. This protein is a secreted endocrine factor that functions as a major metabolic regulator. The encoded protein stimulates the uptake of glucose in adipose tissue. [provided by RefSeq, Mar 2016]	**Physical activity**: [93]<br>**Diet**: [94]<br>**Sleep:** n/a<br>**Smoking:**<br>**Obesity:** [95-99]<br>**Diabetes/glucose**: [94, 98]<br>**Lipids**: [98, 99]<br>**Blood pressure/hypertension**: [99]<br>**Cardiometabolic risk or cardiovascular disease**: [99]	[47-49]
**PRSS8**	Serine protease 8	This gene encodes a member of the peptidase S1 or chymotrypsin family of serine proteases. The encoded preproprotein is proteolytically processed to generate light and heavy chains that associate via a disulfide bond to form the heterodimeric enzyme. This enzyme is highly expressed in prostate epithelia and is one of several proteolytic enzymes found in seminal fluid. This protease exhibits trypsin-like substrate specificity, cleaving protein substrates at the carboxyl terminus of lysine or arginine residues. The encoded protease partially mediates proteolytic activation of the epithelial sodium channel, a regulator of sodium balance, and may also play a role in epithelial barrier formation. [provided by RefSeq, Feb 2016]	**Physical activity**: n/a<br>**Diet**: n/a<br>**Sleep: n/a**<br>**Smoking:** [100]<br>**Obesity:** n/a<br>**Diabetes/glucose**: [101]<br>**Lipids**: n/a<br>**Blood pressure/hypertension**: n/a<br>**Cardiometabolic risk or cardiovascular disease**: [102]	[50-52]

**LILRA5**			**Physical activity**: n/a<br>**Diet**: n/a<br>**Sleep:** n/a<br>**Smoking:** n/a<br>**Obesity:** [62]<br>**Diabetes/glucose**: n/a<br>**Lipids**: n/a<br>**Blood pressure/hypertension**: [63]<br>**Cardiometabolic risk or cardiovascular disease**: [63]	[59-61]
**CA14**	Carbonic anhydrase 14	Carbonic anhydrases (CAs) are zinc metalloenzymes that reversibly hydrate carbon dioxide, playing a role in various biological processes like respiration, calcification, and bone resorption. They vary in tissue distribution and localization, with different physiologic roles.	**Physical activity**: [69, 103]<br>**Diet**: [68]<br>**Smoking:** [65]<br>**Sleep:** [66]<br>**Obesity:** [68, 104, 105]<br>**Diabetes/glucose**: [68]<br>**Lipids**: [67]<br>**Blood pressure/hypertension**: [106]<br>**Cardiometabolic risk or cardiovascular disease**:[106]	[107-111]
**VWA1**	von Willebrand factor 1	VWA1 belongs to the von Willebrand factor (VWF; MIM 613160) A (VWFA) domain superfamily of extracellular matrix proteins and appears to play a role in cartilage structure and function [112] supplied by OMIM, Nov 2010]	**Physical activity**: [72]<br>**Diet**: n/a<br>**Sleep:** n/a<br>**Smoking:** n/a<br>**Obesity:** n/a<br>**Diabetes/glucose**:<br>**Lipids**: n/a<br>**Blood pressure/hypertension**: n/a<br>**Cardiometabolic risk or cardiovascular disease**: [71]	[74]
**BAIAP2**	BAR/IMD domain containing adaptor protein 2	The protein encoded by this gene has been identified as a brain-specific angiogenesis inhibitor (BAI1)-binding protein. This adaptor protein links membrane bound G-proteins to cytoplasmic effector proteins. This protein functions as an insulin receptor tyrosine kinase substrate and suggests a role for insulin in the central nervous system. It also associates with a downstream effector of Rho small G proteins, which is associated with the formation of stress fibers and cytokinesis. This protein is involved in lamellipodia and filopodia formation in motile cells and may affect neuronal growth-cone guidance. This protein has also been identified as interacting with the dentatorubral-pallidoluysian atrophy	**Physical activity**: n/a<br>**Diet**: n/a<br>**Sleep:** [113]<br>**Smoking:** n/a<br>**Obesity:** [114]<br>**Diabetes/glucose**: [78, 115]<br>**Lipids**: n/a<br>**Blood pressure/hypertension**: n/a<br>**Cardiometabolic risk or cardiovascular disease**:[78]	[75-77]

		gene, which is associated with an autosomal dominant neurodegenerative disease. Alternative splicing results in multiple transcript variants encoding distinct isoforms.[provided by RefSeq, Jan 2009]		
**WFIKKN2**	WAP, follistatin/kazal, immunoglobulin, kunitz and netrin domain containing 2	The WFIKKN1 protein contains a WAP domain, follistatin domain, immunoglobulin domain, two tandem Kunitz domains, and an NTR domain. This gene encodes a WFIKKN1-related protein which has the same domain organization as the WFIKKN1 protein. The WAP-type, follistatin type, Kunitz-type, and NTR-type protease inhibitory domains may control the action of multiple types of proteases. [provided by RefSeq, Jul 2008]	**Physical activity**: n/a<br>**Diet**: n/a<br>**Sleep:** n/a<br>**Smoking:** n/a<br>**Obesity:** [116]<br>**Diabetes/glucose**: [81]<br>**Lipids**: n/a<br>**Blood pressure/hypertension**: n/a<br>**Cardiometabolic risk or cardiovascular disease**: n/a	[80]

Protein abbreviations are found at https://www.ncbi.nlm.nih.gov/gene/.

OSM 8: OLINK Insight and STRING analysis methods and results

Methods:

In this part of the analysis, only global average DTI and NODDI measures that displayed a significant TE were used. The results were analyzed using the OLINK insight pathways browser (https://insight.olink.com/) to identify the most frequently used paths by the mediators, as shown in a set of interconnected pathways. Specific pathways deemed unique to each DTI/NODDI metric and common pathways between them were also determined. Moreover, the mediators showing statistically significant PIE for each measure coupled with a with significant TE were analyzed using the Search Tool for the Retrieval of Interacting Genes/Proteins (STRING: (https:// string-db.org)) and k-means clustering with a maximum of 10

clusters. This provided additional details on proteomic biomarkers identified as significant mediators using PIE, along with their grouping or functional relationships with the top mediators. Gene ontology (GO) pathways in these clusters were analyzed and top pathways were identified partly using the lowest false discovery rate coupled with a strength value greater than 1. Strength in GO indicates the degree of confidence or support for the functional connection between certain genes or words, as determined by existing biological data. Detailed results are presented in: https://github.com/baydounm/UKB-paper13-supplementarydata.

Results:

When examining all statistically significant mediators with PIE $p<0.05$ (**Appendices VIII** through **X**), 212 plasma proteins were identified as mediators between poor CVH ($LE8_{z\_rev}$) and FA_{mean}, and 142 plasma proteins were identified as mediators between poor CVH and OD_{mean}. A total of 1002 pathways were identified based on the OLINK insight pathway analysis for mediators between poor CVH and FA_{mean} and/or OD_{mean}, with the full list of key mediators having significant PIE shown in **Appendix X**, and the list of pathways for FA_{mean}, OD_{mean} and their intersection shown in **supplementary datasheet 3** (**Appendix IX**). For FA_{mean} alone 821 pathways were identified, while for OD_{mean}, 525 unique pathways were identified, and for both phenotypic outcomes, while 339 pathways were in common between the two phenotypic outcomes [**Appendix VIII**; **supplementary Figure 4, panels A-C**]. These pathways when all unique proteins (k=313) were entered into the OLINK insight pathway (i.e for both FA_{mean} and OD_{mean}) belonged to the domains of the immune system, signal transduction, metabolism, disease (one pathway directly related to Alzheimer's Disease), protein metabolism, hemostasis, apoptosis, cellular response to stress, and membrane trafficking among others (See: [**Appendix VIII**; **supplementary Figure 4, panel C** ; See also: https://github.com/baydounm/UKB-paper13-supplementarydata). Furthermore, STRING analysis results are presented in **Figure S4**, panels **D** and **E**, focusing on the largest cluster uncovered for FA_{mean} and OD_{mean} four-way decomposition findings. In addition, GO biological, molecular and cellular components pathways for each of these clusters (i.e. for FA_{mean} and

OD_{mean}), were obtained and presented as supplementary information in https://github.com/baydounm/UKB-paper13-supplementarydata, and ranked according to FDR,gene count and signal strength. For OD_{mean}, the largest cluster was automatically labelled as “Cytokine-cytokine receptor interaction”, while several GO pathways identified as being the strongest with lowest FDR (GO biological: cell surface receptor signaling pathway; GO molecular: signaling receptor regulator activity; GO cellular component: Extracellular region). For FA_{mean}, while the largest cluster was not automatically labelled, clear patterns emerged regarding the strongest and most significant GO pathways (GO biological: transmembrane receptor protein tyrosine kinase signaling pathway; GO molecular: signaling receptor binding; GO cell component: Extracellular space).

SUPPLEMENTARY REFERENCES

[1] Beydoun MA, Beydoun HA, Hu YH, Li Z, Georgescu MF, Noren Hooten N, et al. Mediating and moderating effects of plasma proteomic biomarkers on the association between poor oral health problems and brain white matter microstructural integrity: the UK Biobank study. Mol Psychiatry. 2024.

[2] Beydoun MA, Beydoun HA, Hu YH, Li Z, Wolf C, Meirelles O, et al. Infection burden and its association with neurite orientation dispersion and density imaging markers in the UK Biobank. Brain Behav Immun. 2024;115:394-405.

[3] Beydoun MA, Beydoun HA, Gale SD, Hedges D, Weiss J, Li Z, et al. Cardiovascular health, infection burden and their interactive association with brain volumetric and white matter integrity outcomes in the UK Biobank. Brain Behav Immun. 2023;113:91-103.

[4] Gossai A, Waterboer T, Nelson HH, Michel A, Willhauck-Fleckenstein M, Farzan SF, et al. Seroepidemiology of Human Polyomaviruses in a US Population. American journal of epidemiology. 2016;183:61-9.

[5] Badji A, Cohen-Adad J, Girouard H. Relationship Between Arterial Stiffness Index, Pulse Pressure, and Magnetic Resonance Imaging Markers of White Matter Integrity: A UK Biobank Study. Front Aging Neurosci. 2022;14:856782.

[6] Andersson JL, Sotiropoulos SN. Non-parametric representation and prediction of single- and multi-shell diffusion-weighted MRI data using Gaussian processes. Neuroimage. 2015;122:166-76.

[7] Andersson JLR, Sotiropoulos SN. An integrated approach to correction for off-resonance effects and subject movement in diffusion MR imaging. Neuroimage. 2016;125:1063-78.

[8] Nir TM, Jahanshad N, Villalon-Reina JE, Isaev D, Zavaliangos-Petropulu A, Zhan L, et al. Fractional anisotropy derived from the diffusion tensor distribution function boosts power to detect Alzheimer's disease deficits. Magn Reson Med. 2017;78:2322-33.

[9] Mori S, Oishi K, Jiang H, Jiang L, Li X, Akhter K, et al. Stereotaxic white matter atlas based on diffusion tensor imaging in an ICBM template. Neuroimage. 2008;40:570-82.

[10] Smith SM, Jenkinson M, Johansen-Berg H, Rueckert D, Nichols TE, Mackay CE, et al. Tract-based spatial statistics: voxelwise analysis of multi-subject diffusion data. Neuroimage. 2006;31:1487-505.

[11] Daducci A, Canales-Rodriguez EJ, Zhang H, Dyrby TB, Alexander DC, Thiran JP. Accelerated Microstructure Imaging via Convex Optimization (AMICO) from diffusion MRI data. Neuroimage. 2015;105:32-44.

[12] Zhang Y, Brady M, Smith S. Segmentation of brain MR images through a hidden Markov random field model and the expectation-maximization algorithm. IEEE Trans Med Imaging. 2001;20:45-57.

[13] de Groot M, Vernooij MW, Klein S, Ikram MA, Vos FM, Smith SM, et al. Improving alignment in Tract-based spatial statistics: evaluation and optimization of image registration. Neuroimage. 2013;76:400-11.

[14] Beydoun MA, Hossain S, Tajuddin SM, Canas JA, Kuczmarski M, Beydoun HA, et al. Vitamin D Metabolism-Related Gene Haplotypes and Their Association with Metabolic Disturbances Among African-American Urban Adults. Sci Rep. 2018;8:8035.

[15] Liang H, Tao L, Ford EW, Beydoun MA, Eid SM. The patient-centered oncology care on health care utilization and cost: A systematic review and meta-analysis. Health Care Manage Rev. 2018.

[16] Mozaffarian D. Dietary and Policy Priorities for Cardiovascular Disease, Diabetes, and Obesity: A Comprehensive Review. Circulation. 2016;133:187-225.

[17] Beydoun MA, Beydoun HA, Hu YH, Maino Vieytes CA, Noren Hooten N, Song M, et al. Plasma proteomic biomarkers and the association between poor cardiovascular health and incident dementia: The UK Biobank study. Brain Behav Immun. 2024;119:995-1007.

[18] Beydoun MA, Beydoun HA, Fanelli-Kuczmarski MT, Weiss J, Georgescu MF, Meirelles O, et al. Pathways explaining racial/ethnic and socio-economic disparities in dementia incidence: the UK Biobank study. Aging (Albany NY). 2023;15:9310-40.

[19] Bradbury KE, Young HJ, Guo W, Key TJ. Dietary assessment in UK Biobank: an evaluation of the performance of the touchscreen dietary questionnaire. J Nutr Sci. 2018;7:e6.
[20] Sun BB, Chiou J, Traylor M, Benner C, Hsu YH, Richardson TG, et al. Plasma proteomic associations with genetics and health in the UK Biobank. Nature. 2023;622:329-38.
[21] Wik L, Nordberg N, Broberg J, Bjorkesten J, Assarsson E, Henriksson S, et al. Proximity Extension Assay in Combination with Next-Generation Sequencing for High-throughput Proteome-wide Analysis. Mol Cell Proteomics. 2021;20:100168.
[22] Petrera A, von Toerne C, Behler J, Huth C, Thorand B, Hilgendorff A, et al. Multiplatform Approach for Plasma Proteomics: Complementarity of Olink Proximity Extension Assay Technology to Mass Spectrometry-Based Protein Profiling. J Proteome Res. 2021;20:751-62.
[23] Beydoun MA, Beydoun HA, Noren Hooten N, Meirelles O, Li Z, El-Hajj ZW, et al. Hospital-treated prevalent infections, the plasma proteome and incident dementia among UK older adults. iScience. 2023;26:108526.
[24] Beydoun HA, Beydoun MA, Noren Hooten N, Weiss J, Li Z, Georgescu MF, et al. Mediating and moderating effects of plasma proteomic biomarkers on the association between poor oral health problems and incident dementia: The UK Biobank study. Geroscience. 2024;46:5343-63.
[25] Thompson DJ, Wells D, Selzam S, Peneva I, Moore R, Sharp K, et al. UK Biobank release and systematic evaluation of optimised polygenic risk scores for 53 diseases and quantitative traits. medRxiv. 2022:2022.06.16.22276246.
[26] Discacciati A, Bellavia A, Lee JJ, Mazumdar M, Valeri L. Med4way: a Stata command to investigate mediating and interactive mechanisms using the four-way effect decomposition. Int J Epidemiol. 2018.
[27] Sharma S. Applied multivariate techniques. USA: Wiley; 1996.
[28] Arnoldussen IAC, Gustafson DR, Leijsen EMC, de Leeuw FE, Kiliaan AJ. Adiposity is related to cerebrovascular and brain volumetry outcomes in the RUN DMC study. Neurology. 2019;93:e864-e78.
[29] Lieb W, Beiser AS, Vasan RS, Tan ZS, Au R, Harris TB, et al. Association of plasma leptin levels with incident Alzheimer disease and MRI measures of brain aging. JAMA. 2009;302:2565-72.
[30] Harvey J, Solovyova N, Irving A. Leptin and its role in hippocampal synaptic plasticity. Prog Lipid Res. 2006;45:369-78.
[31] Shanley LJ, Irving AJ, Harvey J. Leptin enhances NMDA receptor function and modulates hippocampal synaptic plasticity. J Neurosci. 2001;21:RC186.
[32] Doherty GH, Oldreive C, Harvey J. Neuroprotective actions of leptin on central and peripheral neurons in vitro. Neuroscience. 2008;154:1297-307.
[33] Guo Z, Jiang H, Xu X, Duan W, Mattson MP. Leptin-mediated cell survival signaling in hippocampal neurons mediated by JAK STAT3 and mitochondrial stabilization. J Biol Chem. 2008;283:1754-63.
[34] Fewlass DC, Noboa K, Pi-Sunyer FX, Johnston JM, Yan SD, Tezapsidis N. Obesity-related leptin regulates Alzheimer's Abeta. FASEB J. 2004;18:1870-8.
[35] Greco SJ, Sarkar S, Johnston JM, Zhu X, Su B, Casadesus G, et al. Leptin reduces Alzheimer's disease-related tau phosphorylation in neuronal cells. Biochem Biophys Res Commun. 2008;376:536-41.
[36] Tezapsidis N, Johnston JM, Smith MA, Ashford JW, Casadesus G, Robakis NK, et al. Leptin: a novel therapeutic strategy for Alzheimer's disease. J Alzheimers Dis. 2009;16:731-40.
[37] Gruzdeva O, Borodkina D, Uchasova E, Dyleva Y, Barbarash O. Leptin resistance: underlying mechanisms and diagnosis. Diabetes Metab Syndr Obes. 2019;12:191-8.
[38] Aljaloud KS, Hughes AR, Galloway SDR. Impact of Physical Activity on Adiposity and Risk Markers for Cardiovascular and Metabolic Disease. Am J Mens Health. 2022;16:15579883221092289.
[39] Mendoza-Herrera K, Florio AA, Moore M, Marrero A, Tamez M, Bhupathiraju SN, et al. The Leptin System and Diet: A Mini Review of the Current Evidence. Front Endocrinol (Lausanne). 2021;12:749050.
[40] Dashti HS, Scheer FA, Jacques PF, Lamon-Fava S, Ordovas JM. Short sleep duration and dietary intake: epidemiologic evidence, mechanisms, and health implications. Adv Nutr. 2015;6:648-59.

[41] Shramko, II, Ageeva ES, Maliy KD, Repinskaya IN, Tarimov CO, Fomochkina, II, et al. Association between Adiponectin and Leptin Receptor Genetic Polymorphisms and Clinical Manifestations of Metabolic Syndrome. J Diabetes Res. 2022;2022:9881422.
[42] Khaki-Khatibi F, Shademan B, Gholikhani-Darbroud R, Nourazarian A, Radagdam S, Porzour M. Gene polymorphism of leptin and risk for heart disease, obesity, and high BMI: a systematic review and pooled analysis in adult obese subjects. Horm Mol Biol Clin Investig. 2023;44:11-20.
[43] Kalman J, Kitajka K, Pakaski M, Zvara A, Juhasz A, Vincze G, et al. Gene expression profile analysis of lymphocytes from Alzheimer's patients. Psychiatr Genet. 2005;15:1-6.
[44] Ahn HJ, Chen ZL, Zamolodchikov D, Norris EH, Strickland S. Interactions of beta-amyloid peptide with fibrinogen and coagulation factor XII may contribute to Alzheimer's disease. Curr Opin Hematol. 2017;24:427-31.
[45] Begic E, Hadzidedic S, Obradovic S, Begic Z, Causevic M. Increased Levels of Coagulation Factor XI in Plasma Are Related to Alzheimer's Disease Diagnosis. J Alzheimers Dis. 2020;77:375-86.
[46] Rohmann JL, Longstreth WT, Jr., Cushman M, Fitzpatrick AL, Heckbert SR, Rice K, et al. Coagulation factor VIII, white matter hyperintensities and cognitive function: Results from the Cardiovascular Health Study. PLoS One. 2020;15:e0242062.
[47] Chen S, Chen ST, Sun Y, Xu Z, Wang Y, Yao SY, et al. Fibroblast growth factor 21 ameliorates neurodegeneration in rat and cellular models of Alzheimer's disease. Redox Biol. 2019;22:101133.
[48] Taliyan R, Chandran SK, Kakoty V. Therapeutic Approaches to Alzheimer's Type of Dementia: A Focus on FGF21 Mediated Neuroprotection. Curr Pharm Des. 2019;25:2555-68.
[49] Zhai W, Zhang T, Jin Y, Huang S, Xu M, Pan J. The fibroblast growth factor system in cognitive disorders and dementia. Front Neurosci. 2023;17:1136266.
[50] Cruchaga C, Western D, Timsina J, Wang L, Wang C, Yang C, et al. Proteogenomic analysis of human cerebrospinal fluid identifies neurologically relevant regulation and informs causal proteins for Alzheimer's disease. Res Sq. 2023.
[51] Pietzner M, Wheeler E, Carrasco-Zanini J, Cortes A, Koprulu M, Worheide MA, et al. Mapping the proteo-genomic convergence of human diseases. Science. 2021;374:eabj1541.
[52] Wallace C. A more accurate method for colocalisation analysis allowing for multiple causal variants. PLoS Genet. 2021;17:e1009440.
[53] Narikiyo T, Kitamura K, Adachi M, Miyoshi T, Iwashita K, Shiraishi N, et al. Regulation of prostasin by aldosterone in the kidney. J Clin Invest. 2002;109:401-8.
[54] Wang C, Chao J, Chao L. Adenovirus-mediated human prostasin gene delivery is linked to increased aldosterone production and hypertension in rats. Am J Physiol Regul Integr Comp Physiol. 2003;284:R1031-6.
[55] Zhu H, Guo D, Li K, Yan W, Tan Y, Wang X, et al. Prostasin: a possible candidate gene for human hypertension. Am J Hypertens. 2008;21:1028-33.
[56] Uchimura K, Hayata M, Mizumoto T, Miyasato Y, Kakizoe Y, Morinaga J, et al. The serine protease prostasin regulates hepatic insulin sensitivity by modulating TLR4 signalling. Nat Commun. 2014;5:3428.
[57] Bao X, Xu B, Muhammad IF, Nilsson PM, Nilsson J, Engstrom G. Plasma prostasin: a novel risk marker for incidence of diabetes and cancer mortality. Diabetologia. 2022;65:1642-51.
[58] Stattin K, Lind L, Elmstahl S, Wolk A, Lemming EW, Melhus H, et al. Physical activity is associated with a large number of cardiovascular-specific proteins: Cross-sectional analyses in two independent cohorts. Eur J Prev Cardiol. 2019;26:1865-73.
[59] Chen HH, Petty LE, Sha J, Zhao Y, Kuzma A, Valladares O, et al. Genetically regulated expression in late-onset Alzheimer's disease implicates risk genes within known and novel loci. Transl Psychiatry. 2021;11:618.
[60] Fominykh V, Shadrin AA, Jaholkowski PP, Bahrami S, Athanasiu L, Wightman DP, et al. Shared genetic loci between Alzheimer's disease and multiple sclerosis: Crossroads between neurodegeneration and immune system. Neurobiol Dis. 2023;183:106174.

[61] Salih DA, Bayram S, Guelfi S, Reynolds RH, Shoai M, Ryten M, et al. Genetic variability in response to amyloid beta deposition influences Alzheimer's disease risk. Brain Commun. 2019;1:fcz022.
[62] Rajakumar K, Yan Q, Khalid AT, Feingold E, Vallejo AN, Demirci FY, et al. Gene Expression and Cardiometabolic Phenotypes of Vitamin D-Deficient Overweight and Obese Black Children. Nutrients. 2019;11.
[63] Wei Z, Yang Y, Li Q, Yin Y, Wei Z, Zhang W, et al. The transcriptome of circulating cells indicates potential biomarkers and therapeutic targets in the course of hypertension-related myocardial infarction. Genes Dis. 2021;8:555-68.
[64] Lind L, Zanetti D, Ingelsson M, Gustafsson S, Arnlov J, Assimes TL. Large-Scale Plasma Protein Profiling of Incident Myocardial Infarction, Ischemic Stroke, and Heart Failure. J Am Heart Assoc. 2021;10:e023330.
[65] Abel P, Wussow S, Blucher H, Gros G, Rettig R, Honig A. Erythrocyte carbonic anhydrase activity in smokers and in diabetic patients. Exp Clin Endocrinol Diabetes. 1997;105 Suppl 2:17-9.
[66] Hoff E, Zou D, Schiza S, Demir Y, Grote L, Bouloukaki I, et al. Carbonic anhydrase, obstructive sleep apnea and hypertension: Effects of intervention. J Sleep Res. 2020;29:e12956.
[67] Qian J, Shen Q, Zhang T, Chen J, Chen L, Dong Y, et al. Carbonic anhydrase 14 protects the liver against the cytotoxicity of bile acids in a biliary bicarbonate umbrella-related manner. Life Sci. 2022;310:121117.
[68] Salameh TS, Mortell WG, Logsdon AF, Butterfield DA, Banks WA. Disruption of the hippocampal and hypothalamic blood-brain barrier in a diet-induced obese model of type II diabetes: prevention and treatment by the mitochondrial carbonic anhydrase inhibitor, topiramate. Fluids Barriers CNS. 2019;16:1.
[69] Liu X, Pan S, Xanthakis V, Vasan RS, Psaty BM, Austin TR, et al. Plasma proteomic signature of decline in gait speed and grip strength. Aging Cell. 2022;21:e13736.
[70] Allen JM, Brachvogel B, Farlie PG, Fitzgerald J, Bateman JF. The extracellular matrix protein WARP is a novel component of a distinct subset of basement membranes. Matrix Biol. 2008;27:295-305.
[71] Moller AL, Vasan RS, Levy D, Andersson C, Lin H. Integrated omics analysis of coronary artery calcifications and myocardial infarction: the Framingham Heart Study. Sci Rep. 2023;13:21581.
[72] Hemanthakumar KA, Fang S, Anisimov A, Mayranpaa MI, Mervaala E, Kivela R. Cardiovascular disease risk factors induce mesenchymal features and senescence in mouse cardiac endothelial cells. Elife. 2021;10.
[73] Duong T, Lopez IA, Ishiyama A, Ishiyama G. Immunocytochemical distribution of WARP (von Willebrand A domain-related protein) in the inner ear. Brain Res. 2011;1367:50-61.
[74] Weller AE, Ferraro TN, Doyle GA, Reiner BC, Crist RC, Berrettini WH. Single Nucleus Transcriptome Data from Alzheimer's Disease Mouse Models Yield New Insight into Pathophysiology. J Alzheimers Dis. 2022;90:1233-47.
[75] Bonham LW, Steele NZR, Karch CM, Manzoni C, Geier EG, Wen N, et al. Protein network analysis reveals selectively vulnerable regions and biological processes in FTD. Neurol Genet. 2018;4:e266.
[76] Sinsky J, Majerova P, Kovac A, Kotlyar M, Jurisica I, Hanes J. Physiological Tau Interactome in Brain and Its Link to Tauopathies. J Proteome Res. 2020;19:2429-42.
[77] Tourette C, Li B, Bell R, O'Hare S, Kaltenbach LS, Mooney SD, et al. A large scale Huntingtin protein interaction network implicates Rho GTPase signaling pathways in Huntington disease. J Biol Chem. 2014;289:6709-26.
[78] Schiano C, Balbi C, Burrello J, Ruocco A, Infante T, Fiorito C, et al. De novo DNA methylation induced by circulating extracellular vesicles from acute coronary syndrome patients. Atherosclerosis. 2022;354:41-52.
[79] Colombo G, Gertow K, Marenzi G, Brambilla M, De Metrio M, Tremoli E, et al. Gene expression profiling reveals multiple differences in platelets from patients with stable angina or non-ST elevation acute coronary syndrome. Thrombosis research. 2011;128:161-8.
[80] Yan T, Ding F, Zhao Y. Integrated identification of key genes and pathways in Alzheimer's disease via comprehensive bioinformatical analyses. Hereditas. 2019;156:25.

[81] Ngo D, Benson MD, Long JZ, Chen ZZ, Wang R, Nath AK, et al. Proteomic profiling reveals biomarkers and pathways in type 2 diabetes risk. JCI Insight. 2021;6.
[82] Zaghlool SB, Sharma S, Molnar M, Matías-García PR, Elhadad MA, Waldenberger M, et al. Revealing the role of the human blood plasma proteome in obesity using genetic drivers. Nature communications. 2021;12:1279.
[83] Banerjee S, Prabhu Basrur N, Rai PS. Omics technologies in personalized combination therapy for cardiovascular diseases: challenges and opportunities. Personalized Medicine. 2021;18:595-611.
[84] Shaheen N, Shaheen A, Diab RA, Saad AM, Abdelwahab OA, Soliman S, et al. Association of serum leptin and ghrelin levels with smoking status on body weight: a systematic review and meta-analysis. Front Psychiatry. 2023;14:1296764.
[85] Gunstad J, Spitznagel MB, Keary TA, Glickman E, Alexander T, Karrer J, et al. Serum leptin levels are associated with cognitive function in older adults. Brain Res. 2008;1230:233-6.
[86] Holden KF, Lindquist K, Tylavsky FA, Rosano C, Harris TB, Yaffe K. Serum leptin level and cognition in the elderly: Findings from the Health ABC Study. Neurobiol Aging. 2009;30:1483-9.
[87] Pannacciulli N, Le DS, Chen K, Reiman EM, Krakoff J. Relationships between plasma leptin concentrations and human brain structure: a voxel-based morphometric study. Neurosci Lett. 2007;412:248-53.
[88] Rajagopalan P, Toga AW, Jack CR, Weiner MW, Thompson PM, Alzheimer's Disease Neuroimaging I. Fat-mass-related hormone, plasma leptin, predicts brain volumes in the elderly. Neuroreport. 2013;24:58-62.
[89] Sanborn V, Preis SR, Ang A, Devine S, Mez J, DeCarli C, et al. Association Between Leptin, Cognition, and Structural Brain Measures Among "Early" Middle-Aged Adults: Results from the Framingham Heart Study Third Generation Cohort. J Alzheimers Dis. 2020;77:1279-89.
[90] Lendeckel U, Arndt M, Wrenger S, Nepple K, Huth C, Ansorge S, et al. Expression and activity of ectopeptidases in fibrillating human atria. J Mol Cell Cardiol. 2001;33:1273-81.
[91] Cheng N, Wang H, Zhang W, Wang H, Jin X, Ma X, et al. Comparative Proteomic Investigation of Plasma Reveals Novel Potential Biomarker Groups for Acute Aortic Dissection. Dis Markers. 2020;2020:4785068.
[92] Fang YC, Chan L, Liou JP, Tu YK, Lai MJ, Chen CI, et al. HDAC inhibitor protects chronic cerebral hypoperfusion and oxygen-glucose deprivation injuries via H3K14 and H4K5 acetylation-mediated BDNF expression. J Cell Mol Med. 2020;24:6966-77.
[93] Kim H, Jung J, Park S, Joo Y, Lee S, Sim J, et al. Exercise-Induced Fibroblast Growth Factor-21: A Systematic Review and Meta-Analysis. Int J Mol Sci. 2023;24.
[94] Richter MM, Thomsen MN, Skytte MJ, Kjeldsen SAS, Samkani A, Frystyk J, et al. Effect of a 6-Week Carbohydrate-Reduced High-Protein Diet on Levels of FGF21 and GDF15 in People With Type 2 Diabetes. J Endocr Soc. 2024;8:bvae008.
[95] Platek T, Polus A, Goralska J, Razny U, Dziewonska A, Micek A, et al. Epigenetic Regulation of Processes Related to High Level of Fibroblast Growth Factor 21 in Obese Subjects. Genes (Basel). 2021;12.
[96] van Baak MA, Vink RG, Roumans NJT, Cheng CC, Adams AC, Mariman ECM. Adipose tissue contribution to plasma fibroblast growth factor 21 and fibroblast activation protein in obesity. Int J Obes (Lond). 2020;44:544-7.
[97] Chuang GT, Liu PH, Chyan TW, Huang CH, Huang YY, Lin CH, et al. Genome-wide association study for circulating fibroblast growth factor 21 and 23. Sci Rep. 2020;10:14578.
[98] Wang YS, Ye J, Cao YH, Zhang R, Liu Y, Zhang SW, et al. Increased serum/plasma fibroblast growth factor 21 in type 2 diabetes mellitus: a systematic review and meta-analysis. Postgrad Med J. 2019;95:134-9.
[99] Yang M, Liu C, Jiang N, Liu Y, Luo S, Li C, et al. Fibroblast growth factor 21 in metabolic syndrome. Front Endocrinol (Lausanne). 2023;14:1220426.

[100] Chen LM, Nergard JC, Ni L, Rosser CJ, Chai KX. Long-term exposure to cigarette smoke extract induces hypomethylation at the RUNX3 and IGF2-H19 loci in immortalized human urothelial cells. PLoS One. 2013;8:e65513.
[101] Ishii T, Miyasato Y, Ichijo M, Uchimura K, Furuya F. Membrane protease prostasin promotes insulin secretion by regulating the epidermal growth factor receptor pathway. Sci Rep. 2023;13:9086.
[102] Casselbrant A, Fedorowski A, Frantz S, Engstrom G, Wollmer P, Hamrefors V. Common physiologic and proteomic biomarkers in pulmonary and coronary artery disease. PLoS One. 2022;17:e0264376.
[103] Ohno H, Yamashita K, Doi R, Yamamura K, Kondo T, Taniguchi N. Exercise-induced changes in blood zinc and related proteins in humans. J Appl Physiol (1985). 1985;58:1453-8.
[104] De Simone G, Di Fiore A, Supuran CT. Are carbonic anhydrase inhibitors suitable for obtaining antiobesity drugs? Curr Pharm Des. 2008;14:655-60.
[105] Pang Y, Kartsonaki C, Lv J, Fairhurst-Hunter Z, Millwood IY, Yu C, et al. Associations of Adiposity, Circulating Protein Biomarkers, and Risk of Major Vascular Diseases. JAMA Cardiol. 2021;6:276-86.
[106] Thongboonkerd V, Chutipongtanate S, Kanlaya R, Songtawee N, Sinchaikul S, Parichatikanond P, et al. Proteomic identification of alterations in metabolic enzymes and signaling proteins in hypokalemic nephropathy. Proteomics. 2006;6:2273-85.
[107] Anzovino A, Canepa E, Alves M, Lemon NL, Carare RO, Fossati S. Amyloid Beta Oligomers Activate Death Receptors and Mitochondria-Mediated Apoptotic Pathways in Cerebral Vascular Smooth Muscle Cells; Protective Effects of Carbonic Anhydrase Inhibitors. Cells. 2023;12.
[108] Carey A, Fossati S. Hypertension and hyperhomocysteinemia as modifiable risk factors for Alzheimer's disease and dementia: New evidence, potential therapeutic strategies, and biomarkers. Alzheimers Dement. 2023;19:671-95.
[109] Provensi G, Carta F, Nocentini A, Supuran CT, Casamenti F, Passani MB, et al. A New Kid on the Block? Carbonic Anhydrases as Possible New Targets in Alzheimer's Disease. Int J Mol Sci. 2019;20.
[110] Shafi O. Inverse relationship between Alzheimer's disease and cancer, and other factors contributing to Alzheimer's disease: a systematic review. BMC Neurol. 2016;16:236.
[111] Fujikawa-Adachi K, Nishimori I, Taguchi T, Onishi S. Human carbonic anhydrase XIV (CA14): cDNA cloning, mRNA expression, and mapping to chromosome 1. Genomics. 1999;61:74-81.
[112] Fitzgerald J, Tay Ting S, Bateman JF. WARP is a new member of the von Willebrand factor A-domain superfamily of extracellular matrix proteins. FEBS Lett. 2002;517:61-6.
[113] Lakshman Kumar P, Wilson AC, Rocco A, Cho MH, Wan E, Hobbs BD, et al. Genetic variation in genes regulating skeletal muscle regeneration and tissue remodelling associated with weight loss in chronic obstructive pulmonary disease. J Cachexia Sarcopenia Muscle. 2021;12:1803-17.
[114] Daily JW, Park S. Association of Plant-Based and High-Protein Diets with a Lower Obesity Risk Defined by Fat Mass in Middle-Aged and Elderly Persons with a High Genetic Risk of Obesity. Nutrients. 2023;15.
[115] Minchenko DO, Kharkova AP, Hubenia OV, Minchenko OH. Insulin receptor, IRS1, IRS2, INSIG1, INSIG2, RRAD, and BAIAP2 gene expressions in glioma U87 cells with ERN1 loss of function: effect of hypoxia and glutamine or glucose deprivation. Endocr Regul. 2013;47:15-26.
[116] Zaghlool SB, Sharma S, Molnar M, Matias-Garcia PR, Elhadad MA, Waldenberger M, et al. Revealing the role of the human blood plasma proteome in obesity using genetic drivers. Nat Commun. 2021;12:1279.
