# Supplementary material for: Plasma proteomic biomarkers as mediators or moderators for the association between poor cardiovascular health and white matter microstructural integrity: The UK Biobank study
Source: Alzheimers Dement. 2025 Jan 17;21(2):e14507. doi: 10.1002/alz.14507 (PMC11864230; doi:10.1002/alz.14507)
Supplement: Supplementary file 2 — Supporting information [file ALZ-21-e14507-s002.pdf]

Supplementary Figure 1. Participant Flowchart

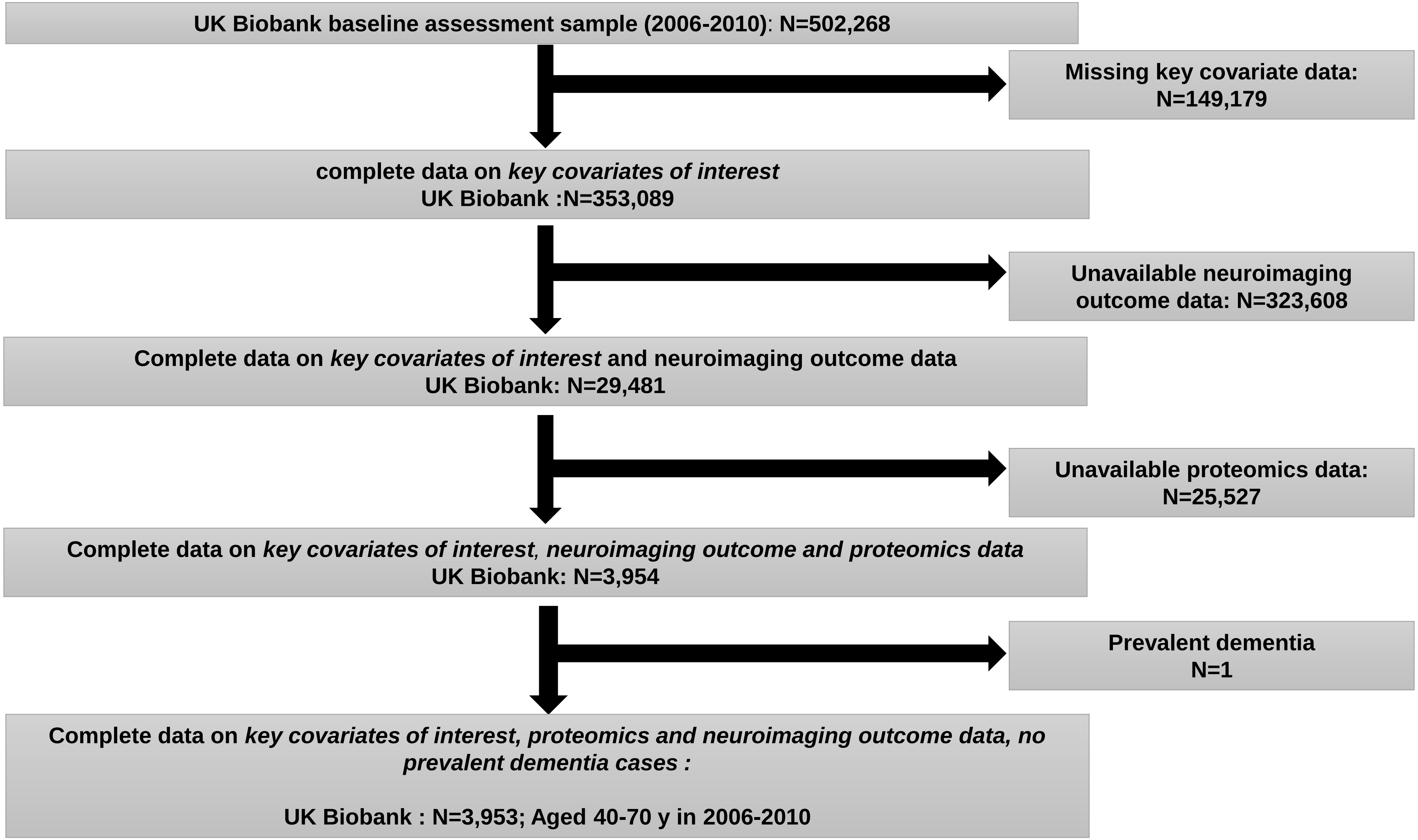

Abbreviations: dMRI=Diffusion-weighted magnetic resonance imaging; UK=United Kingdom
