# Supplementary material for: Plasma proteomic biomarkers as mediators or moderators for the association between poor cardiovascular health and white matter microstructural integrity: The UK Biobank study
Source: Alzheimers Dement. 2025 Jan 17;21(2):e14507. doi: 10.1002/alz.14507 (PMC11864230; doi:10.1002/alz.14507)
Supplement: Supplementary file 6 — Supporting information [file ALZ-21-e14507-s012.pdf]

FIGURE S2. Results of principal components analysis for top consistent mediators (k=10), overall (N_{max}=3,581: UK biobank 2006-2021

(A) Scree plot

(B) Principal Component loading plot (PC1, PC2 and PC3)

(A)

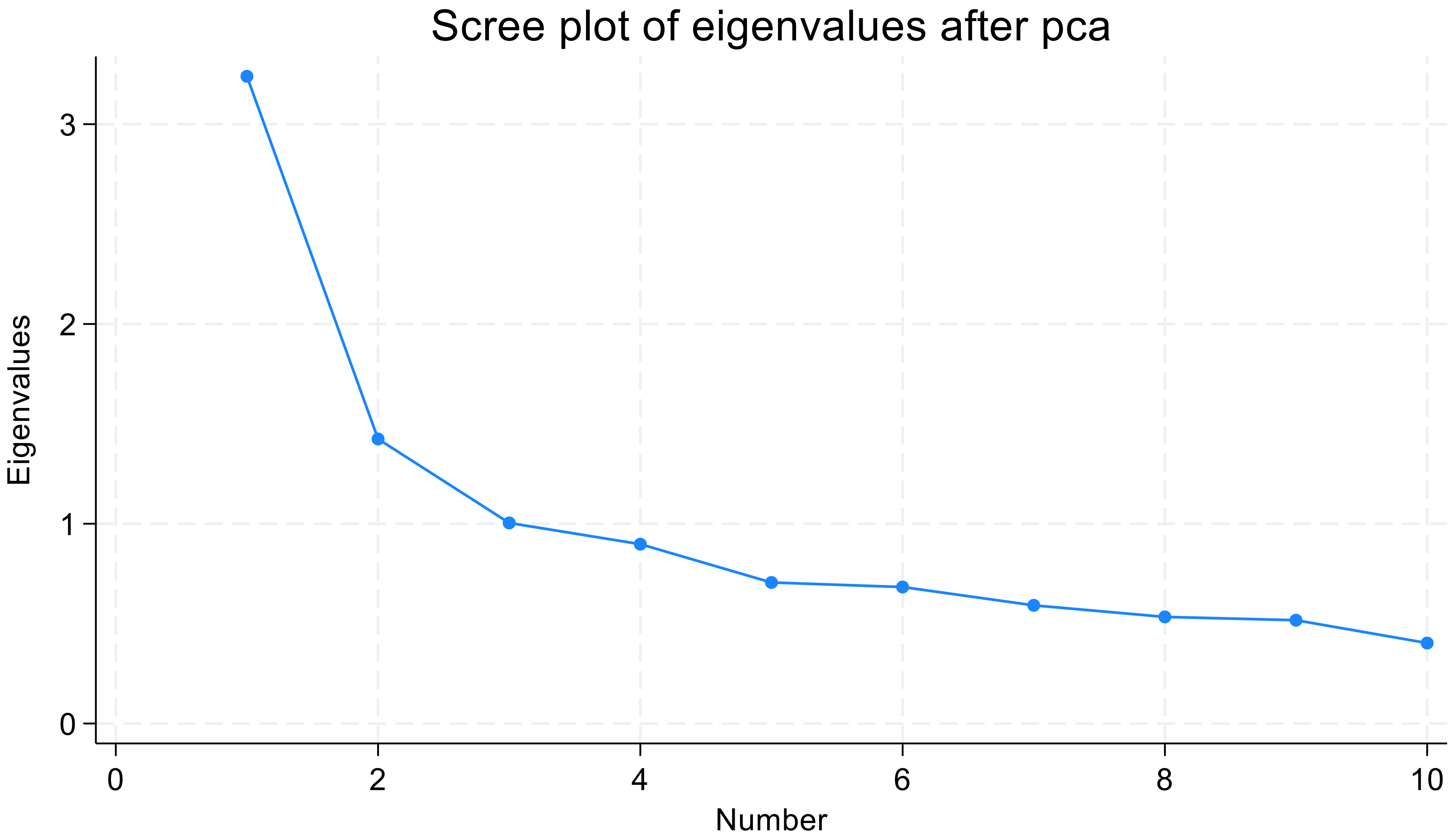
Scree plot of eigenvalues after pca
Eigenvalues
0
1
2
3
0
2
4
6
8
10
Number

(B)

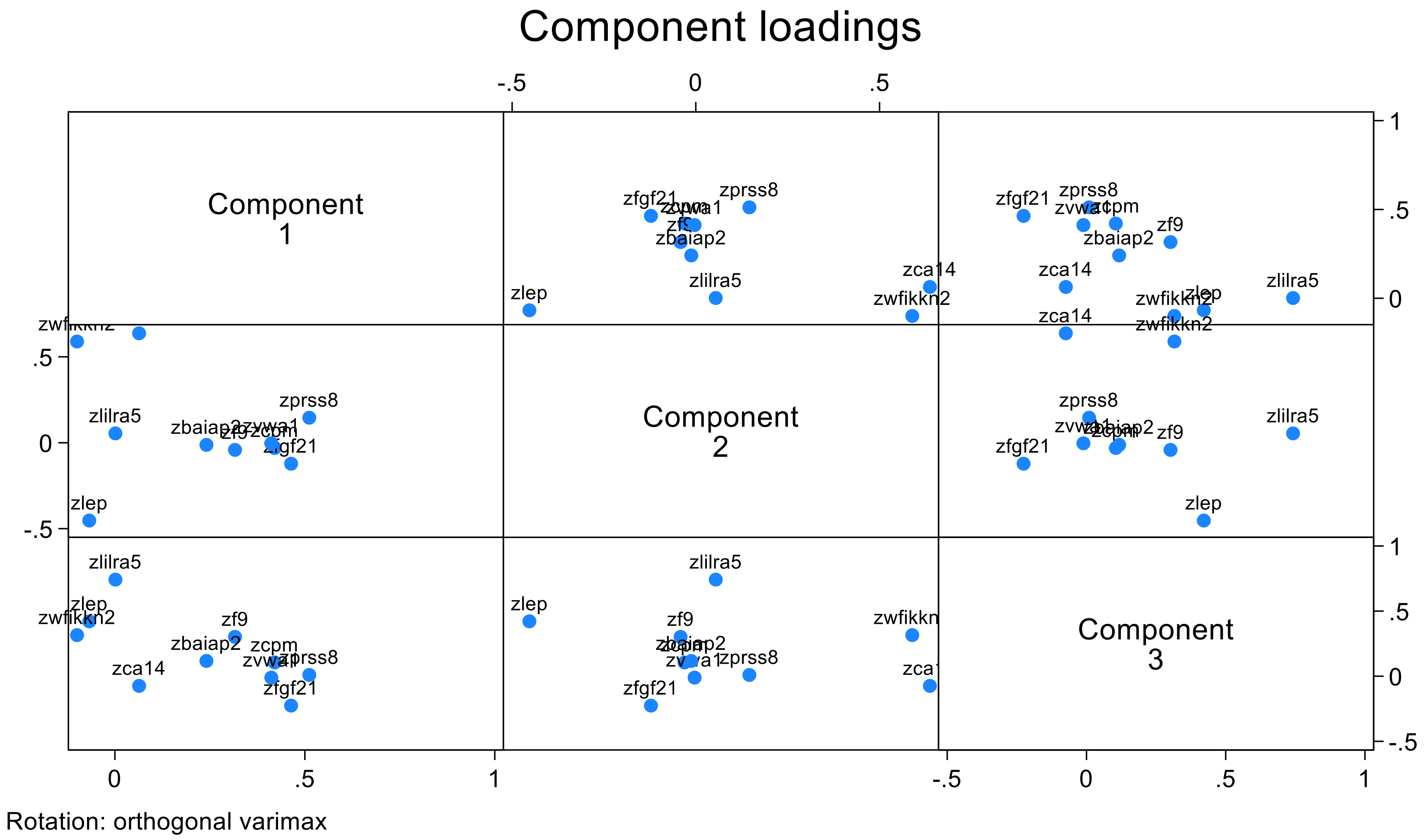
Component loadings
Component 1
Component 2
Component 3
zfgf21
zprss8
zvwa1
zcpm
zf9
zbaiap2
zca14
zlilra5
zlep
zwfikkn2
Rotation: orthogonal varimax
