# Supplementary material for: Plasma proteomic biomarkers as mediators or moderators for the association between poor cardiovascular health and white matter microstructural integrity: The UK Biobank study
Source: Alzheimers Dement. 2025 Jan 17;21(2):e14507. doi: 10.1002/alz.14507 (PMC11864230; doi:10.1002/alz.14507)
Supplement: Supplementary file 7 — Supporting information [file ALZ-21-e14507-s015.pdf]

FIGURE S3. Four-way decomposition of the association between Poor Cardiovascular Health as measured by Life's Essential 8 total score (z-scored, reverse coded, LE8z_rev) and FA_{mean} (along with OD_{mean}) by principal component score of selected plasma proteomic biomarkers with consistent mediation (k=10, 3 PCA scores), overall (N=3,581): UK biobank 2006-2021

Abbreviations: ereri_cde= excess relative risk due to neither mediation nor interaction or controlled direct effect; ereri_intmed= excess relative risk due to mediated interaction or mediated interaction; ereri_intref= excess relative risk due to interaction only or interaction referent; ereri_pie= excess relative risk due to mediation only or pure indirect effect; PC1=First principal component; PC2=Second principal component; p_cde=proportion of total effect that is controlled direct effect; p_intmed=proportion of total effect that is mediated interaction; p_intref=proportion of total effect that is interaction referent; p_pie=proportion of total effect that is pure indirect effect; tereri= Total excess relative risk; UK=United Kingdom. See **supplementary Table 3** for protein abbreviations and **supplementary Table 2** (**Appendix II**) for results of PCA. Other Protein abbreviations are found at https://www.ncbi.nlm.nih.gov/gene/.

(A) FA_{mean} vs. PC1

(B) FA_{mean} vs. PC2

(C) FA_{mean} vs. PC3

(D) OD_{mean} vs. PC1

(E) OD_{mean} vs. PC2

(F) OD_{mean} vs. PC3

(A) FAmean vs. PC1

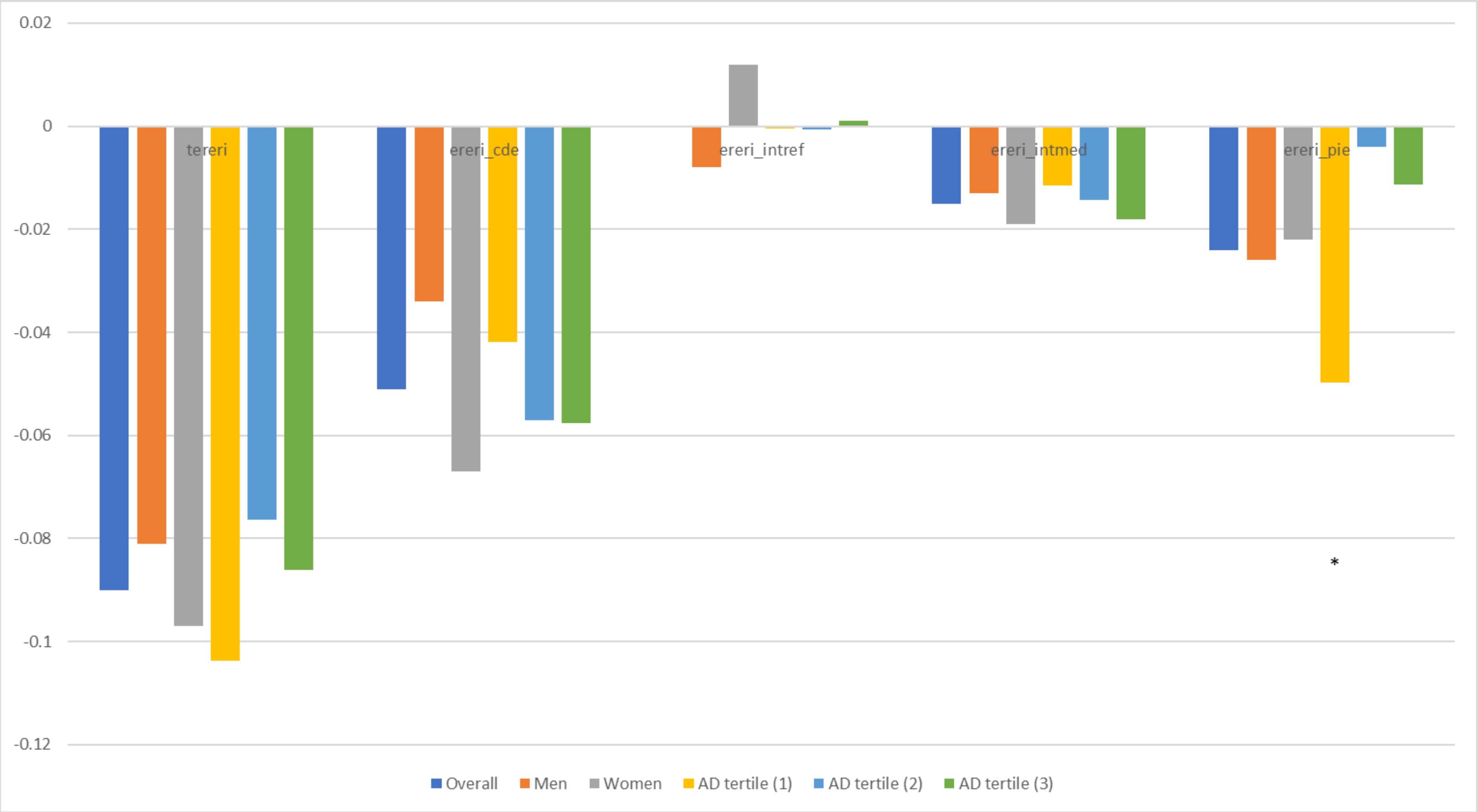

	Overall	Men	Women	AD tertile (1)	AD tertile (2)	AD tertile (3)
tereri	**-0.090**	**-0.081**	**-0.097**	**-0.1037**	**-0.0763**	**-0.0861**
ereri_cde	-0.051	-0.034	-0.067	-0.0418	**-0.0571**	-0.0577
ereri_intref	-0.0001	-0.008	0.012	-0.0004	-0.0007	0.0011
ereri_intmed	-0.015	-0.013	-0.019	-0.0116	-0.0143	-0.0181
ereri_pie	**-0.024**	**-0.026**	**-0.022**	**-0.0498**	**-0.0041**	**-0.0113**

Bolded numbers are associated with $p<0.05$

(B) FAmean vs. PC2

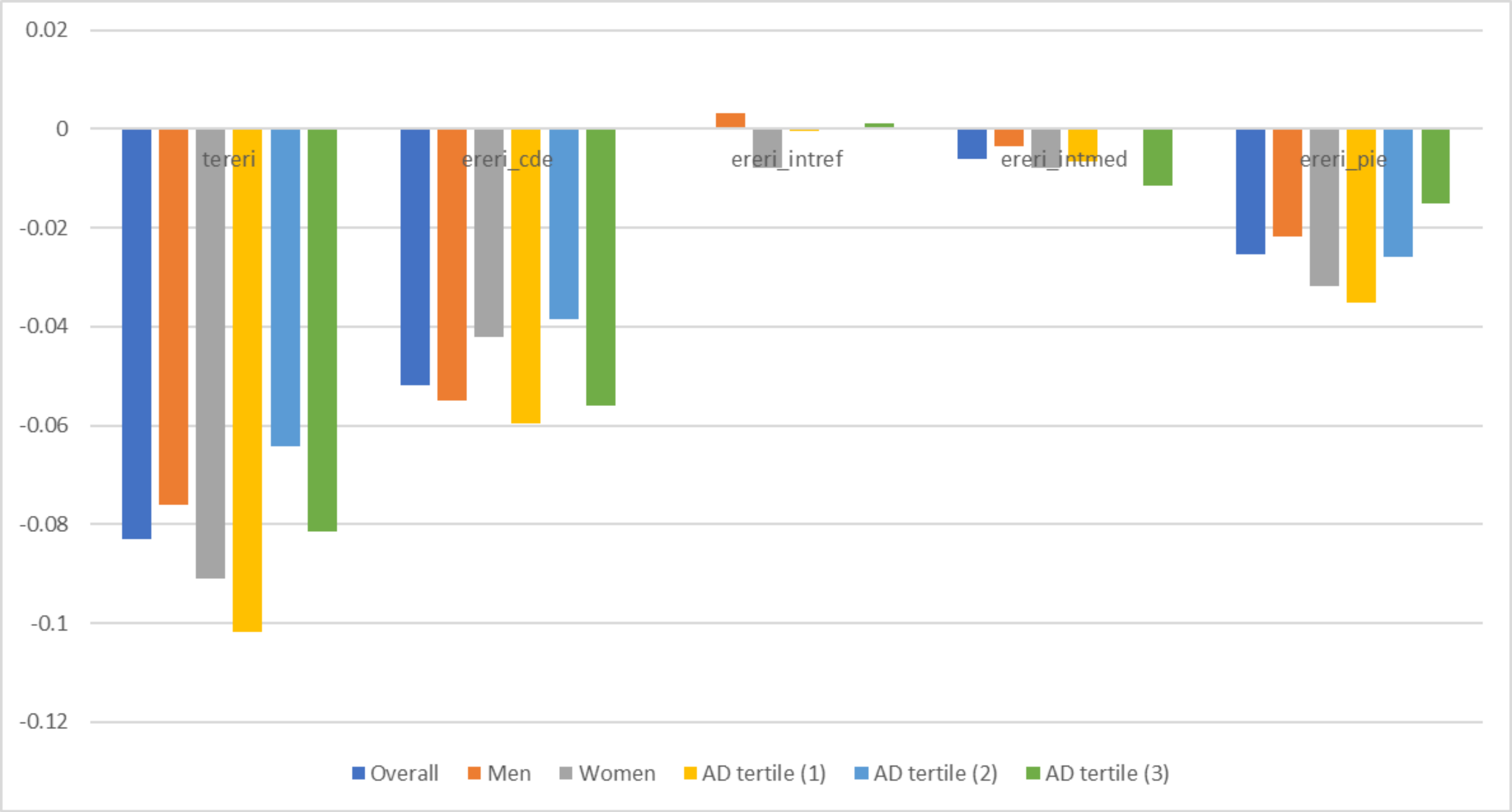

	Overall	Men	Women	AD tertile (1)	AD tertile (2)	AD tertile (3)
tereri	**-0.083**	**-0.076**	**-0.091**	**-0.1018**	**-0.0642**	**-0.0816**
ereri_cde	**-0.052**	-0.055	-0.042	-0.0596	-0.0384	-0.0560
ereri_intref	0.00006	0.0033	-0.008	-0.0004	0.00000	0.00100
ereri_intmed	-0.0060	-0.0036	-0.008	-0.0065	0.00000	-0.0115
ereri_pie	**-0.0255**	**-0.0217**	**-0.0319**	**-0.0352**	**-0.0258**	-0.01509

Bolded numbers are associated with $p<0.05$

(C) FAmean vs. PC3

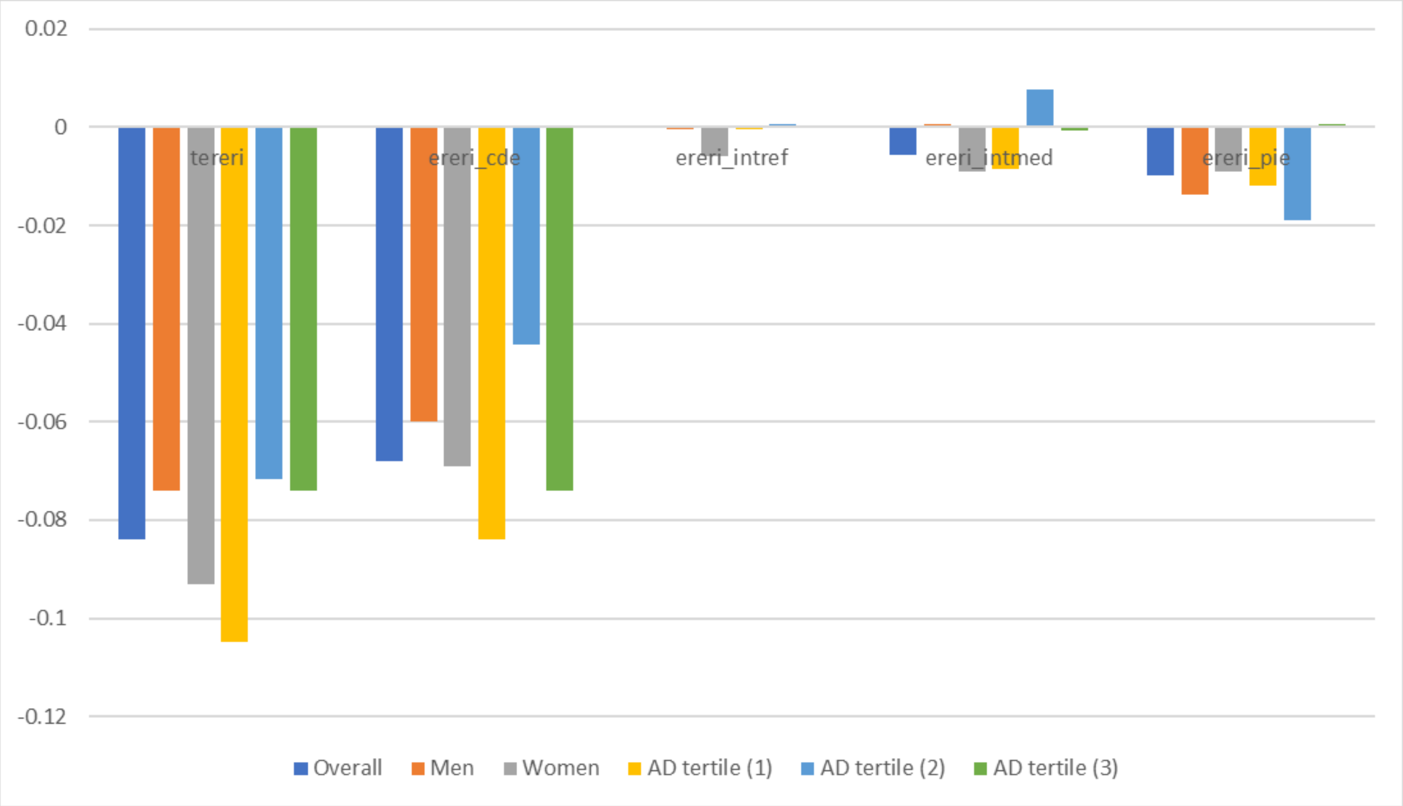

	Overall	Men	Women	AD tertile (1)	AD tertile (2)	AD tertile (3)
tereri	**-0.084**	**-0.074**	**-0.093**	**-0.1047**	**-0.0717**	**-0.0740**
ereri_cde	**-0.068**	**-0.060**	**-0.069**	**-0.0840**	-0.0442	**-0.0740**
ereri_intref	0.000056	-0.0004	-0.006	-0.0004	0.0007	0.00006
ereri_intmed	-0.00577	0.0006	-0.009	-0.0086	0.0077	-0.00062
ereri_pie	-0.0099	-0.0137	-0.009	-0.0118	-0.019	0.00048

Bolded numbers are associated with $p<0.05$

(D) ODmean vs. PC1

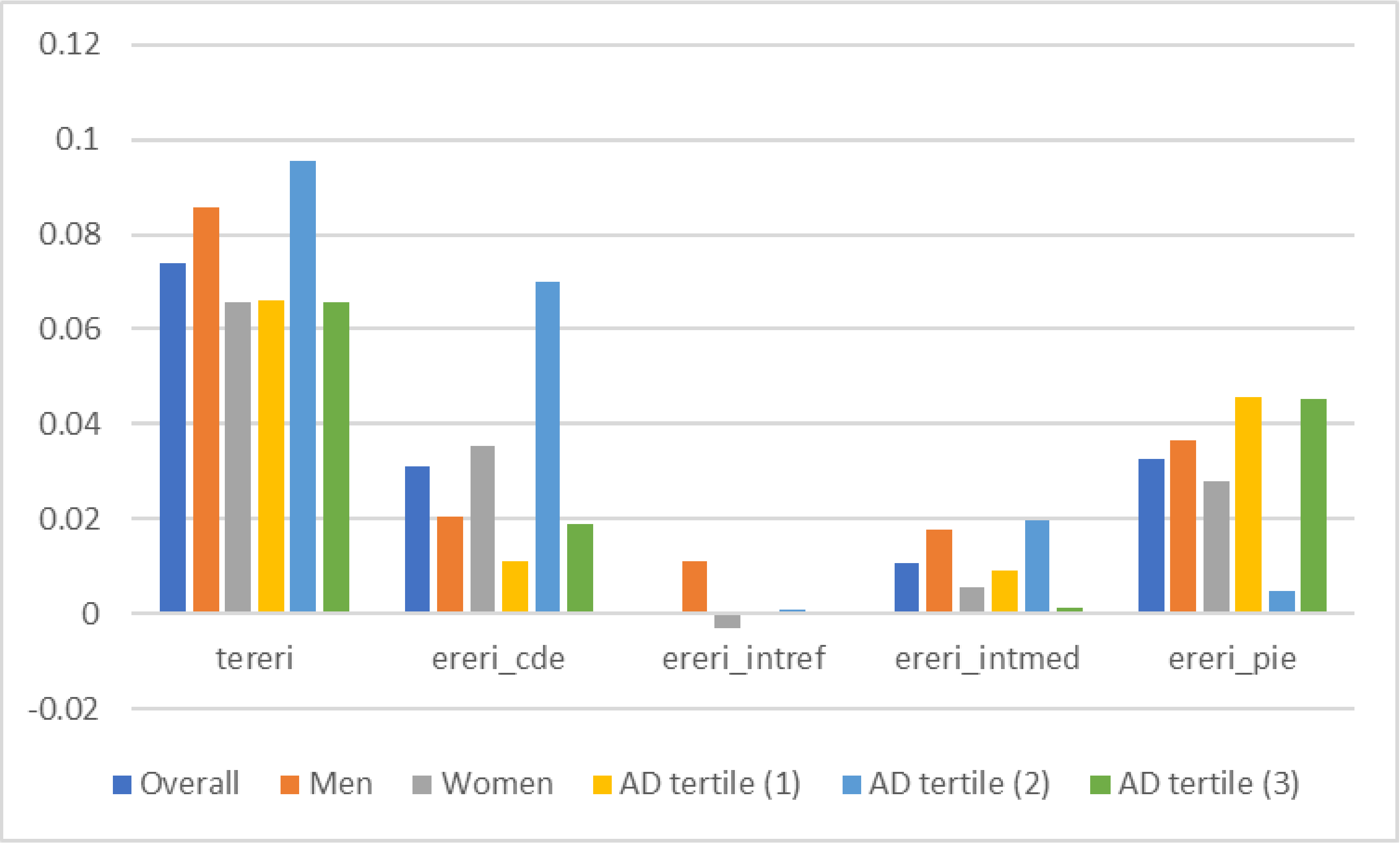

	Overall	Men	Women	AD tertile (1)	AD tertile (2)	AD tertile (3)
tereri	**0.074**	**0.0856**	**0.0657**	0.0661	**0.0957**	**0.0658**
ereri_cde	0.031	0.0204	0.0354	0.0110	**0.0701**	0.0190
ereri_intref	0.0001	**0.0110**	-0.0032	0.0003	0.00097	-0.0001
ereri_intmed	0.0106	**0.0178**	0.0054	0.0091	**0.0198**	0.0014
ereri_pie	**0.0327**	**0.0367**	**0.0281**	**0.0457**	0.0048	**0.0454**

Bolded numbers are associated with $p<0.05$

(E) ODmean vs. PC2

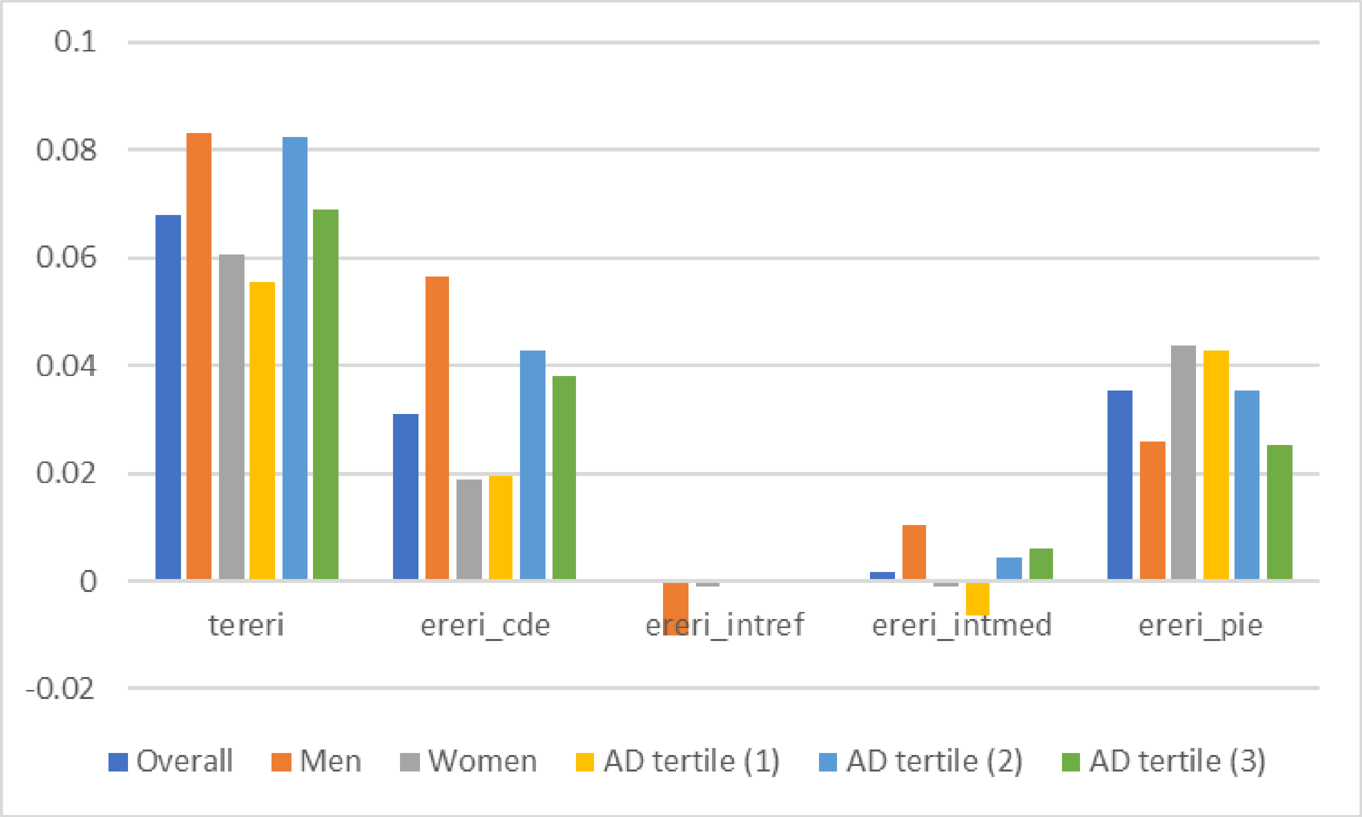

	Overall	Men	Women	AD tertile (1)	AD tertile (2)	AD tertile (3)
tereri	**0.068**	**0.0830**	**0.0606**	0.0557	**0.0826**	**0.0690**
ereri_cde	**0.031**	**0.0566**	0.0190	0.0196	0.0428	0.0382
ereri_intref	0.0000	-0.010	-0.0009	-0.0004	0.0002	-0.0005
ereri_intmed	0.0017	0.0104	-0.001	-0.0065	0.0043	0.0060
ereri_pie	**0.0353**	**0.0258**	**0.0436**	**0.0429**	**0.0352**	**0.0253**

Bolded numbers are associated with $p<0.05$

(F) ODmean vs. PC3

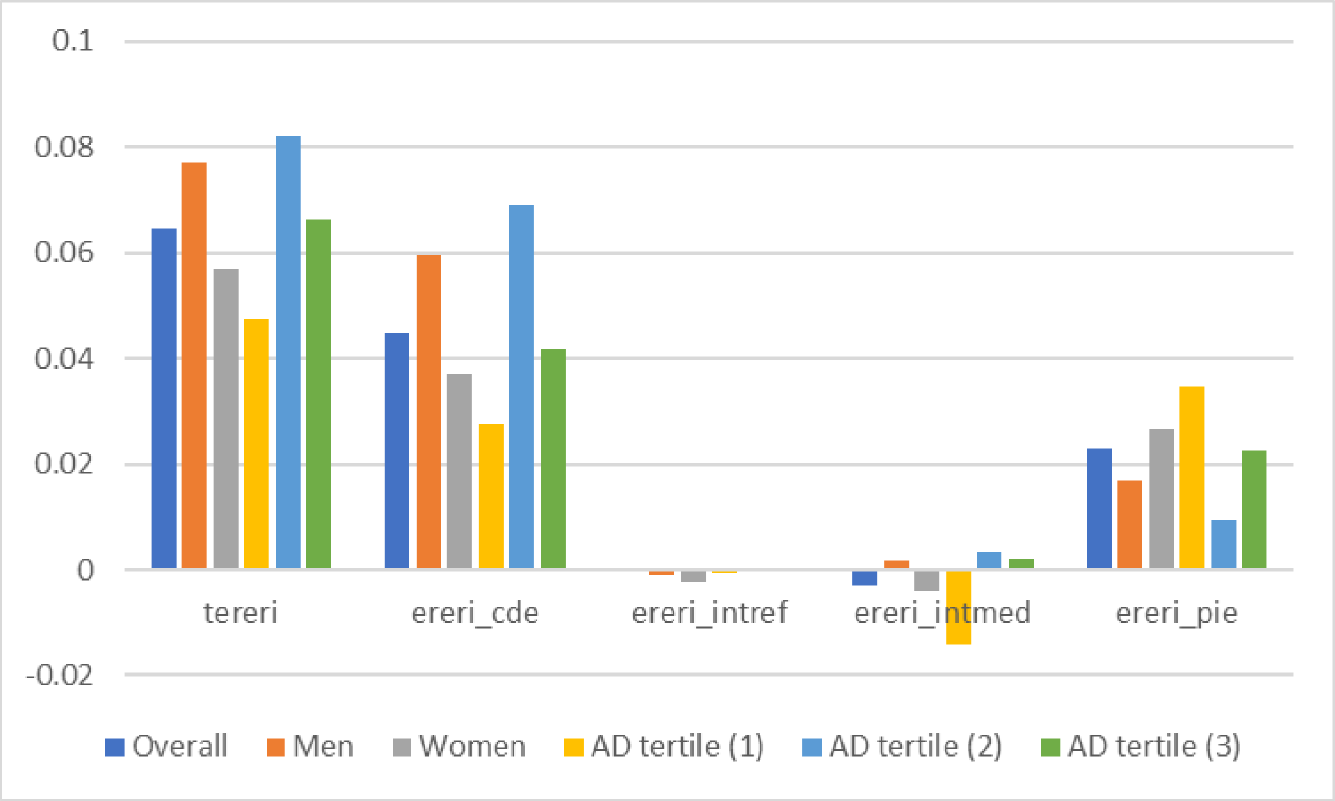

	Overall	Men	Women	AD tertile (1)	AD tertile (2)	AD tertile (3)
tereri	**0.0646**	**0.0770**	**0.0570**	0.0473	**0.0822**	**0.0663**
ereri_cde	**0.0447**	**0.0596**	0.0370	0.0275	**0.0691**	0.0418
ereri_intref	-0.0000	-0.001	-0.0025	-0.0007	0.0003	-0.0002
ereri_intmed	-0.0029	0.0016	-0.0041	-0.0142	0.0034	0.0020
ereri_pie	**0.0228**	**0.0168**	**0.0266**	**0.0348**	0.0093	**0.0227**

Bolded numbers are associated with $p<0.05$
