# Supplementary material for: Plasma proteomic biomarkers as mediators or moderators for the association between poor cardiovascular health and white matter microstructural integrity: The UK Biobank study
Source: Alzheimers Dement. 2025 Jan 17;21(2):e14507. doi: 10.1002/alz.14507 (PMC11864230; doi:10.1002/alz.14507)
Supplement: Supplementary file 8 — Supporting information [file ALZ-21-e14507-s007.pdf]

FIGURE S4.

OLINK Insight Pathway browser findings for all plasma proteomic biomarkers found to be significant mediators (PIE, $p<0.05$) and STRING analysis (k=212 proteins for FA_{mean}; k=142 for OD_{meam}), k=313 for both.

(A)-(C) OLINK Insight pathways: distinct and shared between FA_{mean} (p=818) and OD_{mean} (p=522); both combined (p=1002)

(D) String analysis for FA_{mean}

(E) String analysis for OD_{mean}

Sources: https://insight.olink.com/. The full list of plasma protein biomarkers included into the insight pathway analysis is found on github: https://github.com/baydounm/UKB-paper13-supplementarydata. The same list was included in the STRING analysis: https://string-db.org

Abbreviations: p=Number of OLINK Insight pathways; PIE=Pure indirect effect. Protein abbreviations are found at https://www.ncbi.nlm.nih.gov/gene/.

(A)

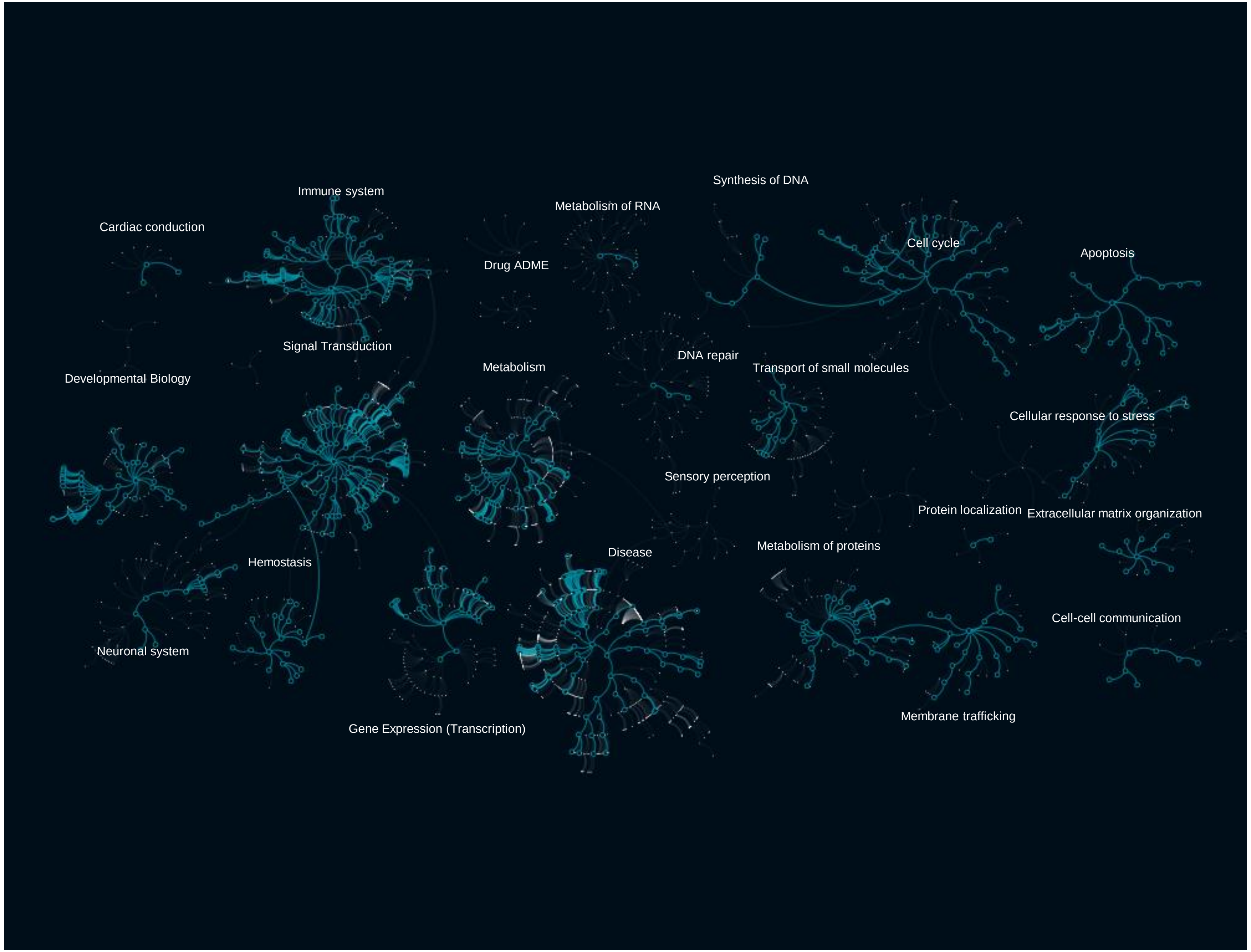
Synthesis of DNA
Immune system
Metabolism of RNA
Cardiac conduction
Cell cycle
Apoptosis
Drug ADME
Signal Transduction
DNA repair
Metabolism
Transport of small molecules
Developmental Biology
Cellular response to stress
Sensory perception
Protein localization
Extracellular matrix organization
Metabolism of proteins
Disease
Hemostasis
Cell-cell communication
Neuronal system
Membrane trafficking
Gene Expression (Transcription)

(B)

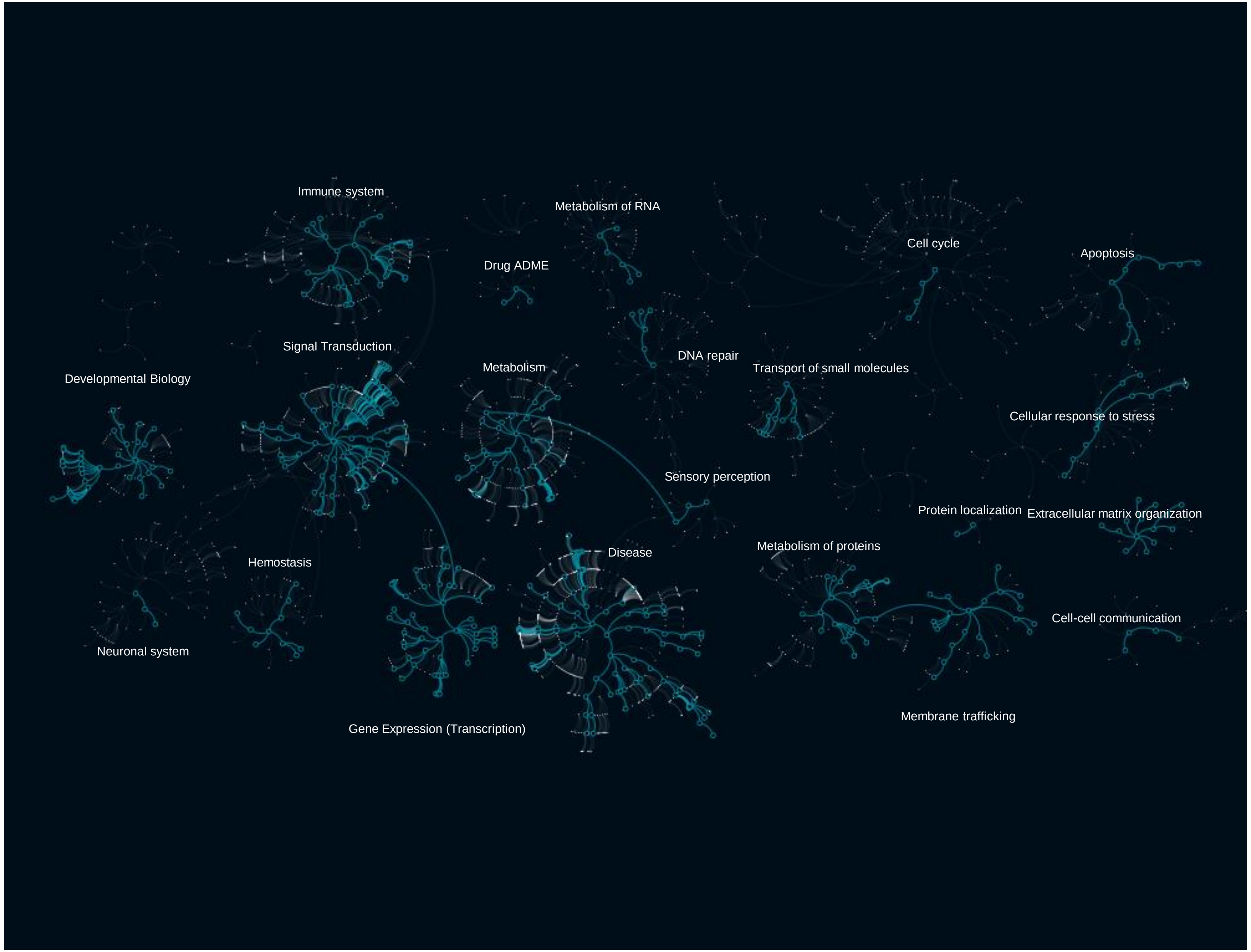
Immune system
Metabolism of RNA
Cell cycle
Apoptosis
Drug ADME
Signal Transduction
DNA repair
Metabolism
Transport of small molecules
Developmental Biology
Cellular response to stress
Sensory perception
Protein localization
Extracellular matrix organization
Metabolism of proteins
Disease
Hemostasis
Cell-cell communication
Neuronal system
Membrane trafficking
Gene Expression (Transcription)

(C)

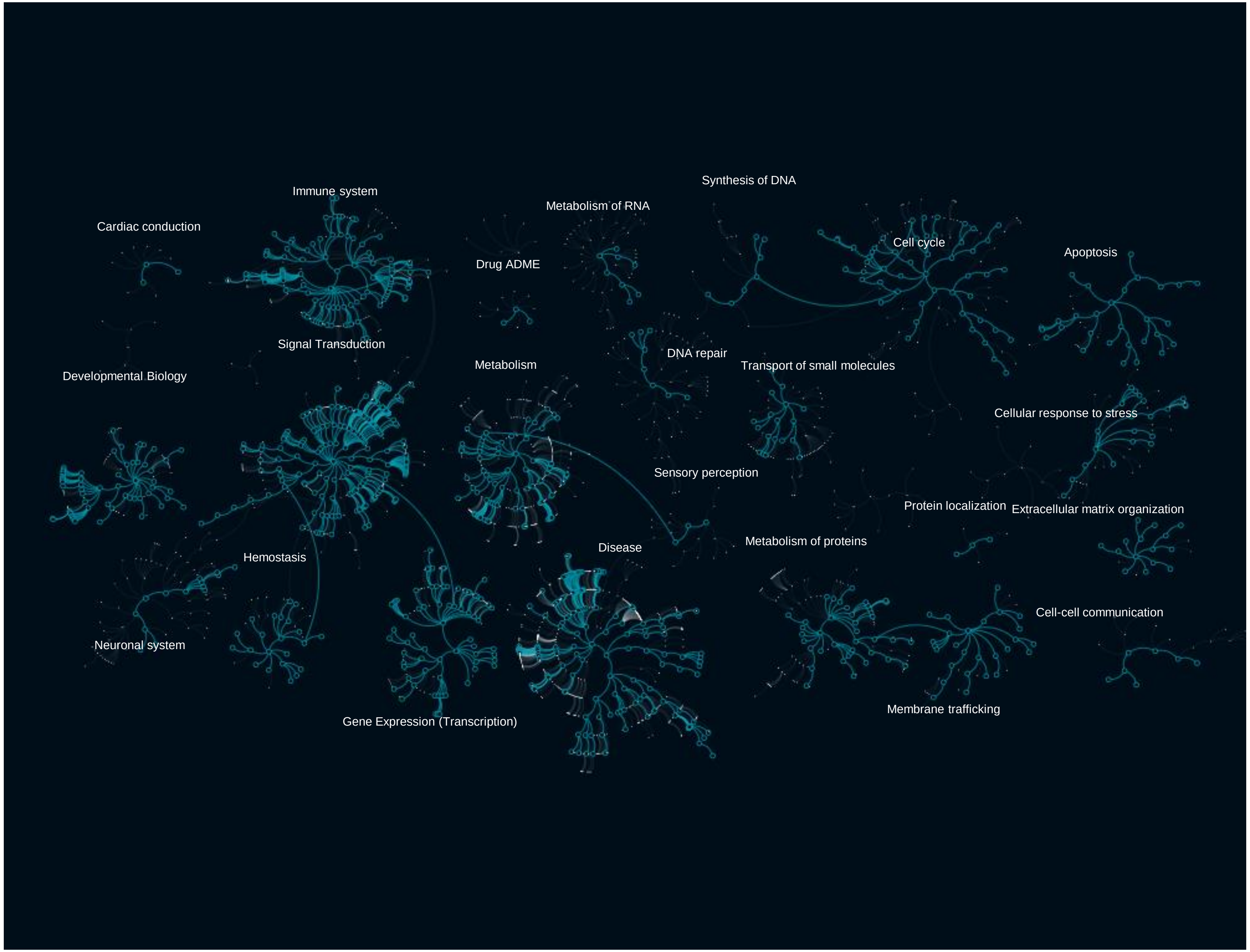
Synthesis of DNA
Immune system
Metabolism of RNA
Cardiac conduction
Cell cycle
Apoptosis
Drug ADME
Signal Transduction
DNA repair
Metabolism
Transport of small molecules
Developmental Biology
Cellular response to stress
Sensory perception
Protein localization
Extracellular matrix organization
Metabolism of proteins
Disease
Hemostasis
Cell-cell communication
Neuronal system
Membrane trafficking
Gene Expression (Transcription)

(D)

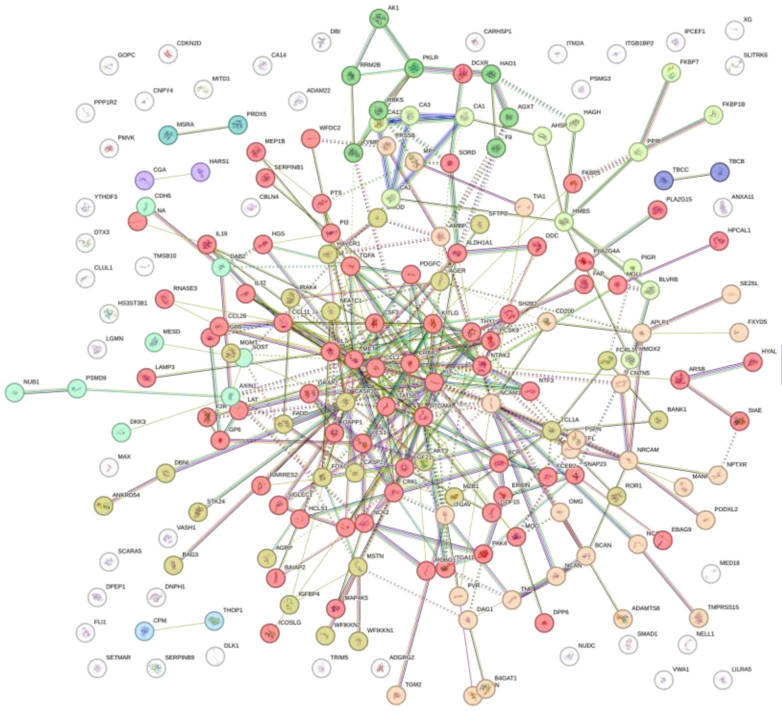

color	cluster Id	gene count	description
	Cluster 1	70	ALDH1A1, ARSB, BAIAP2, BCR, CCL11, CCL2, CCL26, CD93, CRKL, CSF3, DAPP1, DC...
	Cluster 2	29	ADAMTS8, AMBP, APLP1, B4GAT1, BCAN, CD200, CDON, CNTN5, DAG1, FXYD5, ITG...
	Cluster 3	26	AGER, AGRP, AKT3, ANKRD54, BAG3, BANK1, CASP2, CASP3, DBNL, FADD, FCRL1, F...
	Cluster 4	13	+ Nitrogen metabolism
	Cluster 5	8	+ Glyoxylate and dicarboxylate metabolism
	Cluster 6	8	Regulation of aldosterone biosynthesis
	Cluster 7	2	MSRA, PRDX5
	Cluster 8	2	CPM, THOP1
	Cluster 9	2	Post-chaperonin tubulin folding pathway
	Cluster 10	2	CGA, HARS1

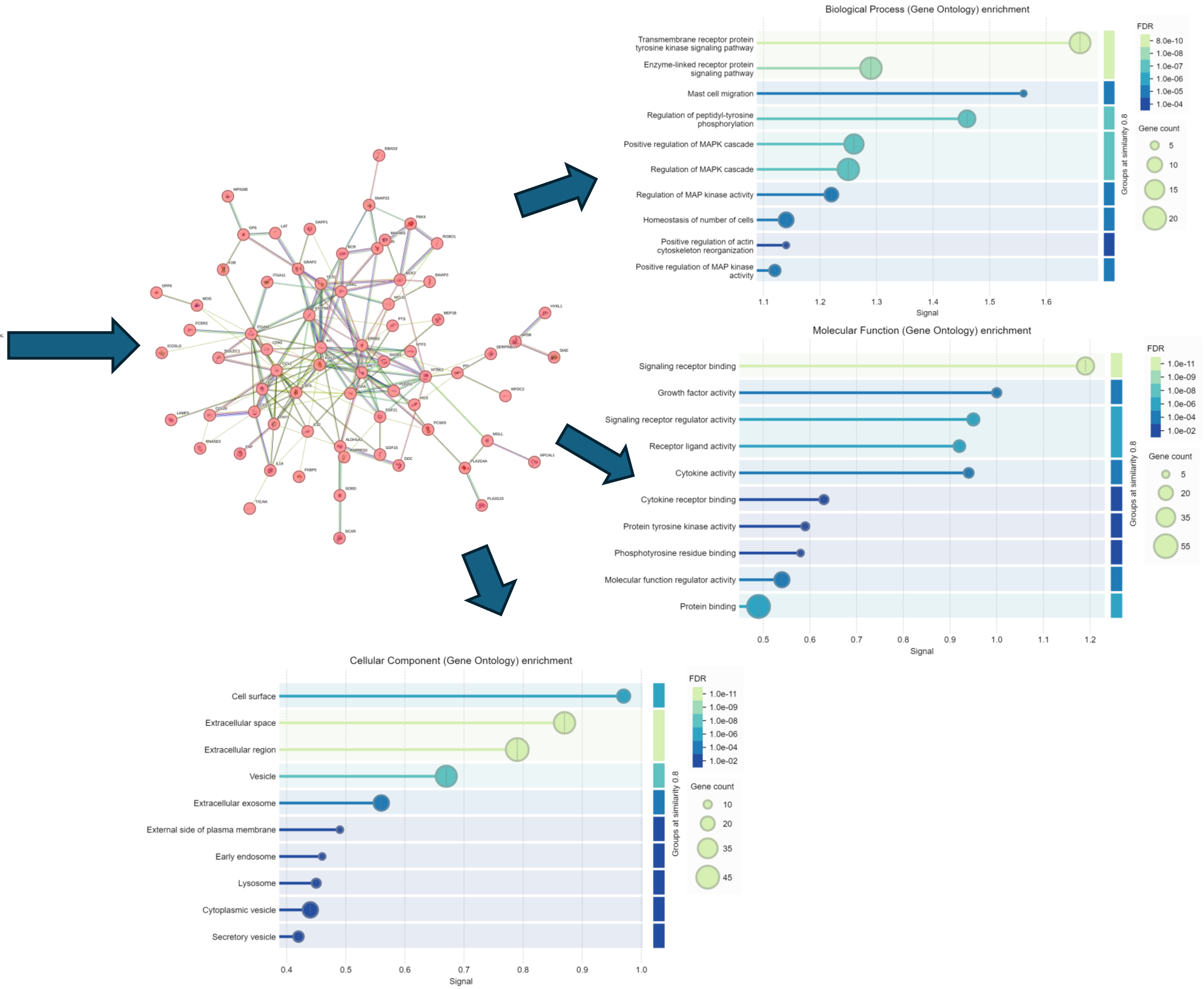

(E)

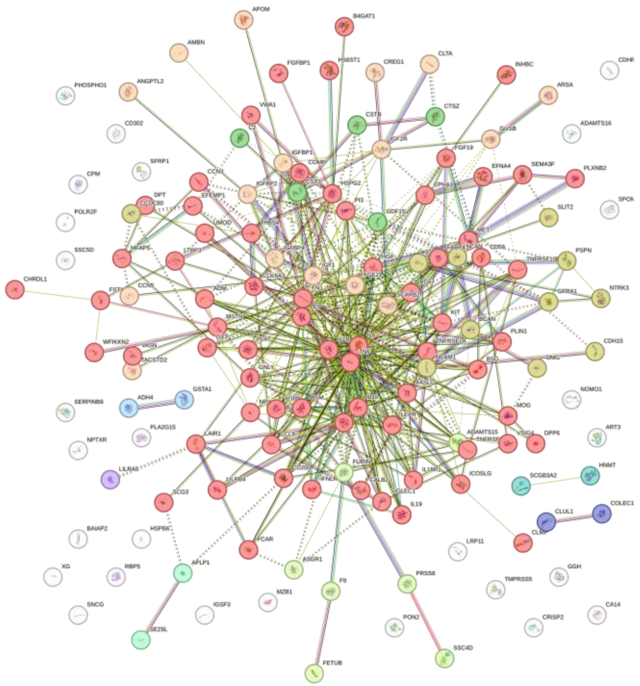

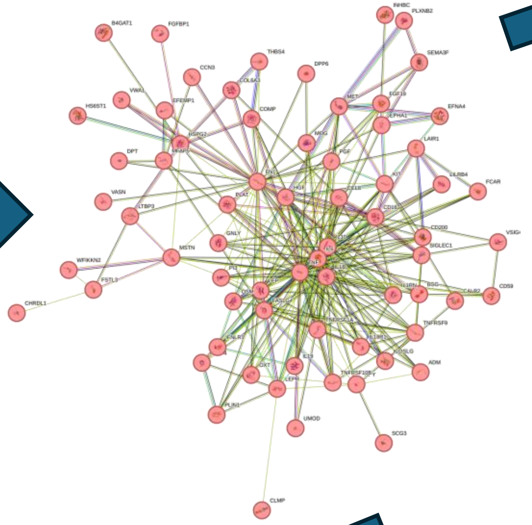

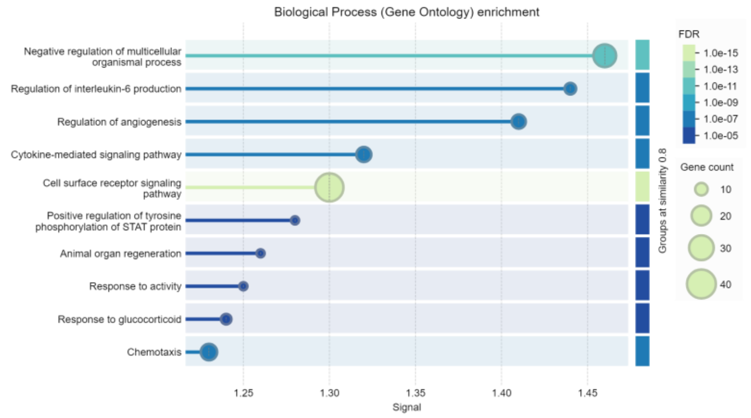

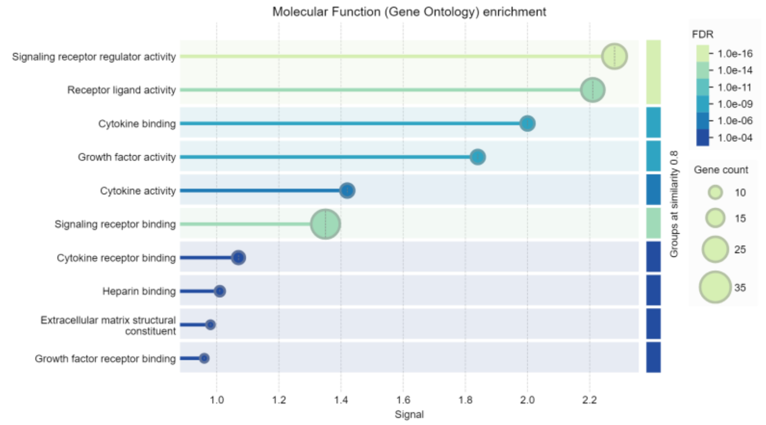

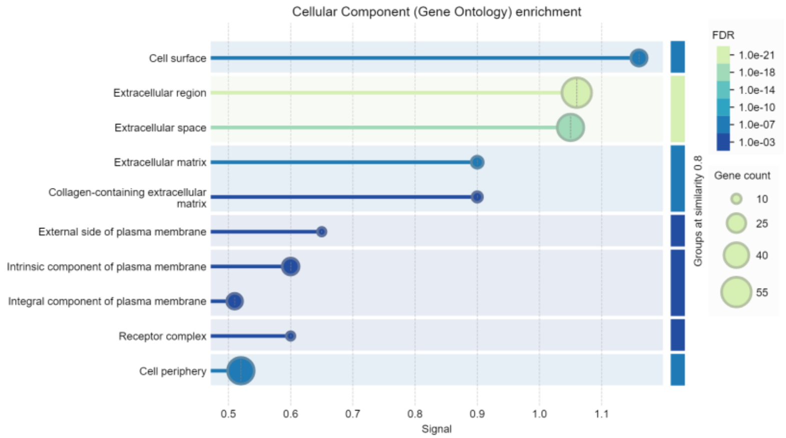

color	cluster Id	gene count	description
●	Cluster 1	69	Cytokine-cytokine receptor interaction
●	Cluster 2	16	+ Insulin-like growth factor II binding
●	Cluster 3	11	NCAM1 interactions
●	Cluster 4	7	Removal of aminoterminal propeptides from gamma-carboxylated proteins
●	Cluster 5	5	+ Age-related macular degeneration
●	Cluster 6	2	APLP1, SEZ6L
●	Cluster 7	2	HNMT, SCGB3A2
●	Cluster 8	2	Metabolism of xenobiotics by cytochrome P450
●	Cluster 9	2	CLUL1, COLEC12
●	Cluster 10	1	LILRA5
