# Supplementary material for: Plasma proteomic biomarkers as mediators or moderators for the association between poor cardiovascular health and white matter microstructural integrity: The UK Biobank study
Source: Alzheimers Dement. 2025 Jan 17;21(2):e14507. doi: 10.1002/alz.14507 (PMC11864230; doi:10.1002/alz.14507)

FIGURE S5. Summary of findings

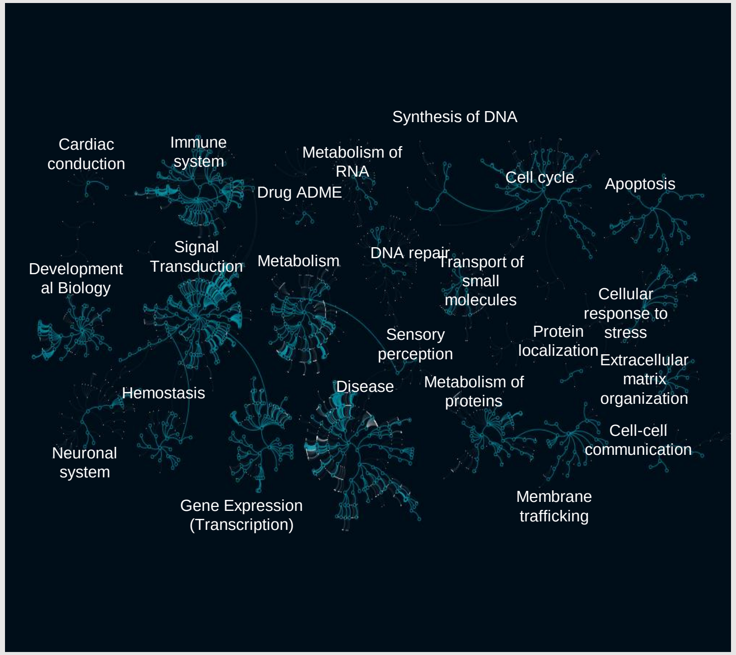

LE8z_rev vs. FA

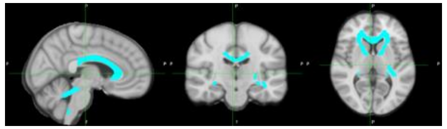

LE8z_rev vs. OD

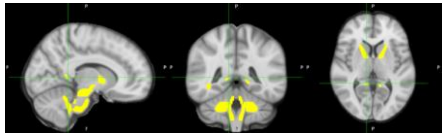

LE8z_rev vs. PROT

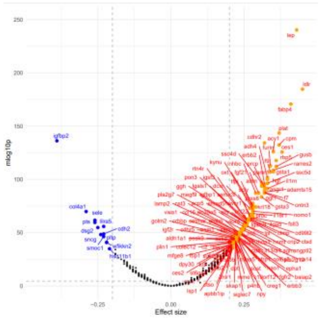

LE8z_rev→PROTselect→OD; Consistent mediators

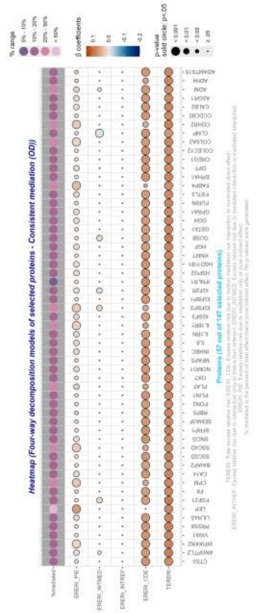

Shared consistent mediation between FA and OD:

K7=10

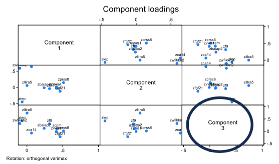

LE8z_rev→PC3→ OD

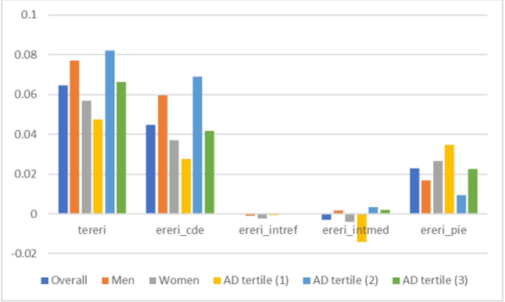

LE8z_rev,comp→PROTselect (FA and OD)

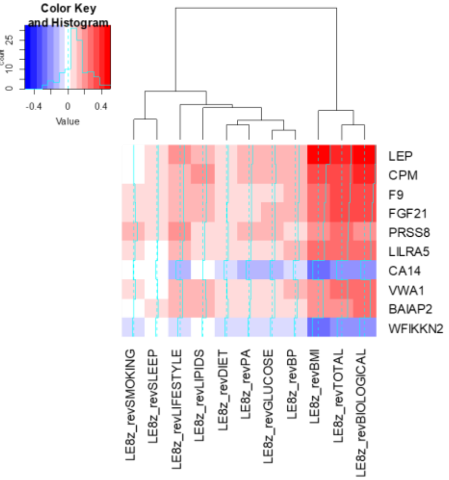

Significant TE and PIE at type I error of 0.05

K2a=212 for FA and K2b=142 for OD

Initial plasma proteins

K1=1,463

Did not survive Bonferroni correction for LE8z_rev → protein association

K3=619

Strength of the LE8z_rev→protein association is >0.20 in absolute value

K5=147

Survived Bonferroni correction for LE8z_rev→ protein association

K4=844

Total effect of LE8z_rev on FA and OD global means is significant and PIE is significant at type I error of 0.05, PIE and TE in the same direction:

K6a=14 (FA); K6b=57 (OD)

OLINK insight Pathways: FA and OD

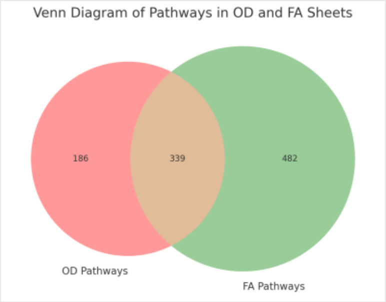

Supplement: Supplementary file 12 — Supporting information [file ALZ-21-e14507-s005.pdf]
