# Supplementary material for: Plasma proteomic biomarkers as mediators or moderators for the association between poor cardiovascular health and white matter microstructural integrity: The UK Biobank study
Source: Alzheimers Dement. 2025 Jan 17;21(2):e14507. doi: 10.1002/alz.14507 (PMC11864230; doi:10.1002/alz.14507)
Supplement: Supplementary file 13 — Supporting information [file ALZ-21-e14507-s008.pdf]

FIGURE S6. Heatmap for effect sizes of LE8 main and secondary exposures (LE8z_rev_total, lifestyle and biological sub-scores and 8 components) on selected common consistent mediators for FA_{mean} and OD_{mean} (k=10 proteins): OLS multiple linear regression models

Notes: Linear Models were adjusted for age, sex, racial minority status (Non-White vs. White), SES, household size and time elapsed from baseline assessment to neuroimaging visit. Heatmap depict the values of the point estimates (LE8 reverse coded → plasma protein) from these models, with red corresponding to positive values and blue to negative values. Values can potentially range from -1 to 1. Proteins are listed in alphabetical order. Hierarchical clustering is applied as well.

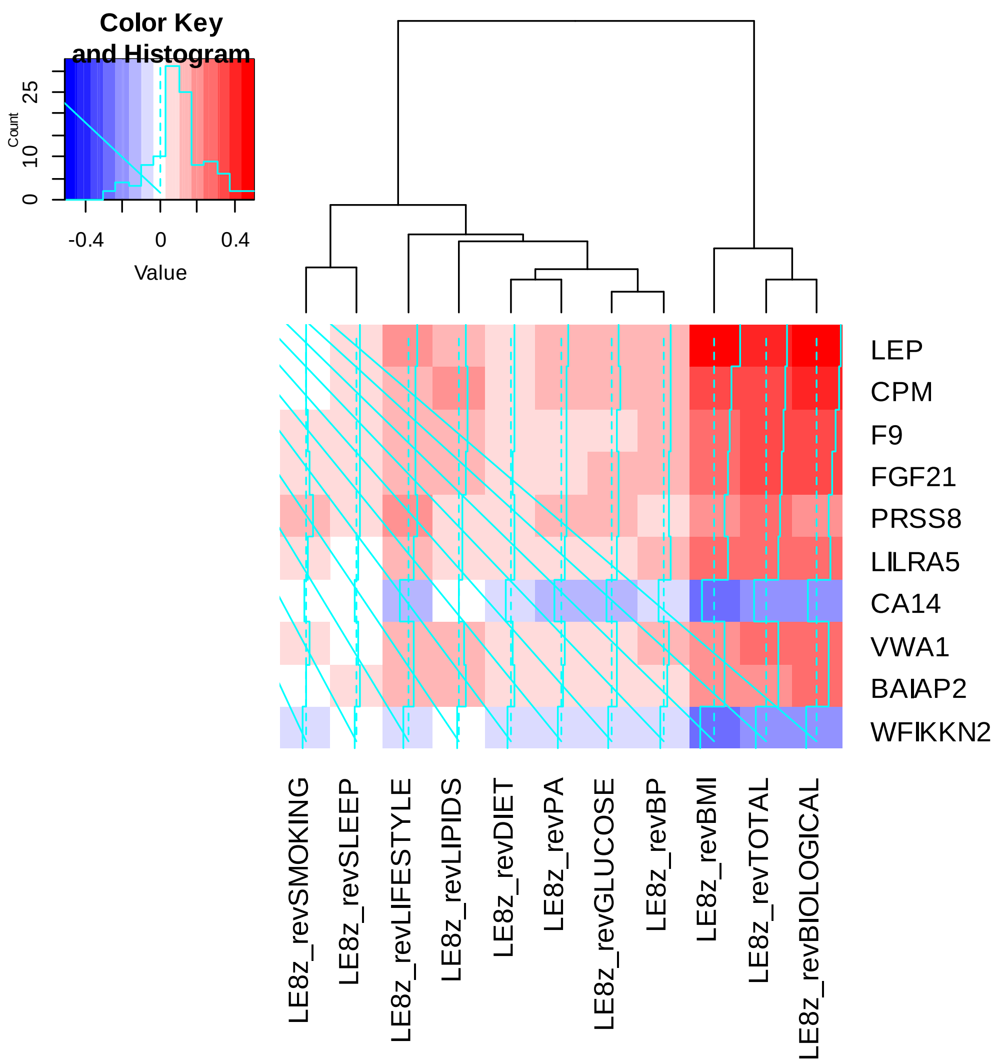
Color Key
and Histogram
Count
0
10
25
-0.4
0
0.4
Value
LEP
CPM
F9
FGF21
PRSS8
LILRA5
CA14
VWA1
BAIAP2
WFIKKN2
LE8z_revSMOKING
LE8z_revSLEEP
LE8z_revLIFESTYLE
LE8z_revLIPIDS
LE8z_revDIET
LE8z_revPA
LE8z_revGLUCOSE
LE8z_revBP
LE8z_revBMI
LE8z_revTOTAL
LE8z_revBIOLOGICAL
